# Transcription factor expression is the main determinant of variability in gene co‐activity

**DOI:** 10.15252/msb.202211392

**Published:** 2023-05-09

**Authors:** Lucas van Duin, Robert Krautz, Sarah Rennie, Robin Andersson

**Affiliations:** ^1^ Section for Computational and RNA Biology, Department of Biology University of Copenhagen Copenhagen Denmark

**Keywords:** co‐activity domains, co‐regulation, gene regulation, individual variation, transcriptional decomposition, Chromatin, Transcription & Genomics

## Abstract

Many genes are co‐expressed and form genomic domains of coordinated gene activity. However, the regulatory determinants of domain co‐activity remain unclear. Here, we leverage human individual variation in gene expression to characterize the co‐regulatory processes underlying domain co‐activity and systematically quantify their effect sizes. We employ transcriptional decomposition to extract from RNA expression data an expression component related to co‐activity revealed by genomic positioning. This strategy reveals close to 1,500 co‐activity domains, covering most expressed genes, of which the large majority are invariable across individuals. Focusing specifically on domains with high variability in co‐activity reveals that contained genes have a higher sharing of eQTLs, a higher variability in enhancer interactions, and an enrichment of binding by variably expressed transcription factors, compared to genes within non‐variable domains. Through careful quantification of the relative contributions of regulatory processes underlying co‐activity, we find transcription factor expression levels to be the main determinant of gene co‐activity. Our results indicate that distal *trans* effects contribute more than local genetic variation to individual variation in co‐activity domains.

## Graphical abstract

Human individual variation is used to characterize the co‐regulatory processes underlying domain co‐activity. Systematic quantification of their effect sizes reveals transcription factor expression as the main determinant of co‐activity.

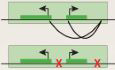

## Introduction

Gene expression is the integrated result of multiple gene regulatory processes acting at scales ranging from local binding of transcription factors (TFs) at regulatory elements (Spitz & Furlong, 2012; Lambert *et al*, 2018; Andersson & Sandelin, 2020) to permissive chromatin environments ensured by large‐scale chromatin topologies and histone post‐translational modifications (PTMs) (Robson *et al*, 2019; Schoenfelder & Fraser, 2019). Aberrant gene activity may thus result from genetic variants causing alterations in any of such regulatory processes. Disentangling the regulatory mechanisms acting upon each gene in its native context is therefore crucial for understanding the basis of transcriptional regulation and, ultimately, the role of dysregulation in disease.

Groups of genes expressed in a cell are often co‐regulated, in that they are regulated by the same regulatory processes, for example, having a common set of TFs binding their promoters or enhancers or even having shared distal enhancers, thereby ensuring coordinated transcription, referred to as co‐expression, in foci with high TF concentration (Robson *et al*, 2019; Pachano *et al*, 2022). Similarly, co‐regulation through shared localization within domains of permissive or repressive histone PTMs ensures accurate coordinated activation or repression for multiple genes during development (Coleman & Struhl, 2017; Zenk *et al*, 2017). Analysis of co‐expression can therefore yield insights into the regulatory processes acting on genes through co‐regulation.

Co‐expression is typically measured by quantifying the correlation between expression levels of gene pairs across cell types and/or conditions (Hawrylycz *et al*, 2012), but can also be inferred from coordinated differential expression within genomic domains between cell types or conditions (Zufferey *et al*, 2021). Complex regulatory networks involving multiple genes can also be learned from expression data. Assessment of the downstream effects of perturbations of these networks helps to identify important regulatory processes and pathways implicated in disease. More recently, single cell‐based approaches have presented a fine resolution picture of coordinated activities of genes across individual cells (Crow & Gillis, 2018; preprint: González‐Blas *et al*, 2022; He *et al*, 2022).

While analysis of gene regulatory differences between cell types may successfully capture differential activity between TFs or domain repression, studying variation in gene expression between individuals within the same cell type may reveal other regulatory patterns. Within the same cell type, a major determinant of co‐expression is genomic proximity (Kustatscher *et al*, 2017), suggesting that data across individuals for the same cell type might better reveal the regulatory activities underlying co‐regulation in the absence of strong cell type‐specific differences. Topologically associating domains (TADs) have been suggested to confine interactions between regulatory elements within genomic loci (Symmons *et al*, 2014) and insulate repressed genes from active domains (Narendra *et al*, 2015). However, while deletions of TAD boundaries or chromosomal inversions may disrupt TAD‐contained regulatory wirings and cause gene dysregulation (Gröschel *et al*, 2014; Lupiáñez *et al*, 2015; Laugsch *et al*, 2019), the proper formation of TADs only has a marginal contribution to gene expression (Nora *et al*, 2017; Rao *et al*, 2017; Ghavi‐Helm *et al*, 2019). This is reflected by only a minor agreement between gene co‐regulation and TAD co‐localization across active genes (Soler‐Oliva *et al*, 2017; Zufferey *et al*, 2021).

Quantifying co‐regulatory effect sizes is complicated by the likelihood that certain regulatory processes may primarily control coordinated expression activities, while other regulatory processes may have a larger influence on the expression level of individual genes. For instance, two proximal genes may be co‐regulated leading to co‐activity, but their expression levels may differ due to differences in their promoter strength or local chromatin environment. Decoding transcriptional regulation thus requires an accurate quantification of both the effect sizes of regulatory processes acting on individual genes and those driving co‐activity. To this end, we recently developed an approach to decompose RNA expression levels across chromosomes into two parts: an expression component related to genomic positioning, and a location‐independent component (Rennie *et al*, 2018). The position‐dependent component accurately captures domains of chromatin compartments and their activities and reveals large‐scale co‐activity patterns between neighboring genes, indicating a sizable effect of regulatory processes modulating co‐activity in genomic neighborhoods.

Here, we make use of the transcriptional decomposition approach (Rennie *et al*, 2018) to investigate and quantify the changes in regulatory processes underlying variability in co‐activity in a genotyped panel of lymphoblastoid cell lines profiled by RNA‐seq (Lappalainen *et al*, 2013). We identify domains of co‐active genes and show that gene co‐activity on the domain level is largely invariable between individuals. We then focus specifically on sub‐domains exhibiting high individual variation, in order to characterize the regulatory processes influencing their co‐activity. We find that variability in co‐activity largely reflects histone PTM variation and that genes contained within variable co‐activity domains have a higher sharing of eQTLs, a higher number and variability of interactions with enhancers, and are enriched in specific TF binding sites, which are bound by more variably expressed TFs. Finally, in an attempt to quantify the combined effects of regulatory processes underlying co‐activity, we find that the expression levels of TFs explain on average more of the observed variation in co‐activity at variable domains than local genetic variation or interactions. Our study thus highlights TF expression as the main determinant of gene co‐activity, which has implications for continued efforts in characterizing the role of transcriptional dysregulation in disease.

## Results

### Transcriptional decomposition captures positionally dependent gene co‐activities

We have previously established transcriptional decomposition (Rennie *et al*, 2018), which is a novel Bayesian modeling‐based approach for decomposing RNA expression in genomic bins along chromosomes into two parts: the portion of expression attributable to the local genomic neighborhood (positionally dependent [PD] component, referred to henceforth as “co‐activity” in the current study), and the portion of expression independent of the genomic position (positionally independent [PI] component). Formally, we model the log of the normalized (reads per million, RPM) expression of a given genomic bin as approximately PD + PI, where the value for PD (co‐activity) is assumed to be dependent on neighboring bins, in contrast to the value for PI, which is assumed to be independent of its neighbors. The co‐activity portion is thus highly similar at close‐by regions, which we hypothesize could result from a combination of shared regulatory mechanisms as well as similarities in the underlying chromatin environment, while the independent portion is uncorrelated with distance and likely reflects gene‐specific regulatory mechanisms (Rennie *et al*, 2018).

**Figure 1:**
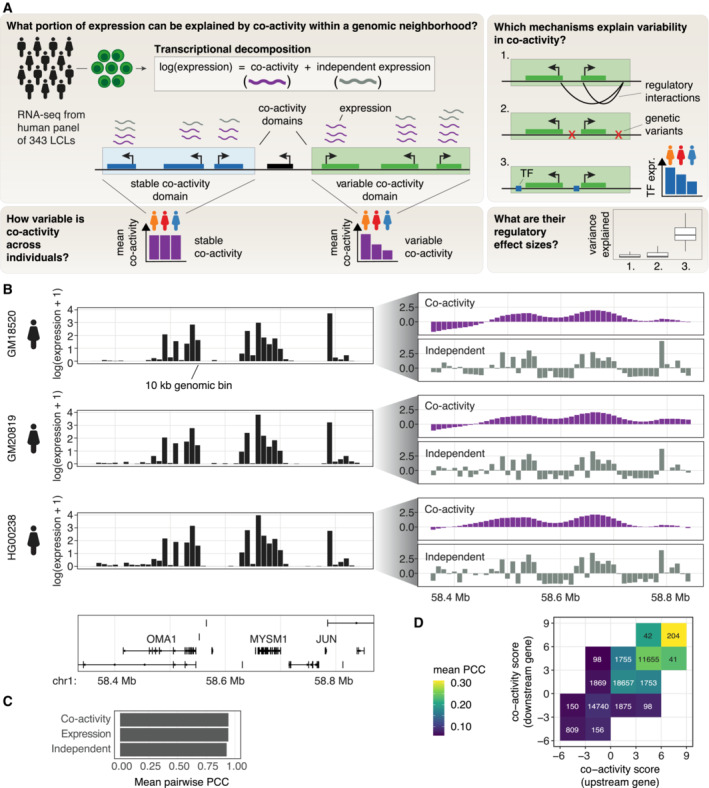
Transcriptional decomposition as a basis for modeling gene co‐activity and its regulatory determinants ASchematic illustrating how RNA expression can be attributed both to co‐activity and positionally independent mechanisms. Positionally dependent co‐activity between proximal genes forms co‐activity domains, while genes with no or only independent activities are contained outside of domains. We here focus specifically on co‐activity domains with variable co‐activity between individuals to study the regulatory mechanisms driving co‐activity, including genotype, TF abundance, and regulatory interactions.BOverall strategy of how expression data from each sample (individual LCLs, biological replicates) is decomposed into transcriptional components. Via approximate Bayesian modeling, normalized RNA expression count data (reads per million, RPM), quantified in 10 kb genomic bins (shown left for three individuals), is modeled as approximately PD + PI, where the value for PD (positionally dependent component: co‐activity) is assumed to be dependent on neighboring bins, whereas the value for PI (positionally independent component) is assumed to be independent of its neighbors (shown right). The co‐activity (PD) component is modeled as a first‐order random walk (see Materials and Methods).CAverage Pearson correlation coefficient (PCC) between all pairs of considered LCLs, for co‐activity score, expression, and positionally independent component.DMean co‐expression (PCC) of neighboring gene pairs, stratified by the co‐activity score of the upstream gene (horizontal axis) and the downstream gene (vertical axis) in each pair. The number of gene pairs considered are stated in each tile, tiles with fewer than 10 gene pairs were omitted from the plot.

We reasoned that we could leverage this modeling approach to obtain co‐activity in order to investigate individual variation in regulatory processes underlying the co‐regulated expression of genes (Fig 1A). To this end, we made use of RNA‐seq data from lymphoblastoid cell lines (LCLs) derived from a panel of 343 individuals from four European and one African populations (Lappalainen *et al*, 2013; Dataset EV1).

**Figure EV1:**
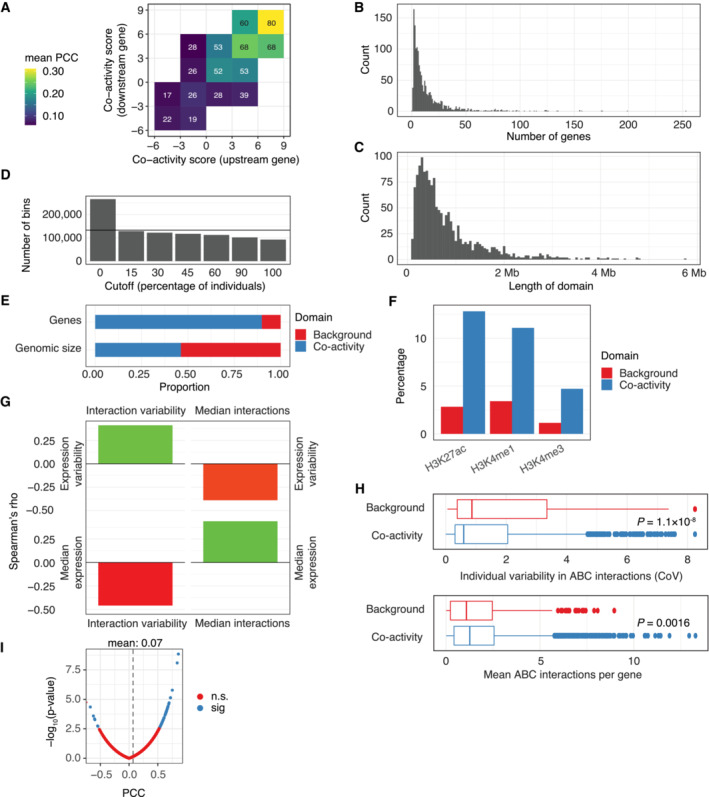
Comparison of co‐activity domains versus background regions ACo‐expression (PCC) of neighboring gene pairs, stratified by the co‐activity score of the upstream gene (horizontal axis) and the downstream gene (vertical axis) in each pair. Tiles are labeled by the percentage of correlated gene pairs (Pearson correlation test, BH‐adjusted *P* value < 0.1).BHistogram of the number of genes per co‐activity domain.CHistogram of co‐activity domain sizes.DThe number of 10 kb genomic bins in co‐activity domains, for different cutoffs based on the percentage of individuals showing a co‐activity score above zero. The horizontal line indicates half of all bins in the genome.EProportion of expressed genes (TPM > 0.1, *n* = 25,982) and genome size in co‐activity domains and background regions.FPercentage of 10 kb bins showing significant (Pearson correlation test, BH‐adjusted *P* < 0.05) correlation between co‐activity score and histone PTM signal in co‐activity domains and background regions.GRelation between ABC‐predicted interactions and expression, in terms of variability and level. Shown are Spearman's rho correlation values for (clockwise) the expression variability and the interaction variability, the expression variability and the number of interactions, the amount of expression and the number of interactions, and the amount of expression and the variability of interactions, per gene. Levels are median, variabilities Coefficient of Variation (CoV).HComparison of variability and number of ABC‐predicted interactions per gene in co‐activity domains and background regions. Mann–Whitney *U*‐test *P*‐values are shown. For box‐and‐whiskers, central band denotes the median, hinges the first and third quartiles, and the whiskers extend max 1.5 × IQR from the hinges.IPCC (horizontal axis) versus Pearson's correlation test *P*‐value (vertical axis) for gene expression versus the number of ABC‐derived gene interactions per individual. Color indicates an BH‐adjusted *P*‐value of < 0.1 (n.s., non‐significant; sig, significant).

To capture positional dependencies influencing gene activity and investigate how these vary across individuals, we applied transcriptional decomposition of expression data in each LCL using aggregated expression in 10 kb tiled windows of the genome (Fig 1B) and extracted the co‐activity scores, as captured by the positionally dependent component. In general, the resulting co‐activity scores exhibited strong inter‐individual correspondence (mean pairwise Pearson correlation coefficient [PCC]: 0.94; Fig 1C), akin to the resemblance between cell types (Rennie *et al*, 2018). As expected, gene pairs that had a higher and more similar co‐activity score tended to have higher co‐expression, as measured by the mean PCC across LCLs, than those with low or dissimilar co‐activity scores (Figs 1D and EV1A).

In agreement with previously observed similarities between RNA‐seq and Cap Analysis of Gene Expression (CAGE)‐derived transcriptional components of GM12878 (Rennie *et al*, 2018), we observed that the co‐activity derived from different assays correlated better between individuals of the same cell type (LCL GM12872 RNA‐seq versus LCL GM12878 CAGE, PCC = 0.8) than between cell types (LCL GM12872 RNA‐seq versus HeLa or HepG2 CAGE, PCC = 0.73 and 0.73, respectively) (Appendix Fig S1). This demonstrates that co‐activities derived from RNA‐seq reflect those of CAGE. It further indicates that cell type‐specific regulatory activities are reflected by changes in co‐activity and that these can be captured by transcriptional decomposition of RNA‐seq data.

Taken together, we conclude that transcriptional decomposition of RNA‐seq data reveals co‐activity, indicating that its application to human panels may reveal the genetic basis of variation in gene co‐activity.

### Positional dependencies of expression reveal co‐activity domains of shared regulation

**Figure 2:**
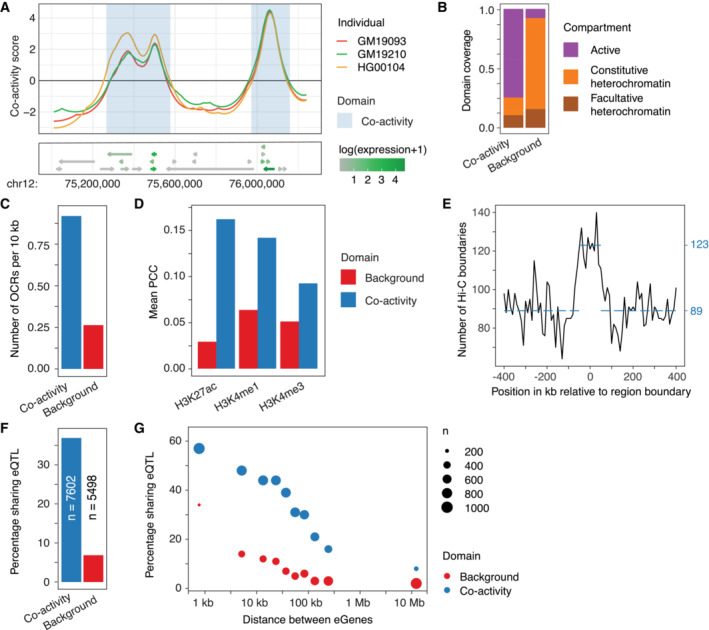
Positional dependencies of expression reveal co‐activity domains of shared regulation ATop: co‐activity scores at locus chr12:75,000,000‐76,250,000 for three individuals. Highlighted in blue are co‐activity domains determined by positive co‐activity scores in at least 15% of considered LCLs. Bottom: gene track showing the location and directions (arrows) of genes, and their expression level (color).BProportion of co‐activity domains and background regions (derived from regions with negative co‐activity scores) in active euchromatin, and constitutive and facultative heterochromatin compartments.CNumber of ATAC‐seq‐inferred open chromatin regions (OCRs) in co‐activity domains and background regions.DMean PCC between co‐activity scores and histone PTM signal values in 10 kb bins in co‐activity domains and background regions.ENumber of Hi‐C‐derived TAD boundaries (vertical axis) at positions relative to co‐activity domain boundaries (horizontal axis). Dotted lines show the mean number of TAD boundaries less and more than 50 kb from a co‐activity domain boundary.FPercentage of neighboring eGene pairs sharing at least one eQTL, for pairs contained within co‐activity domains and pairs outside of co‐activity domains. *n* indicates the number of gene pairs considered.GeQTL sharing of neighboring eGene pairs over distance, summarized in bins with an equal number of genes. The dot size indicates the number of gene pairs in the bin (*n*). For all distances, the difference between background and co‐activity domains was highly significant (*P* < 1 × 10^−5^, Fisher's exact test).

Since we observed stronger co‐expression among pairs of genes associated with a positive co‐activity score (Fig 1D), we reasoned that the sign of the co‐activity score could be used to define domains of shared transcriptional regulation between genes influencing their co‐activity (referred to as co‐activity domains, see Fig 2A for an example locus). We defined co‐activity domains as genomic regions having a positive sign in co‐activity in at least 15% of individuals and containing at least two expressed annotated genes. Subsequent merging of proximal domains resulted in a set of 1,489 co‐activity domains (Dataset EV2; median domain length: 570 kb; median number of active genes per domain: 8; Fig EV1B and C), noting that the genomic size of domains appears robust to the percentage of individuals considered in the calculation (Fig EV1D). These domains contained the majority (88%) of expressed genes in LCLs, in agreement with previous results (Rennie *et al*, 2018), and spanned 44% of the human genome (Fig EV1E).

To characterize properties of derived co‐activity domains, we first investigated whether the domains reflected chromatin states. Indeed, active chromatin compartments (Rao *et al*, 2014) accounted for 75% of the total genomic size of co‐activity domains, compared to only 10% of background regions with negatively signed co‐activity scores. In contrast, heterochromatin compartments made up 75% of background regions and 15% of co‐activity domains (Fig 2B). In support, co‐activity domains were associated with almost four times more open chromatin regions than what was observed for background regions (0.93 versus 0.27 ATAC‐seq open chromatin sites per 10 kb, respectively) (Fig 2C). In general, the correlation between co‐activity score and activating histone modifications was higher in co‐activity domains than in background regions of negative co‐activity scores (Fig 2D). The genomic proportion showing a correlation (Pearson correlation test; Benjamini–Hochberg [BH] adjusted *P* < 0.1) was also higher in co‐activity domains than in background regions (Fig EV1F), when compared across 79 individuals with associated histone ChIP‐seq data (Grubert *et al*, 2015).

We reasoned that the enrichment in physical contacts within TADs should be reflected by the observed co‐activity domains. In agreement with previous observations (Rennie *et al*, 2018), we observed that TAD boundaries (Rao *et al*, 2014; Dekker *et al*, 2017) were enriched at boundaries of co‐activity domains (Fig 2E, Fisher's exact test, *t* = 1.5, *P* = 1.5 × 10^−5^). These results are further supported by a higher expression correlation between neighboring active genes within co‐activity domains compared to gene pairs outside of domains (PCC: 0.30 and 0.20, respectively).

Next, we asked how genes located within co‐activity domains compared to genes outside of domains with respect to their regulatory interactions. To this end, we applied activity‐by‐contact (ABC) modeling (Fulco *et al*, 2019) to predict regulatory interaction maps for 79 of the 343 individuals with available chromatin accessibility (Degner *et al*, 2012; Gorkin *et al*, 2019) and H3K27ac (Grubert *et al*, 2015; Gorkin *et al*, 2019) data. Using these predictions, we observed an association between the median number of associated enhancers and the median expression of genes across individuals (Spearman's rho 0.440, *P* < 2 × 10^−16^; Fig EV1G), with multi‐enhancer genes having a higher expression than those with few or no predicted enhancers. In addition, genes within co‐activity domains were generally less variable in their number of ABC‐associated enhancers (*P* = 1.1 × 10^−8^; Mann–Whitney *U*‐test; Fig EV1H). This is likely in part explained by their association with higher gene expression levels, since more variably interacting genes had lower median expression across individuals (Spearman's rho −0.46; *P* < 2 × 10^−16^; Fig EV1G). Similarly, genes located in co‐activity domains were on average associated with more enhancers than those in background regions (1.8 versus 1.6 connections per gene on average, respectively; *P* = 0.0016, Mann–Whitney U‐test; Fig EV1H), although we cannot rule out that these differences are driven by low H3K27ac signal in background regions.

We next asked whether the number of interactions per gene per individual was associated with the gene expression of that individual (paired analysis). Overall, the expression of a gene across individuals showed no or only weak correlation with their corresponding number of ABC‐connections (PCC = 0.07, Fig EV1I). This might reflect ABC measures being influenced by noise in the input data, or that enhancers might relate to target gene expression in ways beyond their raw numbers. For instance, multiple enhancers for a gene may provide regulatory redundancy (Perry *et al*, 2010; Joshua & Payne, 2015) and, hence, not additively influence gene expression levels. Indeed, the expression of only 62 genes showed correlation with the number of interactions (Pearson correlation test; BH‐adjusted *P* < 0.1, Fig EV1I), for which the correlation sign was positive in 90% of cases. Together, these results suggest that the number of regulatory interactions may reflect differences in expression level within a co‐activity domain across genes, as previously shown (Andersson *et al*, 2014), but that only few regulatory domains are sensitive to perturbations at an individual level.

Since genes within the same co‐activity domain are assumed to share regulation, we investigated the co‐operative capacity of expression quantitative trait loci (eQTL) on genes within the same domain. We focused on genes associated with at least one eQTL (eGenes). Overall, we found that 38% of neighboring eGene pairs contained within the same co‐activity domain shared at least one eQTL, compared to only 7% of eGene pairs located outside of co‐activity domains (Fig 2F). Furthermore, this result could not be explained by differences in the distances between eGene pairs inside or outside of co‐activity domains. In fact, we observed a significant enrichment (Fisher's exact test, *P* < 1 × 10^−5^) of shared eQTLs among eGene pairs in co‐activity domains even when only considering pairs at least 100 kb apart (Fig 2G).

Taken together, these results indicate that co‐activity domains are capturing local neighborhoods of genes, whose collective output is influenced by an environment enriched in regulatory interactions, permissive chromatin, and the co‐operative effects of local sequence variants.

### Expression variation across individuals uncovers regulatory mechanisms underlying co‐activity

**Figure 3:**
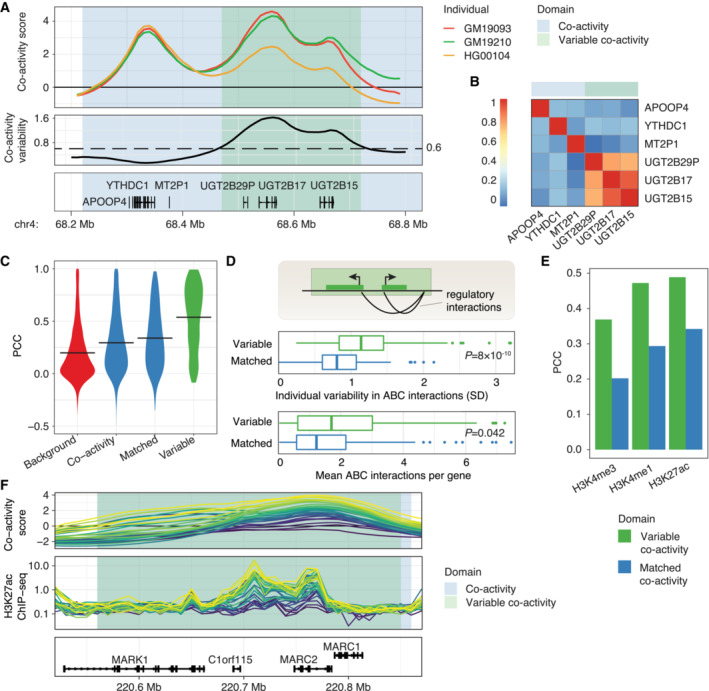
Variable co‐activity domains reveal individual variability in chromatin states and regulatory interactions ATop: co‐activity scores for three individuals (GM19093, GM19210, HG00104) along a co‐activity domain (chr4:68,200,000‐68,800,000). Middle: individual variability (standard deviation) of co‐activity scores across the co‐activity domain. The horizontal line shows the considered threshold (0.6) for calling variable co‐activity domains. Bottom: gene locations in the co‐activity domain.BCo‐expression (PCC) of genes contained within the co‐activity domain depicted in (A).CViolin plots of neighboring gene pair co‐expression (PCC), in background regions, all and matched co‐activity domains, and variable co‐activity domains. Mean PCCs per domain class are depicted by horizontal lines.DTop: variability in gene interactions could lead to variability in co‐activity level. Comparison of variability (middle) and number (bottom) of interactions in variable co‐variability domains compared to matched non‐variable co‐variability domains. Mann–Whitney *U*‐test *P*‐value is shown. For box‐and‐whiskers, central band denotes the median, hinges the first and third quartiles, and the whiskers extend max 1.5 × IQR from the hinges.ECorrelation (PCC) between average co‐activity score and average histone PTM signal for each variable co‐activity domain and matched non‐variable co‐activity domain.FExample locus (chr1:220,500,000‐220,900,000) showing co‐activity score (top) and H3K27ac histone PTM signal (RPM, middle). Individuals share color between upper and middle panels. Co‐activity and variable co‐activity domains are highlighted. Bottom: gene track.

**Figure EV2:**
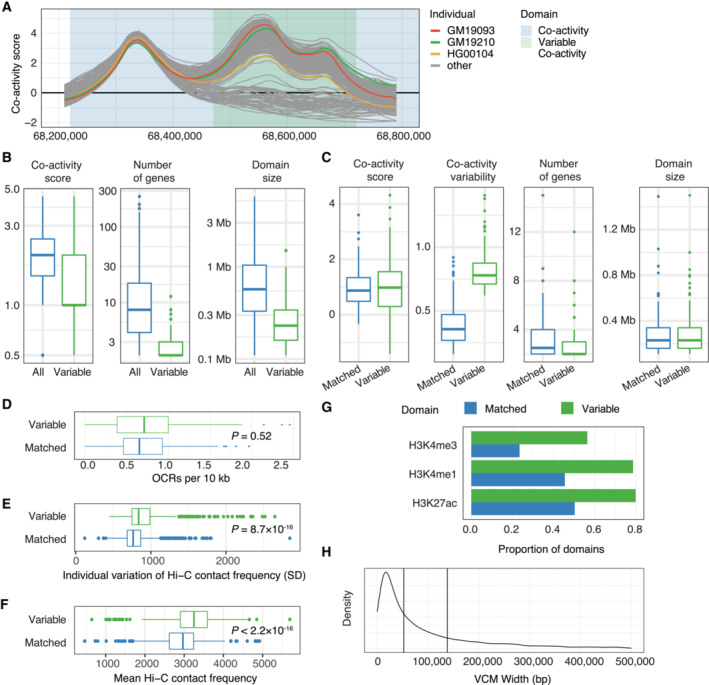
Comparison of variable co‐activity domains versus non‐variable co‐activity domains ACo‐activity scores for all considered individuals in a region containing a variable co‐activity domain (chr4:68,200,000‐68,800,000).BComparison of co‐activity score, number of contained genes and domain size of variable and all non‐variable co‐activity domains. For box‐and‐whiskers, central band denotes the median, hinges the first and third quartiles, and the whiskers extend max 1.5 × IQR from the hinges.CComparison of co‐activity score, variability, number of contained genes and domain size of variable and matched non‐variable co‐activity domains. Box‐and‐whiskers defined as in panel B.DNumber of ATAC‐seq‐inferred open chromatin regions (OCRs) per 10 kb in variable and matched non‐variable co‐activity domains. Mann–Whitney *U*‐test *P*‐value is shown. Box‐and‐whiskers defined as in panel (B).EVariability (standard deviation) in Hi‐C‐derived interaction frequencies of 50 kb bins overlapping annotated gene TSSs summed across bins within 1 Mb in variable and matched non‐variable co‐activity domains. Mann–Whitney U‐test *P*‐value is shown. Box‐and‐whiskers defined as in panel (B).FAverage Hi‐C‐derived interaction frequencies of 50 kb bins overlapping annotated gene TSSs summed across bins within 1 Mb in variable and matched non‐variable co‐activity domains. Mann–Whitney *U*‐test *P*‐value is shown. Box‐and‐whiskers defined as in panel (B).GProportion of variable and matched non‐variable co‐activity domains showing a correlation (Pearson correlation test, BH‐adjusted *P*‐value < 0.1) between average co‐activity score and average ChIP‐seq histone PTM levels per domain.HDensity plot of VCM sizes (median: 52 kb, first vertical line; mean: 138 kb, second vertical line). 523 VCMs (~ 5%) surpassing the max considered size of 500 kb (VCM max width: 24 Mb) are excluded from the plot.

In general, we observed a strong conformity in the positional co‐activity scores across all individuals, but noted the presence of sub‐regions within co‐activity domains displaying considerable variation between individuals (Figs 3A and EV2A). Accordingly, and as a basis for understanding regulation of co‐activity, we focused on regions that differed in co‐activity between individuals, and thus presumably in the activity of their underlying shared regulatory mechanisms. We characterized genomic regions within co‐activity domains involving two or more expressed genes and showing high variability (standard deviation > 0.6) in their average co‐activity scores across the panel of individuals. This identified a total of 212 genomic regions, which we refer to as variable co‐activity domains (Dataset EV3). For example, we detected considerable variation in co‐activity scores in the variable co‐activity domain containing UDP glucuronosyltransferase genes *UGT2B15* and *UGT2B17* and pseudogene *UGT2B29P* (shown in Fig 3A for 3 individuals, and Fig EV2A for all individuals). These three genes correlated in their expression more strongly than the other genes, *APOOP4*, *YTHDC1*, *MT2P1*, in the encompassing co‐activity domain (Fig 3B). Indeed, the observation that genes in variable co‐activity domains are co‐expressed to a higher degree held genome‐wide. For comparative purposes, we sampled a matched set of non‐variable co‐activity domains from the whole set of co‐activity domains, which did not overlap with the set of variable co‐activity domains, but displayed similar mean co‐activities, genomic sizes and gene numbers (Dataset EV4; Materials and Methods; Fig EV2B and C). In general, neighboring gene pairs in variable co‐activity domains were more co‐expressed (PCC of gene expression across LCLs; Fig 3C) than genes contained in the matched non‐variable domains, suggesting a shared regulation of genes within variable co‐activity domains with an effect size stronger than that of non‐variable co‐activity domains.

Next, we investigated which mechanisms were associated with individual variability in variable co‐activity domains. We hypothesized that inter‐individual variation could be associated with at least four (non‐orthogonal) gene regulatory inputs: histone modifications, TF binding, enhancer‐gene interactions, and genetic variants.

We first asked if the average co‐activity score in variable co‐activity domains was reflective of the chromatin state across individuals. Interestingly, the number of open chromatin regions did not differ significantly between the variable and matched non‐variable co‐activity domains (Fig EV2D). Still, genes in variable co‐activity domains had 20% more ABC‐predicted interactions (*P* = 8 × 10^−10^; Mann–Whitney *U*‐test), as well as a 20% higher interaction variability (*P* = 0.042; Mann–Whitney *U*‐test, Fig 3D) compared to matched non‐variable co‐activity domains, accounting for differences in underlying domain activity. In support, genes in variable co‐activity domains had both higher (*P* < 2.2 × 10^−16^; Mann–Whitney *U*‐test) and more variable (*P* = 8.7 × 10^−16^; Mann–Whitney *U*‐test) chromatin interaction frequencies, as inferred from Hi‐C data (Gorkin *et al*, 2019), than genes in matched non‐variable co‐activity domains (Fig EV2E and F). Overall, the average co‐activity scores in variable co‐activity domains correlated strongly with H3K27ac, H3K4me1, and H3K4me3, and these correlations were more pronounced than in the set of matched non‐variable co‐activity domains (Fig 3E), and more likely to be statistically significant (Fig EV2G). The association between variability in histone modification levels and variability in positional co‐activity scores is clear in the variable co‐activity domain at the *MARC1‐2* gene locus (Fig 3F), showing that H3K27ac matches both the variance and rank of co‐activity scores across individuals.

Domains of co‐varying histone modifications have previously been described as variable chromatin modules (VCMs) (Waszak *et al*, 2015) and cis‐regulatory domains (CRDs) (Delaneau *et al*, 2019). These modules/domains, which are on average shorter than the variable co‐activity domains described here (Median 52 kb, mean 138 kb; Fig EV2H), showed a high enrichment in variable co‐activity domains (odds ratio 6; Fisher's exact test, *P* < 2.2 × 10^−6^). In fact, VCMs accounted for as much as 85% coverage of variable co‐activity domains, indicating that variable chromatin domains are capturing the same variability, although through smaller domains. This further demonstrates that variation in co‐activity can be derived from orthogonal assays, including those measuring histone modifications. Thus, it appears that chromatin state reflects co‐activity domain variability.

**Figure 4:**
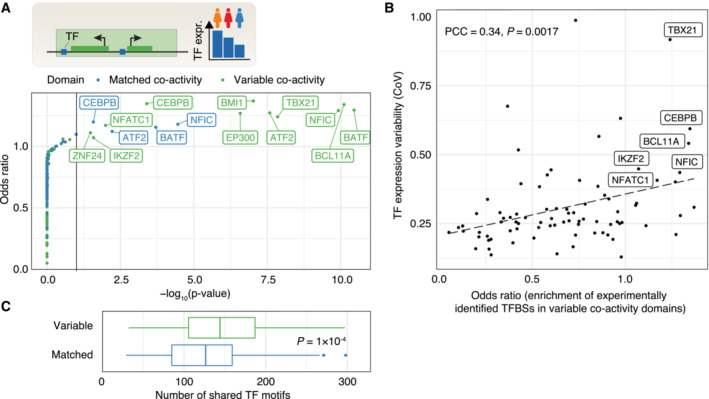
TF expression variability and binding differences influence co‐activity variability ATop: variability in TF expression could lead to variability in co‐activity level. Bottom: enrichment (odds ratio) of experimentally identified TFBSs in variable and matched non‐variable co‐activity regions versus all non‐variable co‐activity regions.BEnrichment of experimentally identified TFBSs in variable regions (odds ratio, horizontal axis) and expression variability (CoV, vertical axis) for each considered TF. PCC and Pearson correlation test *P*‐value are shown. TFs both being among the top 10 enriched and the top 10 variably expressed are labeled.CNumber of JASPAR predicted TFBSs shared in all ATAC‐seq‐inferred OCRs in variable and matched non‐variable co‐activity domains. Mann–Whitney *U*‐test *P*‐value is shown. For box‐and‐whiskers, central band denotes the median, hinges the first and third quartiles, and the whiskers extend max 1.5 × IQR from the hinges.

**Figure EV3:**
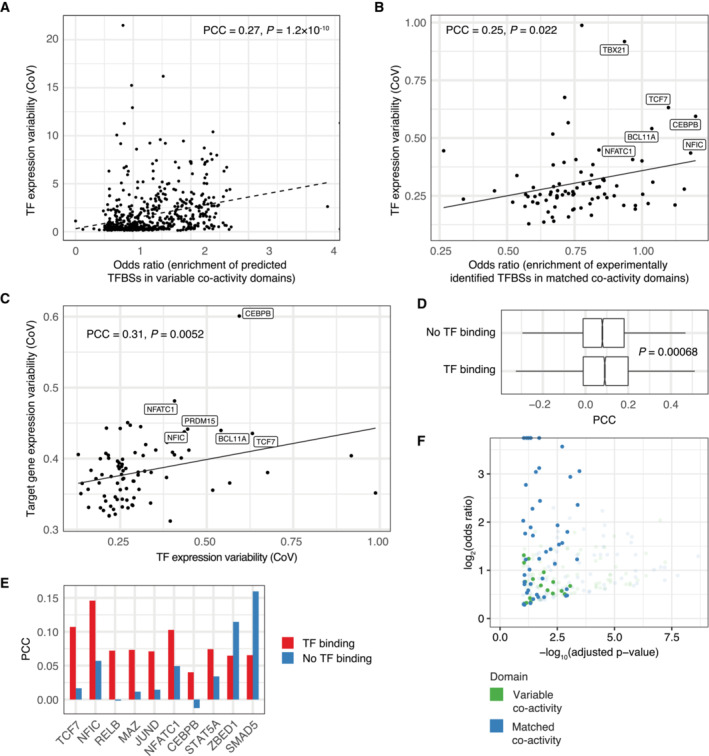
Transcription factor variability and binding differences versus co‐activity variability AEnrichment of predicted TFBSs in variable regions (odds ratio, horizontal axis) and expression variability (CoV, vertical axis) for each considered TF. PCC and Pearson correlation test *P*‐value are shown.BEnrichment of experimentally defined TFBSs (odds ratio, horizontal axis) in matched non‐variable co‐activity domains (all co‐activity domains as background) versus TF expression variability (CoV, vertical axis). PCC and Pearson correlation test *P*‐value are shown.CTF expression variability (CoV, horizontal axis) versus variability of TF target genes (CoV, vertical axis). Each dot represents a TF, vertical axis value the mean CoV over all genes containing an ENCODE TFBS in their promoters. PCC and Pearson correlation test *P*‐value are shown.DCorrelation (PCC) between TF expression and co‐activity score for variable co‐activity domains for which there are no identified TFBSs compared to variable co‐activity domains with identified TFBSs, across all TFs and variable domains. Mann–Whitney *U*‐test *P*‐value is shown. For box‐and‐whiskers, central band denotes the median, hinges the first and third quartiles, and the whiskers extend max 1.5 × IQR from the hinges.ECorrelation (PCC) between TF expression and co‐activity score for variable co‐activity domains with identified TFBSs compared to variable co‐activity domains for which no TFBSs were identified, for 10 TFs showing differences in PCC (Welch Two‐Sample *t*‐test, BH‐adjusted *P* < 0.1).FEnrichment of predicted TFBSs in promoter regions (−2000 to +200 around annotated TSSs) of genes in variable and matched non‐variable co‐activity domains (log_2_ odds ratio, vertical axis) and associated significance (−log_10_(BH‐adjusted *P*‐value), horizontal axis).

Another possible explanation for the variation in variable co‐activity domains is the variable expression of TFs preferentially binding to regulatory elements in these domains. Of 83 tested TFs (Dataset EV5) with experimentally identified binding sites in LCLs (Dunham *et al*, 2012), we found that TF binding sites (TFBSs) for NFATC1, CEBPB, EP300, BMI1, ATF2, TBX21, NFIC, BCL11A, and BATF were enriched in open chromatin regions of variable co‐activity domains compared to open chromatin regions in the wider set of co‐activity domains (Fisher's exact test, BH‐adjusted *P* < 0.1; Fig 4A). Of these, TFBSs for BATF and NFIC were also enriched, although to a lesser extent, in the matched non‐variable co‐activity domains. This can possibly be explained by variable and matched non‐variable co‐activity domains sharing certain properties (e.g., mean co‐activity score, number of genes, and domain size; Fig EV2C). Among the identified TFs associated with domain variability, we find both those that are expressed across multiple cell types and tissues (EP300, BMI1, ATF2, NFIC) and those that are immune‐cell related (NFATC1, CEBPB, TBX21, BCL11A, BATF). Two of the latter TFs, TBX21 and BATF, further show LCL‐biased expression across cell types and tissues (The GTEx Consortium, 2020), suggesting that their association with co‐activity domains and the variability at these domains may be cell type‐specific. Interestingly, the more enriched the TFBSs for a given TF were within variable co‐activity domains, the more variable we observed the expression of the TF itself to be (PCC: 0.34, Pearson correlation test, *P* = 0.0017, Fig 4B). The same pattern was observed for predicted TFBSs derived from TF motif scanning (Castro‐Mondragon *et al*, 2022; PCC: 0.27, Pearson correlation test, *P* = 1.2 × 10^−10^; Fig EV3A), and for experimentally defined TFBSs for the matched non‐variable co‐activity domains (PCC: 0.25, Pearson correlation test, *P* = 0.022; Fig EV3B), although the associations were weaker. One possible reason for this relationship could be that more variably expressed genes tend to be bound by more variably expressed TFs (PCC: 0.31, Pearson correlation test, *P* = 0.0052, Fig EV3C). Notably, CEBPB, BCL11A, NFATC1, and NFIC, which were enriched specifically in variable co‐activity domains, were among the TFs that were most variably expressed and regulated the most variable genes (Fig EV3C).

The functional link between TFs and co‐activity variation is supported by a weaker correlation between TF expression and domain co‐activity scores in the absence of TFBSs within a domain for a given TF (*P* = 0.00068 based on all TFs and domains; Mann–Whitney *U*‐test, Fig EV3D). 10 of the considered TFs demonstrated a difference in PCC between bound and non‐bound regions (Welch Two‐Sample *t*‐test, BH‐adjusted *P* < 0.1; Fig EV3E), including the above‐named NFIC, NFATC1, and CEBPB. Finally, we observed specific enrichments of predicted TFBSs (Castro‐Mondragon *et al*, 2022) at gene promoters (−2 kb to +200 bp around annotated gene TSSs) in variable and matched non‐variable co‐activity domains compared to all co‐activity domains (Fig EV3F), indicating a different promoter grammar of variable co‐activity domains. In addition, the open chromatin regions in variable co‐activity domains shared more predicted TFBSs than those in matched non‐variable co‐activity domains (*P* = 1 × 10^−4^; Mann–Whitney *U*‐test, Fig 4C), indicating a more common grammar of regulatory elements in variable co‐activity domains. These findings together point to a strong association between variable co‐activity within a domain and the expression variability of regulating TFs between individuals, suggesting that TFs are key drivers of gene co‐activity.

**Figure 5:**
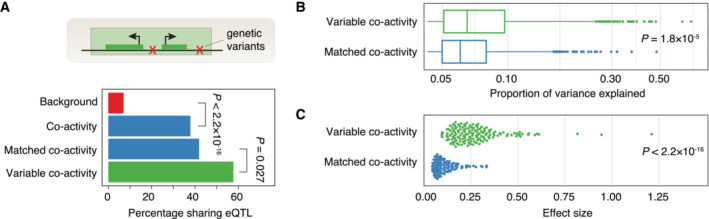
Genetic variation influences variability in co‐activity ATop: genotype variability could lead to variability in co‐activity level. Bottom: percentage of neighboring eGene pairs sharing an eQTL in variable and matched non‐variable co‐activity domains, as well as all non‐variable co‐activity domains and background regions (negative co‐activity scores). Differences in percentages were assessed using Fisher's exact test (*P*‐values shown).BProportion of co‐activity variance explained by co‐activity QTLs in variable and matched non‐variable domains. Mann–Whitney *U*‐test *P*‐value is shown. For box‐and‐whiskers, central band denotes the median, hinges the first and third quartiles, and the whiskers extend max 1.5 × IQR from the hinges.CCo‐activity score effect size of co‐activity QTLs in variable and matched non‐variable co‐activity domains. Mann–Whitney *U*‐test *P*‐value is shown.

Finally, we explored the association between variability in co‐activity and genotypic effects. Principal component analysis revealed a modest separation by ancestry of the individuals for the co‐activity scores, but less so for the positionally independent component or the raw expression data (Appendix Fig S2), suggesting that individual genetic variation may influence individual differences in the regulation of gene co‐activities. We found that 58% of neighboring gene pairs contained in variable co‐activity domains shared an eQTL (controlled for population stratification), compared to 42 and 38% in matched co‐activity domains and the full set of co‐activity domains, respectively (Fig 5A). In addition, when testing the association between SNPs and the average co‐activity score in a domain, we identified co‐activity QTLs for 68% (145 out of 212) of the variable co‐activity domains (Materials and Methods). In contrast, 51% (108 out of 212) of the matched non‐variable co‐activity domains were associated with co‐activity QTLs (Fisher's exact test, odds ratio 2.1, *P* = 0.0035). Thus, the association between genotype and co‐activity score was stronger but not unique to variable domains. We speculate that this is due to the fact that there is some inter‐individual variability also in matched domains, although to a lower degree (Fig EV2C). However, the total number of co‐activity QTLs associated with variable co‐activity domains was higher (1,323 compared to 876 for variable and matched non‐variable co‐activity domains, respectively), and co‐activity QTLs explained a larger fraction of co‐activity variation (*P* = 1.8 × 10^−5^; Mann–Whitney *U*‐test, Fig 5B) and were associated with larger effect sizes (*P* < 2.2 × 10^−16^; Mann–Whitney *U*‐test, Fig 5C) for variable co‐activity domains.

Taken together, through a systematic investigation of regulatory processes acting upon variable co‐activity domains, we conclude that all investigated regulatory inputs, that is, histone modifications, TF binding, enhancer‐promoter interactions, and genetic variants, associate with variability in co‐activity, suggesting that careful deconvolution is required to estimate their individual regulatory effect sizes influencing co‐activity.

### Transcription factor expression is the dominant regulatory determinant of co‐activity

To estimate the relative effects of different regulatory mechanisms influencing co‐activity, we employed multiple linear regression and calculated the individual contributions of predicted enhancer‐promoter interactions, genetic variation, and TF activities to the co‐activity scores in each variable co‐activity domain (Materials and Methods). We generally excluded the influence of histone modifications on gene co‐activity, since histone PTMs can likely both influence and be influenced by transcription (Millán‐Zambrano *et al*, 2022).

**Figure 6:**
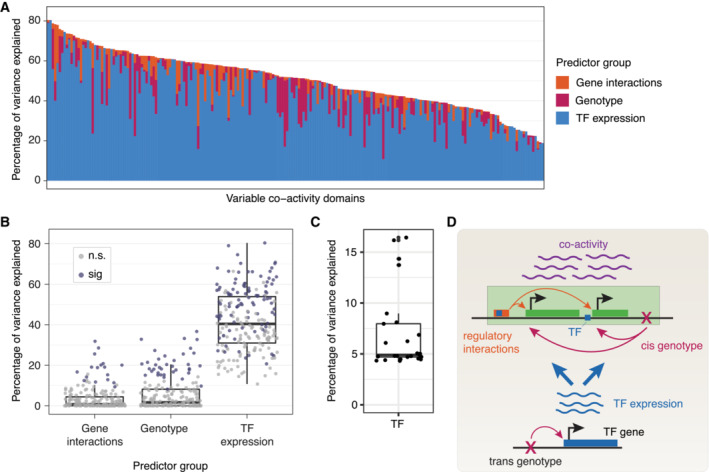
Transcription factor expression is the dominant regulatory determinant of co‐activity AThe proportion of variance in mean co‐activity explained by each predictor (gene interactions, genotype, and TF expression: stacked bars) in each variable co‐activity domain, with domains ordered by the total explained variance by all predictors together.BThe percentage of variance in mean co‐activity explained by each predictor, for variable co‐activity domains. Dots represent individual domains, colored according to whether excluding that predictor from the model containing all terms leads to a significant decrease in explained variation (ANOVA, *P* < 0.05). For box‐and‐whiskers, central band denotes the median, hinges the first and third quartiles, and the whiskers extend max 1.5 × IQR from the hinges.CPercentage of variance of TF expression explained by genotype of most significant eQTL. Box‐and‐whiskers defined as in panel B.DSchematic of proposed model of regulation of co‐activity. Co‐activity level (purple) is mainly influenced in *trans* by TF expression (blue), and to a lesser degree by gene interactions (orange) and local genotype (magenta) acting in *cis*. TF expression is itself influenced by local genetic variants (genotype) acting in *cis* and other unknown mechanisms. Arrow thicknesses provide a representation of the overall measured effect size of each mechanism.

**Figure EV4:**
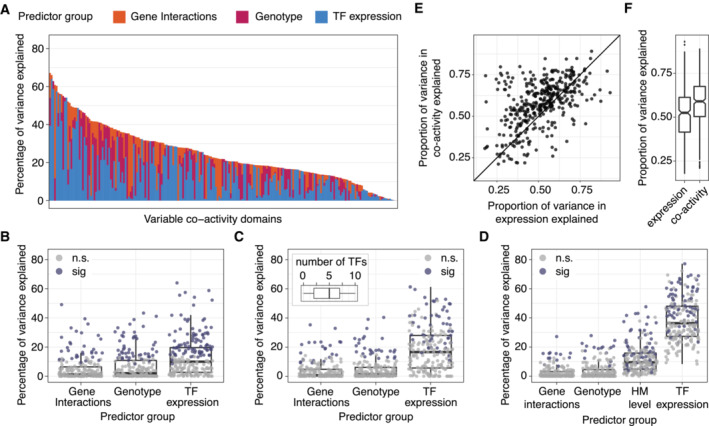
Comparison of different models AThe proportion of variance explained by each predictor (stacked bars) in each variable co‐activity domain, model including single top‐associating TF.BThe percentage of variance in mean co‐activity explained by each predictor, for variable co‐activity domains, in a model including single top‐associating TF. Dots represent variable‐co activity domains, colored by whether including the predictor leads to a significant decrease (ANOVA, *P* < 0.05) of the proportion of variance explained for this domain upon exclusion of the predictor in the model. For box‐and‐whiskers, central band denotes the median, hinges the first and third quartiles, and the whiskers extend max 1.5 × IQR from the hinges.CAs B, but for a model also adjusting for lab as a batch effect and limiting only to the set of transcription factors which have at least one predicted binding site within the modeled domain. Boxplot in top left indicates the distribution of the number of TFs included as variables in the model per domain (median 5 TFs). Box‐and‐whiskers defined as in panel B.DAs Fig 6B, for a model including levels of histone modifications H3K27ac, H3K4me1, and H3K4me3. Box‐and‐whiskers defined as in panel B.EScatter plot depicting the proportion of total variance in co‐activity explained by ENCODE TFs versus the proportion of total variance in log(expression) explained by the same set of TFs, based on a model including 343 individuals.FBoxplots depicting the relative distributions of the proportion of explained variance values for co‐activity and log(expression), as calculated in (E). Box‐and‐whiskers defined as in panel (B).

For each variable co‐activity domain, we used a selection procedure to find the 10 TFs that showed the highest combined importance in explaining the average co‐activity score in that domain (Materials and Methods). We further calculated, for each individual, a polygenic risk score‐inspired measurement (referred to as QTL summary score, QSS; Materials and Methods) combining the effects of the co‐activity QTLs associated with each domain with their individual alleles into a single variable. Then, for the 29 individuals with measured ABC interactions, we considered the following as predictors in an additive linear model: the total number interactions for genes in the domain, the QSS and the log of the expression levels for either each of the 10 most predictive TFs (Fig 6A and B) or the single most predictive TF (Fig EV4A and B) for that domain. For each model, we decomposed the sum of squares corresponding to the total variance into different parts, one for each considered regulatory input, and the residual sum of squares.

Overall, we observed considerable variation in the total amount of explained variance in domain co‐activity by the three mechanisms, both when considering 10 TFs in the model (Fig 6A, mean 51%, 95% confidence interval (CI): 49–53%) and when considering only the single most predictive TF (Fig EV4A, mean 25%, 95% CI: 23–27%). While the relative proportions of variance explained by the individual regulatory inputs also varied across the domains, in the majority of cases (95%, or 202 out of 212), the largest proportion of the explained variance was accounted for by the expression of TFs (Fig 6A; 58%, or 123 out of 212, when considering only a single TF, Fig EV4A). This demonstrates that TF expression variation is the dominant regulatory determinant of co‐activity. We observed similar results when we omitted the ABC interactions from the model, allowing modeling on the full set of 343 individuals (Appendix Fig S3A and B).

To strengthen our conclusions of the relative importance of each regulatory mechanism to co‐activity, we compared models by monitoring the change in R^2^ after omitting each predictor but retaining the others (ANOVA, Materials and Methods; Figs 6B and EV4B). We found that TF expression was a significant predictor in 96 out of the 212 analyzed domains (45% of domains, dropping to 42% for the single TF models), compared to 37 domains for which the QSS was a significant predictor (17%, raising to 23% for the single TF models). There were 17 domains (8%, increasing to 15% for the single TF models) for which the number of predicted enhancer‐promoter interactions was significantly associated with variability in co‐activity, with the increases in the single TF models suggesting that variation in the expression of a TF, local genotype and the enhancer‐promoter interactions do not behave fully orthogonally (Figs 6B and EV4B). In support, when modeling co‐activity scores using only ABC interactions as a single term, the percentage of variance explained by that predictor increased from 3% based on the full model to 5%, and the term was significant in 32 domains (15%) (Appendix Fig S3C). Furthermore, in order to test the robustness of these results, we repeated the analysis involving TFs, QSS and ABC interactions as predictors, now including as an extra covariate the laboratory each sample was derived from (Lappalainen *et al*, 2013), and also restricting the TFs considered to be only those which had at least one TFBS present in the relevant domain (median 5 TFs per domain, Fig EV4C). We also observed robust results when we included histone modifications (H3K27ac, H3K4me1, and H3K4me3) in the model (Fig EV4D). Furthermore, substituting the QSS with the genotype of the top co‐activity QTL yielded a slightly lower percentage of variance explained for the genotype term (Appendix Fig S3D and E), indicating that the inclusion of multiple QTLs into a single QSS adds explanatory power.

In order to see if the predictive power of TF expression was similar for both co‐activity and total expression within domains, we compared the proportion of total variance explained by TF expression across 343 individuals, individually for co‐activity and expression. This revealed that, while variability in TF expression could explain a sizable proportion of domain expression, it could, on average, explain a larger proportion of the variance in co‐activity (Fig EV4E and F). This suggests that TFs may preferentially exert co‐regulatory effects on multiple genes within variable domains, rather than act independently on single genes.

We speculated that the strong association between TF expression and co‐activity variability in variable co‐activity domains could be partly explained by eQTLs in *cis* of the TF genes themselves. Of 25 TFs identified as having both local eQTLs and being associated with co‐activity levels in at least one variable co‐activity domain, the lead SNP could explain 7% of TF expression variance on average (Fig 6C). Although our panel size does not yield sufficient power to map *trans* QTLs, this result and our modeling results above suggest that variability in co‐activity is driven by *trans* effects through genetic variants acting in *cis* on distal TF genes. Based on these results, we conclude that co‐activity is explained by a combination of both cis‐effects, including local sequence variation and enhancer‐promoter interactions, and trans‐effects resulting from variations in TF expression likely causing variation in TF binding to regulatory elements shared by genes in the domain (Fig 6D). In all, we identify TF expression as the strongest determinant of co‐activity.

## Discussion

In this study, we made use of the transcriptional decomposition approach (Rennie *et al*, 2018) to investigate regulatory mechanisms driving gene co‐activity across a wide panel of LCLs from 343 healthy individuals (Lappalainen *et al*, 2013). While originally developed to investigate domain co‐activity based on CAGE data across different cell types, we here slightly adapt the methodology (see Materials and Methods for details) and demonstrate its applicability to large‐scale RNA‐seq data from the same cell type. Transcriptional decomposition enabled the derivation of a co‐activity score that reflects the portion of expression attributable to positional contexts. Put in another way, we excluded the portion of expression that can be explained by a given gene independently of its neighboring genes. Measuring co‐activity in this way is useful for two reasons: firstly, it separates noise from the underlying signal representing shared regulatory effects within chromosomes, thereby allowing us to identify domains of co‐regulation, with a nuance importantly differing from approaches based on co‐expression between neighboring gene pairs (Kustatscher *et al*, 2017) or co‐variable histone PTM domains (Waszak *et al*, 2015; Delaneau *et al*, 2019). Secondly, using co‐activity scores allowed us to pin‐point regulatory mechanisms which may explain specific positional contexts. In other words, we asked if we could explain the necessity of groups of genes to be in genomic proximity in terms of the regulation of their activities.

Overall, co‐activity scores within defined co‐activity domains appeared stable across individuals and were reflective of local chromatin states, conforming to observations that topological and compartment domains have a tendency to remain consistent across cell types and individuals in a population (Rao *et al*, 2014; Gorkin *et al*, 2019). Furthermore, the co‐regulatory potential within a domain was manifested by shared eQTLs having an impact on multiple genes within the same domain, supported by observations of eGene pairs situated in close proximity within the genome (Strunz *et al*, 2021). We found regions within co‐activity domains that displayed significant variability in co‐activities to be particularly interesting, as their analysis allowed for a deeper understanding of the underlying mechanisms driving their variability.

When compared to their non‐variable counterparts, controlling for gene numbers and size, variable co‐activity domains were found to possess unique characteristics. These domains were associated with higher enrichments for binding sites of variably expressed TFs, more variability in gene regulatory interaction numbers across individuals, and a greater impact from local genotypes. The latter was manifested both through higher numbers of shared eQTLs and on average higher effect sizes of QTLs on co‐activity than those in non‐variable domains. This supports a model where high variability in domain‐scale activities is driven by high levels of variable regulatory inputs acting on a given locality (Andersson & Sandelin, 2020). These inputs are acting either in *cis* or in *trans* and potentially separated from additional mechanisms controlling independent regulation at individual genes, such as via binding to specific core promoter sequences. While it can be assumed that these types of inputs also drive co‐activity in non‐variable domains, we do note that variable domains could display intrinsic differences in their regulation. For instance, a strong correlation between TF abundance and transcriptional (co‐)activity of genes might mean a reduced complexity in the TF binding grammar at their promoters. Indeed, differences in variability have been associated with differences in core promoter architecture (Sigalova *et al*, 2020; Einarsson *et al*, 2022), in addition to differences in chromatin state (Faure *et al*, 2017) and regulatory inputs from distal enhancers (Sigalova *et al*, 2020).

In general, variable co‐activity domains showed strong overlap with co‐variable histone PTM domains (VCMs). VCMs were previously identified also in LCLs in a similar population of healthy individuals and, similar to our co‐activity domains, are also enriched in chromatin contacts and genetic variants (Waszak *et al*, 2015). Our work shows that co‐activity alone reflects properties captured by profiles of histone modifications from which VCMs are derived, albeit on a broader scale and approached from an alternative angle. While the relationship between histone PTM and expression domains is expected (Andersson & Sandelin, 2020), we speculate that changes in TF expression, which we find to be the main determinant of variability in co‐activity domains, could also drive variability in VCMs. This is supported by a model of coordinated activity of cis‐regulatory domains (CRDs) being driven by TF abundances (Delaneau *et al*, 2019).

In this study, we utilized the ABC model (Fulco *et al*, 2019) to predict enhancer‐gene interactions in 29 out of the 343 individuals in the panel. Our finding that only 62 genes showed significant correlation between numbers of associated predicted interactions per individual and gene expression levels is supported by the findings that very few loci with a variable chromatin state at enhancers can be linked to expression changes of nearby genes (Kasowski *et al*, 2013; Waszak *et al*, 2015). This could possibly reflect buffering activities of enhancers working together to achieve regulatory robustness within domains (Osterwalder *et al*, 2018). We note, however, that our result could also reflect low sensitivities in the called interactions, in part due to differences across individuals in the resolution of input data used for modeling, emphasizing the importance of future experiments to validate differences in enhancer‐gene connectivity between individuals and their impact on gene expression levels. However, the fact that 90% of these 62 genes had a correlation which was positive suggests that we are not simply capturing noise. In addition, studying naturally occurring variation in enhancer‐promoter interactions across individuals within the same cell type could limit our detection of perturbed enhancer‐promoter interactions that cause large changes in gene expression, as opposed to the expected impact of enhancer‐promoter re‐wiring across different cell types.

Our results reveal that, relative to local effects of eQTLs and ABC‐predicted interactions, TF abundance is the strongest driver of co‐activity variability. This concurs with previous observations that TF abundance influences coordinated variability (Delaneau *et al*, 2019), which is further supported by our observations that TF abundance was in general more predictive of co‐activity than expression. The strong association between TF expression variability and variability in domain co‐activity lead us to hypothesize that variable co‐activity domains, at least partly, reflect enhancer‐based gene regulatory networks (Kamal *et al*, 2023; preprint: González‐Blas *et al*, 2022), and that genotype variation in *cis* to TF genes drives trans‐effects on co‐activity in variable domains through altered binding to their regulatory elements. This hypothesis is supported by observations that, while the majority of genes are associated with local eQTLs (The GTEx Consortium, 2020), there is a large portion of variation that cannot directly be explained by local genetic variation (Liu *et al*, 2019), suggesting that distal variation may contribute to a sizable proportion of expression variation *in trans*. A recent model suggests that the majority of phenotypic effect sizes in complex traits can be explained by accumulated effects on peripheral genes acting on a core set of trait‐associated genes through gene regulatory networks (Boyle *et al*, 2017; Liu *et al*, 2019). Hence, distal genetic variants, e.g. those affecting TF genes, may have a larger accumulated *trans* effect on genes than their local counterparts.

Our results have important implications for future efforts to model transcriptional regulation and deciphering regulatory perturbations associated with disease, emphasizing the need to model altered TF expression alongside efforts to map regulatory domains and regulatory genetic variants associated with disease.

## Materials and Methods

### General analysis

Unless otherwise specified, all analysis was performed in R (R Core Team, 2020) using the tidyverse packages (Wickham *et al*, 2019). All annotations of genes were based on GENCODE 26 comprehensive gene annotations.

Due to potential mapping biases as a result of VDJ recombination, 10 kb bins that contained gene segments belonging to the immunoglobulin heavy, kappa or lambda genes (on chromosomes 14, 2 and 22 respectively) were excluded from all analyses.

### Processing GEUVADIS datasets

For transcriptional decomposition, GEUVADIS (Lappalainen *et al*, 2013) RNA‐seq libraries were downloaded from ENA (accession ERP001942), trimmed, and mapped using HISAT2 (Kim *et al*, 2019). Reads were aggregated in 10 kb bins using deepTools (Ramírez *et al*, 2016) bamcoverage. Libraries with a number of empty bins more than two standard deviations away from the mean were excluded from all analyses (see Dataset EV1 for included libraries).

For gene‐based analyses, the R package recount3 (Wilks *et al*, 2021) was used to obtain gene‐level quantifications (accession ERP001942).

### Transcriptional decomposition

The transcriptional decomposition model was fit to the 10 kb binned RNA‐seq datasets using a previously described approach (Rennie *et al*, 2018), which is based on a Bayesian hierarchical model that relies on the integrated nested Laplace approximation, implemented in the package R‐INLA (Rue *et al*, 2009). Briefly, INLA focuses on models which can be expressed as a conditional Markov random field (a widely used family of models which are particularly suited to modeling the underlying structural dependencies in data), and assumes that the parameter marginal distributions can be approximated using the integrated nested Laplace approximation, a step which greatly eases computational processing time and thus suited to large datasets.

Briefly, the transcriptional decomposition approach is as follows: let $y_{i}\ldots y_{N}$ relate to a chromosomal segment such that $y_{i}$ represents the total read count in bin i, for a total of N bins in the segment (which could in theory span the whole chromosome, but for modeling purposes we terminate the segments prior to large regions of non‐expressed consecutive bins—see below). We assume that these read counts are distributed as negative binomial, such that $y_{i}∼\mathrm{NB}\left(s^{-1}\;\mu_{i},\theta \right)$ where s is the library depth offset (number of millions of reads), $\mu_{i}$ is the mean RPM for bin i and θ is a hyperparameter representing the overdispersion. We model the log of the mean expression in a given bin i as a combination of two latent components and an intercept, in other words $\log\left(\mu_{i}\right)=\log\left(s\right)+\alpha +PD_{i}+PI_{i}$, where α is the intercept and $PD_{i}$ and $PI_{i}$ are the positionally dependent (co‐activity) and independent components respectively.

The co‐activity component was modeled as a first‐order random walk, dependent on neighboring bins and assuming normally distributed differences. This equates to the difference between neighboring bins, $PD_{i}-PD_{i-1}$ having a normal distribution with mean zero and variance $1/\tau_{\mathrm{PD}}$, and where for identifiability purposes the component is scaled to $\sum_{i=1}^{N}\left(PD_{i}-PD_{i-1}\right)=0.$

The positionally independent component was modeled assuming bins to be independent and identically distributed (IID) ($PI_{i}⊥PI_{i-1}$) where $PI_{i}$ is Gaussian with variance $1/\tau_{\mathrm{PI}}$. The model was fit as a hierarchical model in INLA, whereby priors for the hyperparameter θ was given a Gaussian prior and $\tau_{\mathrm{PD}}$ and $\tau_{\mathrm{PI}}$ were fixed according to the scheme described below. For each of the co‐activity and independent components, the mean and variance based on samples from modeled posterior was taken for each bin and used in subsequent analyses.

In order to facilitate fitting the model, which has a high memory demand, the libraries of expression data of individuals were randomly assigned to groups of between 40 and 50 individuals. To focus modeling efforts on transcribed genomic regions, the two largest consecutive regions with no mapped RNA‐seq data were removed from each chromosome. The remaining bins were divided into parts with a maximum length of 7,500 bins, optimizing for containing as many contiguous bases while being close to 7,500 bins in length. The model was run separately on each resulting chromosome part for each of the groups of individuals. Afterwards, the correlation between the different individual groups was assessed for each chromosome part, to ensure the models were comparable between individuals. Finally, the modeled chromosome parts were combined per individual for further analysis.

In order to achieve good convergence and maximum comparability across individuals, chromosomes, and groups, the hyperparameters of the model were fixed using the following strategy: A series of random walk and IID precision hyperparameters was used to run the model, and the combination most closely matching the CAGE‐derived transcriptional components of GM12878 (Rennie *et al*, 2018), in terms of component range and level of detail, and showing the same ratio expression captured by both components, was selected (precision $\tau_{\mathrm{PD}}$ for the random walk −5, precision $\tau_{PI}$ for the IID −1). While efforts were made to make the results as generalizable as possible, we cannot rule out small batch differences impacting our results. Furthermore, we did not investigate the impact of different resolutions (bin sizes) and/or parameterizations of the model itself on the overall results. These aspects could potentially be addressed in future studies.

Co‐activity scores for included individuals can be accessed at Zenodo (https://doi.org/10.5281/zenodo.7180322; Data ref: van Duin *et al*, 2022).

### Identification of co‐activity domains

Regions for which at least 15% of the individuals had a positive co‐activity score for at least 10 consecutive bins (100 kb) were identified. These regions were merged if the gap between them was 100 kb or less. Finally, regions containing at least two genes with a minimum expression of 0.1 TPM were considered for further analyses (Dataset EV2).

### Compartment analysis

Compartment locations were obtained (Rao *et al*, 2014) and lifted over to GRCh38 using the R package rtracklayer (Lawrence *et al*, 2009) with function liftOver. Compartments A1 and A2 were merged and denoted as active, B1 and B4 as facultative heterochromatin, and B2 and B3 as constitutive heterochromatin compartments. Compartment locations were overlapped with co‐activity domains and background regions, and the proportion of the total genomic size of co‐activity domains and background regions covered by different compartments was calculated.

### ATAC‐seq peak identification

A list of ATAC‐seq peak regions was created from Yoruban population ATAC‐seq data (Tehranchi *et al*, 2019), using the ENCODE ATAC‐seq pipeline (https://github.com/ENCODE‐DCC/atac‐seq‐pipeline). Peak regions were defined as ± 300 base pairs from the peak summit. In the case of overlapping peak regions (when two summits are closer than 300 bp), only the region with most CAGE‐derived (Einarsson *et al*, 2022) transcription initiation was kept.

### Histone PTM ChIP‐seq data analysis

All analyses involving H3K27ac, H3K4me1, and H3K4me3 histone PTMs were performed using re‐analyzed (Gorkin *et al*, 2019) ChIP‐seq data (Grubert *et al*, 2015), that were lifted over to GRCh38, and binned in 10 kb bins.

### TAD boundary enrichment analysis

Locations of Hi‐C boundaries for GM12878 in GRCh38 were downloaded from 4D nucleome (Dekker *et al*, 2017; accession 4DNFIVK5JOFU, original data (Rao *et al*, 2014)).

All 10 kb bins included in the transcriptional decomposition modeling were scored based on whether they contained a co‐activity domain boundary and/or a Hi‐C boundary. From this, a contingency table was constructed, upon which a Fisher's exact test was performed.

### eQTL analysis

eQTL analysis was performed using MatrixEQTL (Shabalin, 2012). Only non‐missing SNPs with a minor allele frequency of > 0.1 were included. The first three genotype principal components and the first 15 RNA‐seq principle components were used as covariates.

For analyses considering the number of eQTLs shared between neighboring gene pairs, only genes with at least one detected eQTL (eGenes) were considered. For analysis of eQTL sharing of neighboring eGene pairs over distance (Fig 2G), all neighboring eGene pairs were divided by their distance into bins containing an equal number of eGene pairs. Significance scores were obtained by comparing the number of eGene pairs with and without a common eQTL in co‐activity domains and background regions using Fisher's exact test.

Proportions of variance explained were calculated from MatrixEQTL results as follows: R2 = (t_statistic / sqrt(degrees_of_freedom + t_statistic^2))^2 where t_statistic is the *t* statistic for each SNP‐gene linear model and degrees_of_freedom denotes the number of degrees of freedom for the full model estimated by MatrixEQTL.

### ABC interaction predictions

The ABC model was run as recommended (https://github.com/broadinstitute/ABC‐Enhancer‐Gene‐Prediction) across 68 individuals using DNAse‐seq and H3K27ac ChIP‐seq bigwigs mapped to GRCh37 (Gorkin *et al*, 2019). Putative enhancer locations were defined using DNAse hypersensitive sites derived from one individual (GM19204), to ensure identical enhancer locations for all considered individuals. Hi‐C data for GM12878 (Rao *et al*, 2014) was used for contact frequency. While GM12878 is a LCL derived from an individual not included in the GEUVADIS set of individuals, differences in ABC scores across individuals are mostly driven by differences in activity and accessibility, justifying the use of Hi‐C from a separate individual (Fulco *et al*, 2019).

ABC scores for included individuals can be accessed at Zenodo (https://doi.org/10.5281/zenodo.7180322; Data ref: van Duin *et al*, 2022).

### Identification of variable co‐activity domains

Variable genomic regions within co‐activity domains, whereby the standard deviation of co‐activity score across the set of individuals was above 0.6 for at least 10 consecutive bins, were identified. Domains were merged if they were gapped by 100 kb or less. Finally, all regions containing less than two genes with a minimum expression of 0.1 TPM were removed (Dataset EV3).

To minimize potential biases due to observed differences in co‐activity scores, numbers of genes and domain length when comparing variable co‐activity domains to non‐variable co‐activity domains, we created a matched set of non‐variable co‐activity domains. We first filtered co‐activity domains whose genomic size overlapped at least 10% with variable co‐activity domains and then sampled a subset of non‐variable co‐activity domains that closely matched the above parameters, such that the dominating difference between the matched co‐activity domains and the variable co‐activity domains was the variability between individuals (Dataset EV4).

### Hi‐C contact frequency analysis

We obtained Hi‐C contact frequencies for 13 YRI individuals from the 4DNucleome project (accessions 4DNFIG 5O1OQS, 4DNFIH3OTR14, 4DNFIUATRW3Z, 4DNFIF9BDCNI, 4DNFIQD2DP2F, 4DNFINHT8P7C, 4DNFIQS8853L, 4DNFIGF8EM7M, 4DNFIUPG2ZBJ, 4DNFICKMT1CY, 4DNFIVBYCYGS, 4DNFIE4WWHMF, 4DNFI6V7ZQAE; Gorkin *et al*, 2019). 50 kb resolution was extracted using StrawR (https://github.com/aidenlab/straw; Durand *et al*, 2016), and contact frequencies of bins overlapping annotated gene TSSs (GENCODE v26) in both variable and matched positive domains were summed across 1 Mb. The mean and variability of contact frequencies were calculated across individuals for each domain class.

### VCM enrichment analysis

VCM locations were obtained (Delaneau *et al*, 2019) and lifted over to GRCh38. Enrichment was calculated by performing Fisher's exact test on all 10 kb bins in co‐variability regions, scored for whether they were in a variable co‐activity region and whether they overlapped a VCM.

### TFBS analysis

Experimental TF ChIP‐seq narrowpeak files for 83 TFs were obtained from ENCODE (Dunham *et al*, 2012). For TFs with multiple experiments available, only the experiment with the highest amount of reads was kept. For individual accession numbers, see Dataset EV5. To create a list of putative target genes per TF, promoter areas (2 kb upstream and 200 bp downstream of annotated TSSs) of all genes were overlapped with TF ChIP‐seq peaks.

The binding of TFs in open chromatin regions (OCRs) in co‐activity domains was assessed using 83 ENCODE TF ChIP‐seq peaks. Contingency tables were constructed with counts reflecting the whether the OCR was in a variable co‐activity domain (versus a non‐variable co‐activity domain), combined with whether or not it had at least one binding site for the TF in question. Using these contingency tables, Fisher's exact tests were performed. The same was repeated using matched non‐variable co‐activity domains instead of variable co‐activity domains, against the background of positive co‐activity domains.

### JASPAR TFBS analysis

JASPAR (Castro‐Mondragon *et al*, 2022) predicted TFBS locations were obtained in bigBed format, and subsequently imported into R. For all analyses, a score cutoff of 200 was used. This score is the normalized weight according to the range of weights that can possibly be obtained given the PWM for that TF. The weight of a JASPAR predicted binding site is the probability of observing the site given the PWM divided by the probability of observing the site by random chance.

#### JASPAR TFBS enrichment analysis

The JASPAR enrichment tool (Castro‐Mondragon *et al*, 2022) was used as described (https://bitbucket.org/CBGR/jaspar_enrichment/src/master/). For the domain‐wide analysis, variable and matched non‐variable co‐activity domains were separately supplied as foreground, with all co‐activity domains as background. For the promoter‐specific analysis, promoters of genes with an average log(TPM + 1) expression of more than 0.01 were extracted from variable and matched non‐variable co‐activity domains and supplied separately as foreground using all co‐activity domains as background.

#### Co‐activity QTL analysis

Co‐activity QTL analysis was performed similar to the eQTL analysis described above, separately for variable and matched non‐variable co‐activity regions. The mean co‐activity score per domain was used, and domains were treated as genes. The first three genotype PCs and the first 15 co‐activity PCs across variable/matched domains were included as covariates.

LD filtering was done by iteratively removing QTLs. First, all QTLs which had a genotype correlation with the top QTL (by significance) of > 0.8 were removed. Then, all QTLs which had a genotype correlation of > 0.8 with the QTL with the second lowest *P*‐value were removed, and so on, until all remaining QTLs had a genotype correlation of < 0.8 with all other QTLs.

### QTL summary score (QSS)

To summarize QTLs into a single score, all QTLs were first polarized with regards to the sign of their effect size, by inverting all genotypes for QTLs with a negative effect size, and taking the absolute effect size. Next, the effect sizes were used to weigh the contribution of risk alleles (now defined as leading to an increase in effect size). Thus, the mean of genotypes was computed, weighted by the effect size. A QSS closer to two indicates the individual has a combined cis genotype that is more likely to lead to a higher co‐activity score.

### Selecting the top 10 most predictive TFs per variable region

In order to robustly select transcription factors most closely associated with domain co‐activity variability, a Random Forest (Wright & Ziegler, 2017) model was first constructed for each co‐activity domain, using the mean co‐activity score per domain as response, and the TF expression levels for the 83 TFs that have ENCODE experimental binding data in GM12878 as predictors. The data was split into five training and five validation sets and models were run using five‐fold cross‐validation, requesting “impurity” importance scores. The importance scores for each TF were averaged over the five folds, and the 10 TFs with the highest average importance score were selected. A linear model was then constructed using each of these TFs as the explanatory variable, and the proportion of variance explained (*r*‐squared) was noted. The whole process was repeated 3 times, and the most frequently occurring 10 TFs over all top 10 TFs or the most frequently occuring top single TF (according to *R* ^2^) over all runs was used in the following modeling.

### Multiple linear model to investigate proportion of variance explained

For each variable co‐activity domain a multiple linear model (MLM) was constructed, using the following predictors over the set of 29 individuals with overlapping data availability: the total number of ABC connections, the QSS of cis co‐activity QTLs, and the expression values of top associated TFs (10 most predictive or single most predictive TF). The dependent variable was the average co‐activity score in the domain. Using analysis of variance (ANOVA), we calculated the proportions of the type II sum of squares attributed to each of the predictors, including the TFs as a single predictor group in the case where the top 10 was included. To make sure no overfitting was occurring due to the limited set of considered individuals, two additional models were constructed, involving as predictors the QSS and TF expression (top 10 or top single TF) for 343 individuals.

For further comparison and assessment of the robustness of the TF expression predictability, the laboratory from where each of the individual samples was handled (Lappalainen *et al*, 2013) was included as a covariate in each model, and the TF set considered was limited to those which had predicted TFBSs within the modeled domain (median 5 TFs). Comparison of the total variance explained by ENCODE TFs between the co‐activity and the log(expression) levels was carried out by modeling the TF expressions as predictors and either the co‐activity or log(expression) across the 343 individuals as response.

For further comparison and assessment of the robustness of the QSS measure, models were constructed using the genotype of the most significant co‐activity QTL per region, instead of the QSS. In order to calculate the significance of predictors on the given domain, adjusting for the other terms in the model, each predictor was held out one by one, and the linear model without that predictor was compared to the full model containing all of the predictions using ANOVA.

## Author contributions

**Lucas van Duin:** Conceptualization; software; formal analysis; investigation; visualization; methodology; writing – original draft; writing – review and editing. **Robert Krautz:** Methodology; writing – review and editing. **Sarah Rennie:** Conceptualization; software; formal analysis; supervision; methodology; writing – original draft; writing – review and editing. **Robin Andersson:** Conceptualization; supervision; funding acquisition; methodology; project administration; writing – review and editing.

## Disclosure and competing interests statement

The authors declare that they have no conflict of interest.

## Supporting information



Appendix S1

Expanded View Figures PDF

Dataset EV1

Dataset EV2

Dataset EV3

Dataset EV4

Dataset EV5

PDF+

## Data Availability

Co‐activity scores and ABC predicted interactions are available at Zenodo (https://doi.org/10.5281/zenodo.7180322; Data ref: van Duin *et al*, 2022). Code for core analyses of this manuscript is available at https://github.com/anderssonlab/van_Duin_et_al_2023.
