# Supplementary material for: Transcription factor expression is the main determinant of variability in gene co‐activity
Source: Mol Syst Biol. 2023 May 9;19(7):e11392. doi: 10.15252/msb.202211392 (PMC10333863; doi:10.15252/msb.202211392)
Supplement: Supplementary file 1 — Appendix S1 [file MSB-19-e11392-s002.pdf]

Appendix for

Transcription factor expression is the main determinant of variability in gene co-activity

Lucas van Duin[1], Robert Krautz[1], Sarah Rennie[1,*], Robin Andersson[1,*]

[1] Section for Computational and RNA Biology, Department of Biology, University of Copenhagen, 2200, Copenhagen, Denmark

* Correspondence should be addressed to S.R. (sarah.rennie@bio.ku.dk) and R.A. (robin@bio.ku.dk)

Contents

Appendix Figure S1 – page 2

Appendix Figure S2 – page 2

Appendix Figure S3 – page 3

Appendix Figures

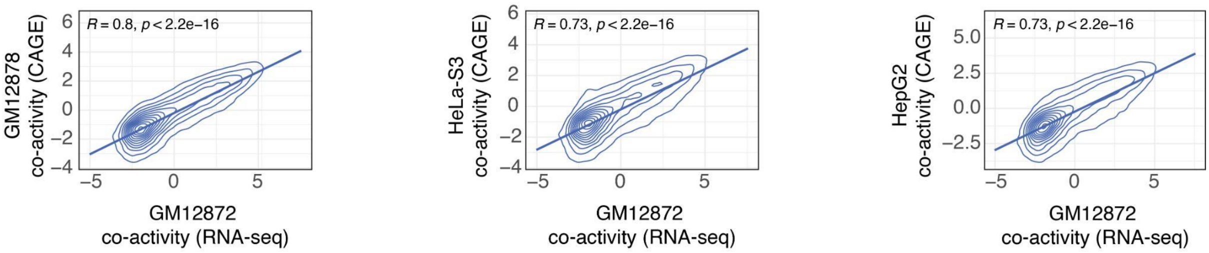

Appendix Figure S1. Comparison of co-activity scores between cell types. Comparison of RNA-seq derived co-activity scores of LCL GM12872 (horizontal axes) and CAGE-derived co-activity scores for GM12878, HeLa and HepG2 (vertical axes). PCCs (R) and p-values (correlation test) are provided.

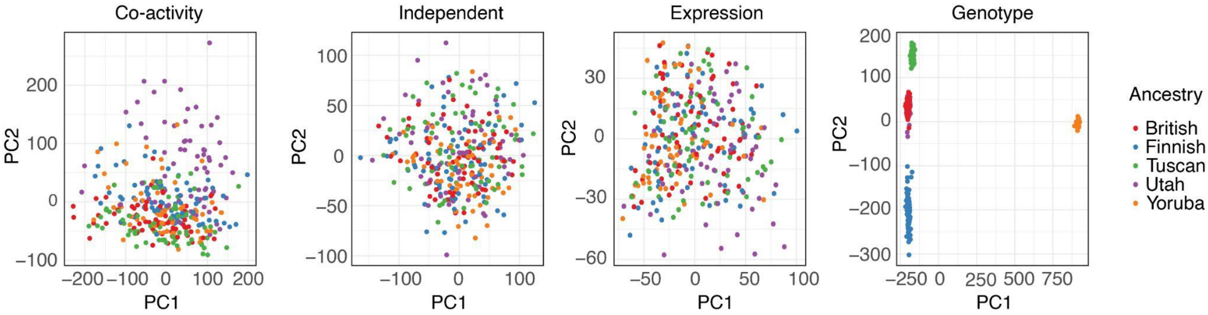

Appendix Figure S2. Evaluation of population stratification by first principal components. PCA plots of co-activity scores, positionally independent component, expression and genotype, colored by population.

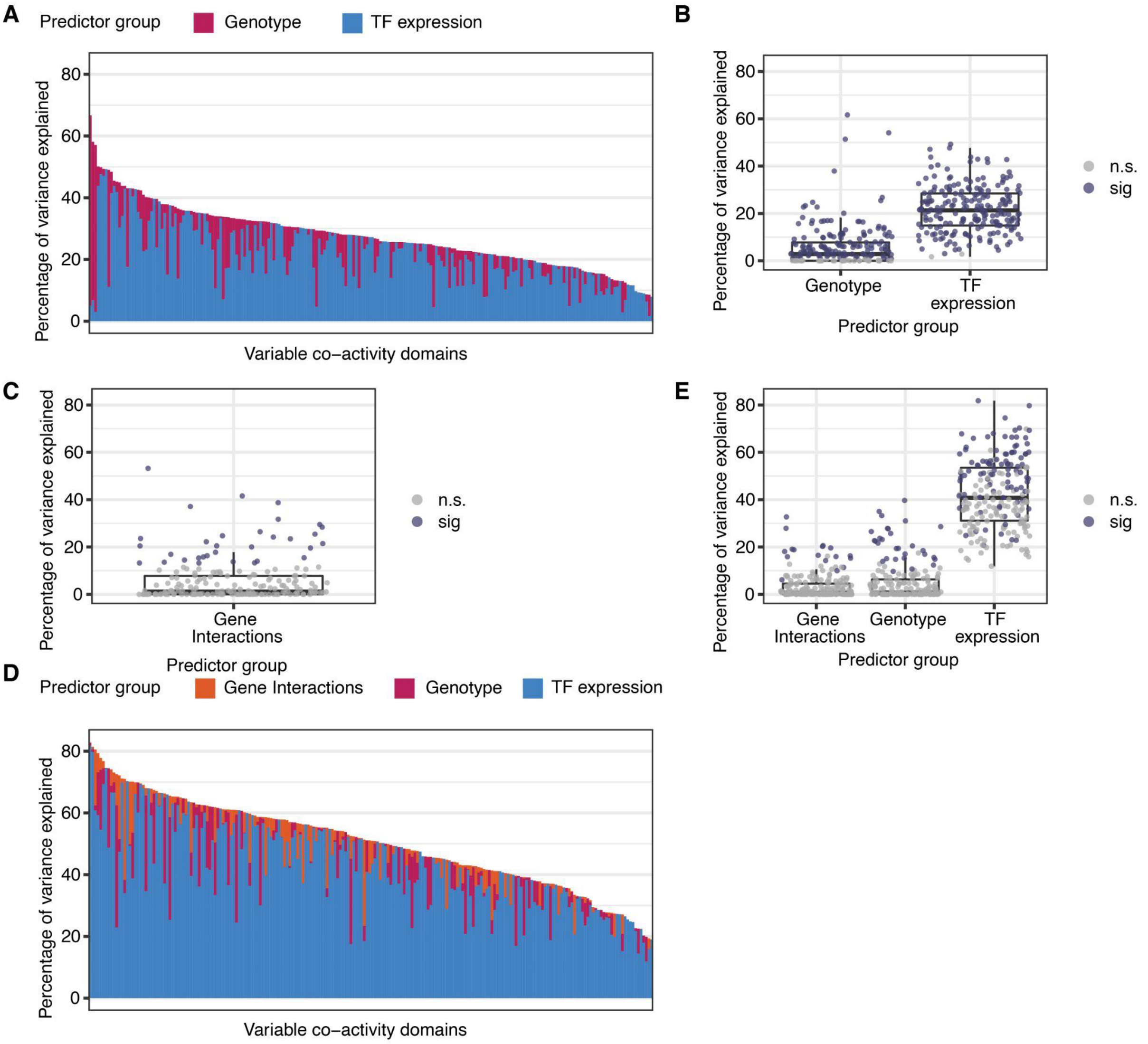

Appendix Figure S3. Extended comparison of different models. A: The proportion of variance explained by each predictor (stacked bars) in each variable co-activity domain, for a model excluding ABC-predicted interactions, expanding to 343 individuals. **B:** The percentage of variance in mean co-activity explained by each predictor, for variable co-activity domains, for a model excluding ABC-predicted interactions, expanding to 343 individuals. **C:** As B, for a model only including ABC-predicted interactions. **D:** As A, for a model including the genotype of the single most significant co-activity QTL per region, instead of QSS. **E:** As B, for a model including the genotype of the single most significant co-activity QTL per region, instead of QSS.
