# Supplementary material for: Transcription factor expression is the main determinant of variability in gene co‐activity
Source: Mol Syst Biol. 2023 May 9;19(7):e11392. doi: 10.15252/msb.202211392 (PMC10333863; doi:10.15252/msb.202211392)
Supplement: Supplementary file 2 — Expanded View Figures PDF [file MSB-19-e11392-s007.pdf]

Expanded View Figures

Figure EV1. Comparison of co-activity domains versus background regions.

A Co-expression (PCC) of neighboring gene pairs, stratified by the co-activity score of the upstream gene (horizontal axis) and the downstream gene (vertical axis) in each pair. Tiles are labeled by the percentage of correlated gene pairs (Pearson correlation test, BH-adjusted *P* value < 0.1).

B Histogram of the number of genes per co-activity domain.

C Histogram of co-activity domain sizes.

D The number of 10 kb genomic bins in co-activity domains, for different cutoffs based on the percentage of individuals showing a co-activity score above zero. The horizontal line indicates half of all bins in the genome.

E Proportion of expressed genes (TPM > 0.1, $n = 25{,}982$) and genome size in co-activity domains and background regions.

F Percentage of 10 kb bins showing significant (Pearson correlation test, BH-adjusted $P < 0.05$) correlation between co-activity score and histone PTM signal in co-activity domains and background regions.

G Relation between ABC-predicted interactions and expression, in terms of variability and level. Shown are Spearman's rho correlation values for (clockwise) the expression variability and the interaction variability, the expression variability and the number of interactions, the amount of expression and the number of interactions, and the amount of expression and the variability of interactions, per gene. Levels are median, variabilities Coefficient of Variation (CoV).

H Comparison of variability and number of ABC-predicted interactions per gene in co-activity domains and background regions. Mann–Whitney *U*-test *P*-values are shown. For box-and-whiskers, central band denotes the median, hinges the first and third quartiles, and the whiskers extend max $1.5 \times$ IQR from the hinges.

I PCC (horizontal axis) versus Pearson's correlation test *P*-value (vertical axis) for gene expression versus the number of ABC-derived gene interactions per individual. Color indicates an BH-adjusted *P*-value of < 0.1 (n.s., non-significant; sig, significant).

 © 2023 The Authors

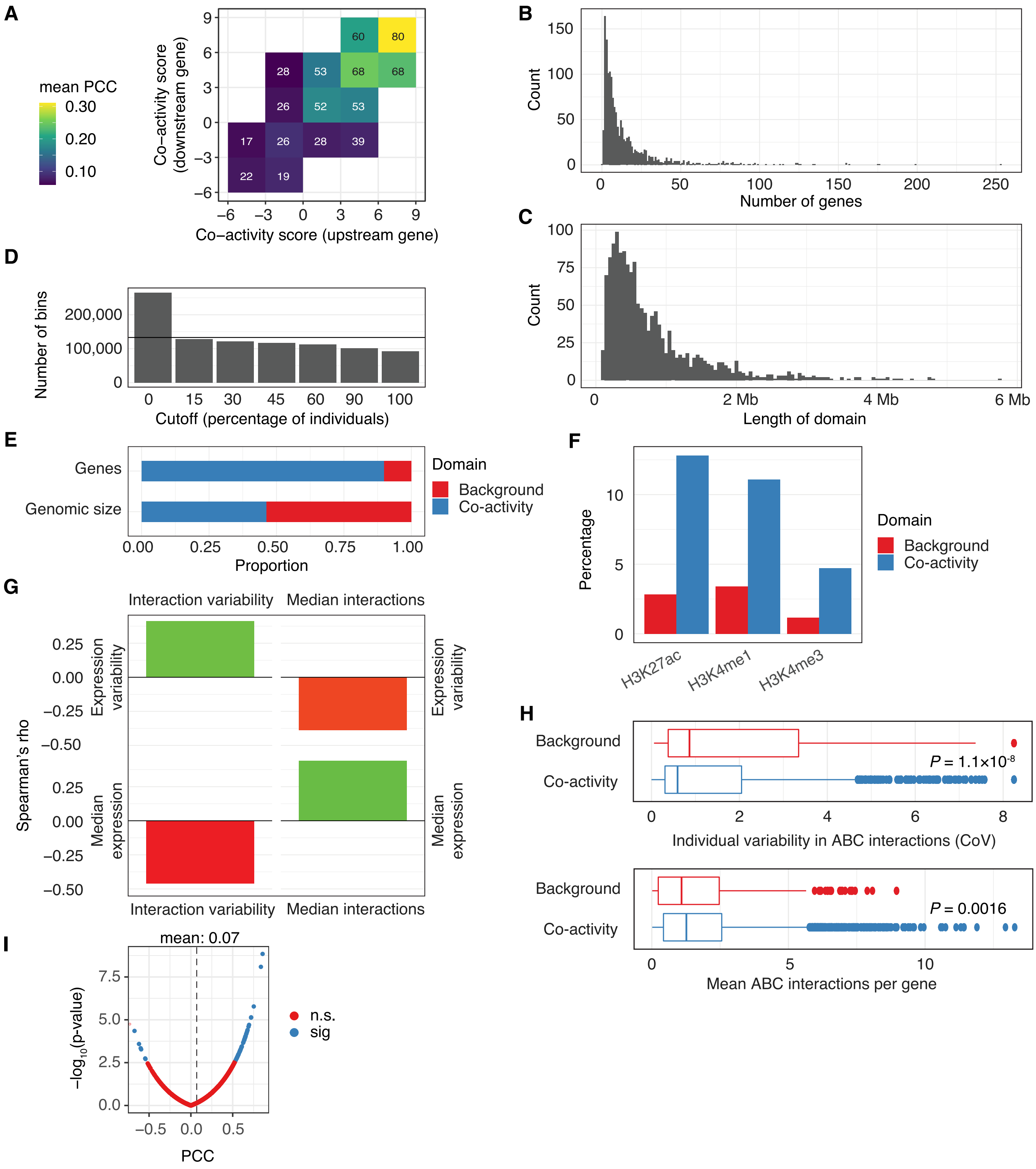
A
mean PCC
0.30
0.20
0.10
Co−activity score (downstream gene)
Co−activity score (upstream gene)
B
Count
Number of genes
C
Count
Length of domain
D
Number of bins
Cutoff (percentage of individuals)
E
Genes
Genomic size
Proportion
Domain
Background
Co-activity
F
Percentage
H3K27ac
H3K4me1
H3K4me3
G
Interaction variability
Median interactions
Spearman's rho
Expression variability
Median expression
H
Background
Co-activity
P = 1.1×10-8
Individual variability in ABC interactions (CoV)
P = 0.0016
Mean ABC interactions per gene
I
mean: 0.07
−log10(p-value)
PCC
n.s.
sig

Figure EV1.

◀ **Figure EV2. Comparison of variable co-activity domains versus non-variable co-activity domains.**

A Co-activity scores for all considered individuals in a region containing a variable co-activity domain (chr4:68,200,000-68,800,000).

B Comparison of co-activity score, number of contained genes and domain size of variable and all non-variable co-activity domains. For box-and-whiskers, central band denotes the median, hinges the first and third quartiles, and the whiskers extend max $1.5 \times$ IQR from the hinges.

C Comparison of co-activity score, variability, number of contained genes and domain size of variable and matched non-variable co-activity domains. Box-and-whiskers defined as in panel B.

D Number of ATAC-seq-inferred open chromatin regions (OCRs) per 10 kb in variable and matched non-variable co-activity domains. Mann–Whitney *U*-test *P*-value is shown. Box-and-whiskers defined as in panel (B).

E Variability (standard deviation) in Hi-C-derived interaction frequencies of 50 kb bins overlapping annotated gene TSSs summed across bins within 1 Mb in variable and matched non-variable co-activity domains. Mann–Whitney U-test *P*-value is shown. Box-and-whiskers defined as in panel (B).

F Average Hi-C-derived interaction frequencies of 50 kb bins overlapping annotated gene TSSs summed across bins within 1 Mb in variable and matched non-variable co-activity domains. Mann–Whitney *U*-test *P*-value is shown. Box-and-whiskers defined as in panel (B).

G Proportion of variable and matched non-variable co-activity domains showing a correlation (Pearson correlation test, BH-adjusted *P*-value < 0.1) between average co-activity score and average ChIP-seq histone PTM levels per domain.

H Density plot of VCM sizes (median: 52 kb, first vertical line; mean: 138 kb, second vertical line). 523 VCMs ($\sim$ 5%) surpassing the max considered size of 500 kb (VCM max width: 24 Mb) are excluded from the plot.

 © 2023 The Authors

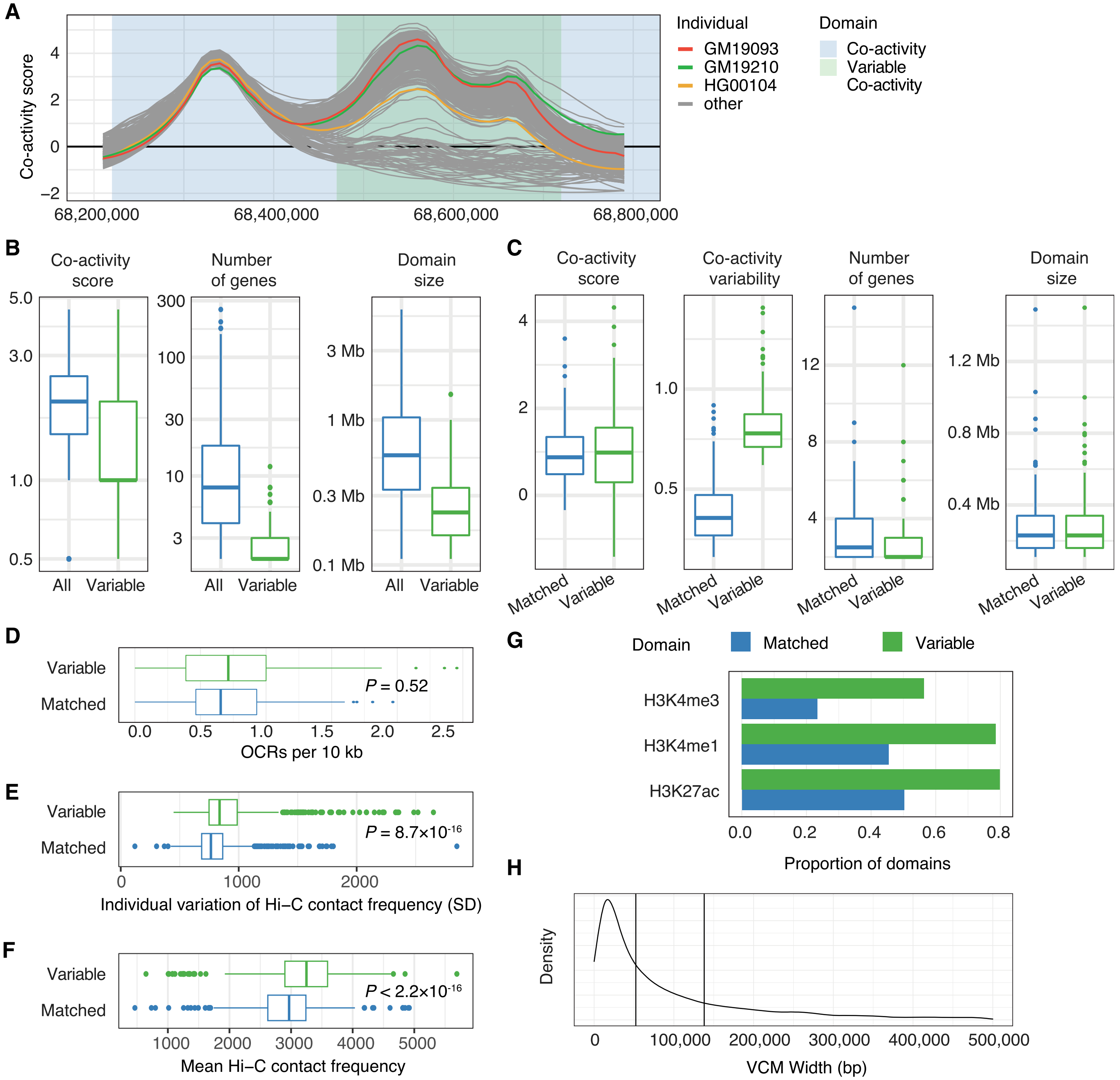
A
Co-activity score
4
2
0
−2
68,200,000
68,400,000
68,600,000
68,800,000
Individual
GM19093
GM19210
HG00104
other
Domain
Co-activity
Variable Co-activity
B
Co-activity score
Number of genes
Domain size
All Variable
C
Co-activity score
Co-activity variability
Number of genes
Domain size
Matched Variable
D
Variable
Matched
P = 0.52
OCRs per 10 kb
E
P = 8.7×10-16
Individual variation of Hi–C contact frequency (SD)
F
P < 2.2×10-16
Mean Hi–C contact frequency
G
Domain Matched Variable
H3K4me3
H3K4me1
H3K27ac
Proportion of domains
H
Density
VCM Width (bp)

Figure EV2.

◀ **Figure EV3. Transcription factor variability and binding differences versus co-activity variability.**

A Enrichment of predicted TFBSs in variable regions (odds ratio, horizontal axis) and expression variability (CoV, vertical axis) for each considered TF. PCC and Pearson correlation test *P*-value are shown.

B Enrichment of experimentally defined TFBSs (odds ratio, horizontal axis) in matched non-variable co-activity domains (all co-activity domains as background) versus TF expression variability (CoV, vertical axis). PCC and Pearson correlation test *P*-value are shown.

C TF expression variability (CoV, horizontal axis) versus variability of TF target genes (CoV, vertical axis). Each dot represents a TF, vertical axis value the mean CoV over all genes containing an ENCODE TFBS in their promoters. PCC and Pearson correlation test *P*-value are shown.

D Correlation (PCC) between TF expression and co-activity score for variable co-activity domains for which there are no identified TFBSs compared to variable co-activity domains with identified TFBSs, across all TFs and variable domains. Mann–Whitney *U*-test *P*-value is shown. For box-and-whiskers, central band denotes the median, hinges the first and third quartiles, and the whiskers extend max $1.5 \times$ IQR from the hinges.

E Correlation (PCC) between TF expression and co-activity score for variable co-activity domains with identified TFBSs compared to variable co-activity domains for which no TFBSs were identified, for 10 TFs showing differences in PCC (Welch Two-Sample *t*-test, BH-adjusted $P < 0.1$).

F Enrichment of predicted TFBSs in promoter regions (-2000 to +200 around annotated TSSs) of genes in variable and matched non-variable co-activity domains ($\log_2$ odds ratio, vertical axis) and associated significance (-$\log_{10}$(BH-adjusted *P*-value), horizontal axis).

 © 2023 The Authors

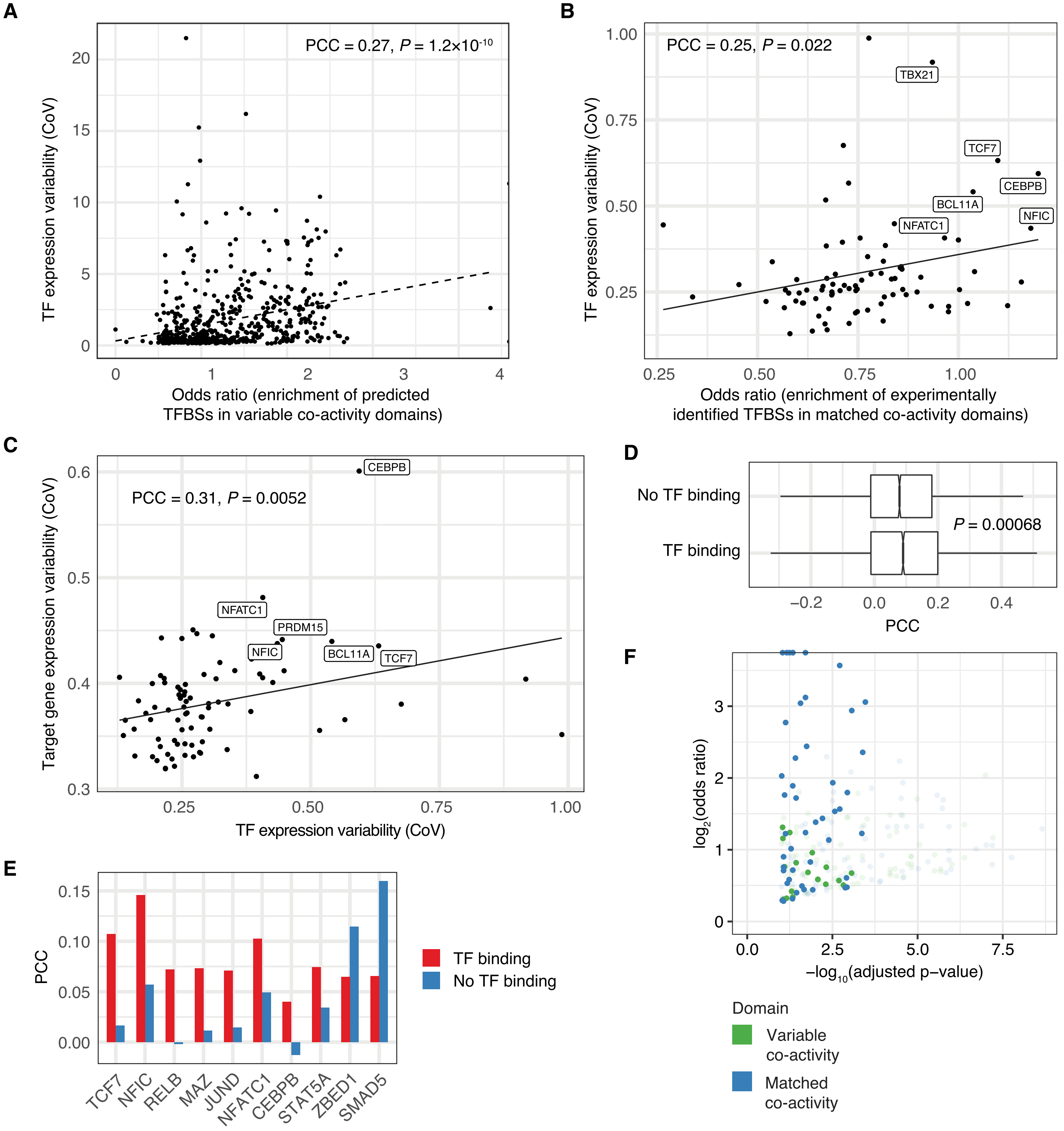
A
PCC = 0.27, P = 1.2×10-10
TF expression variability (CoV)
Odds ratio (enrichment of predicted TFBSs in variable co-activity domains)
B
PCC = 0.25, P = 0.022
TBX21
TCF7
CEBPB
BCL11A
NFATC1
NFIC
TF expression variability (CoV)
Odds ratio (enrichment of experimentally identified TFBSs in matched co-activity domains)
C
CEBPB
PCC = 0.31, P = 0.0052
NFATC1
PRDM15
NFIC
BCL11A
TCF7
Target gene expression variability (CoV)
TF expression variability (CoV)
D
No TF binding
TF binding
P = 0.00068
PCC
E
PCC
TF binding
No TF binding
TCF7
NFIC
RELB
MAZ
JUND
NFATC1
CEBPB
STAT5A
ZBED1
SMAD5
F
log2(odds ratio)
−log10(adjusted p−value)
Domain
Variable co-activity
Matched co-activity

Figure EV3.

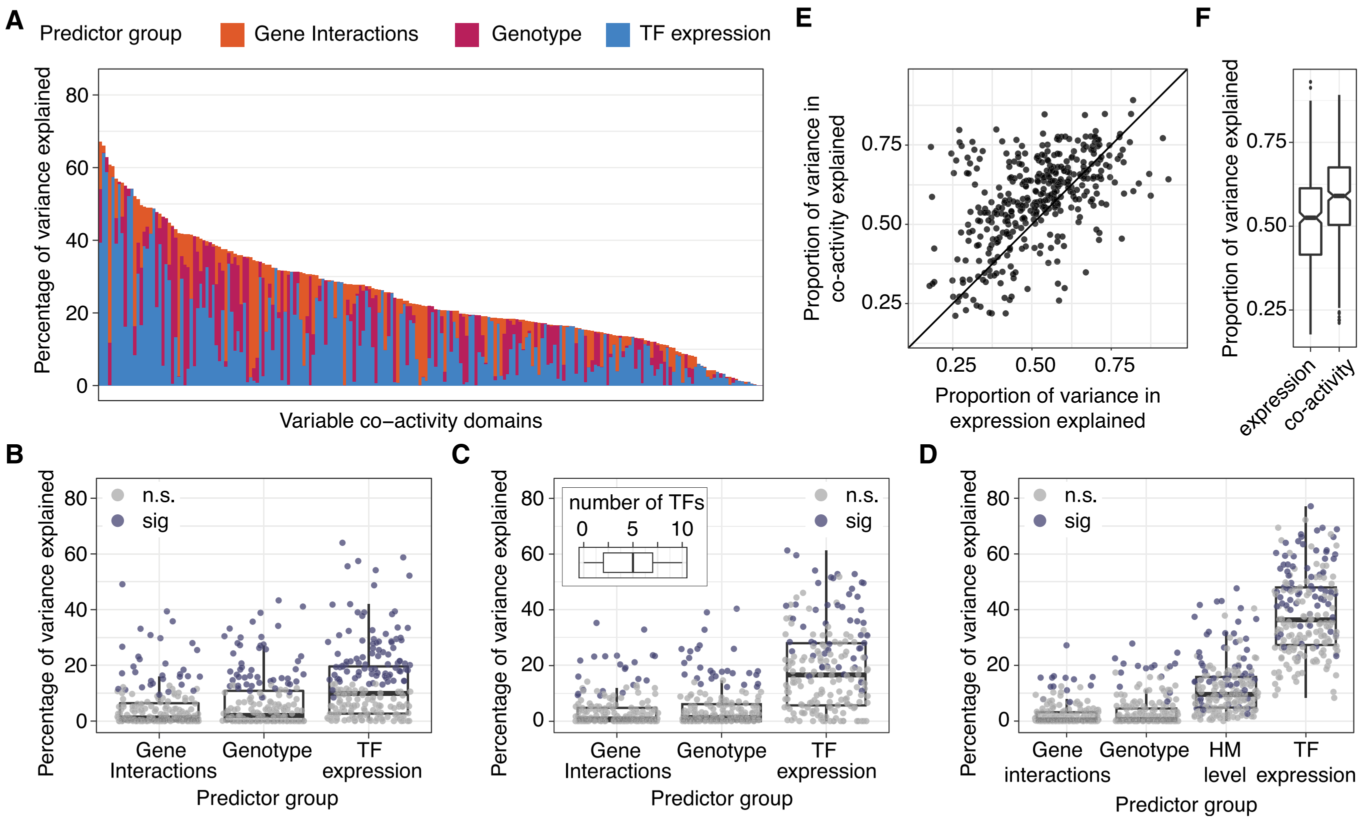

Figure EV4. Comparison of different models.

- A The proportion of variance explained by each predictor (stacked bars) in each variable co-activity domain, model including single top-associating TF.
- B The percentage of variance in mean co-activity explained by each predictor, for variable co-activity domains, in a model including single top-associating TF. Dots represent variable-co activity domains, colored by whether including the predictor leads to a significant decrease (ANOVA, $P < 0.05$) of the proportion of variance explained for this domain upon exclusion of the predictor in the model. For box-and-whiskers, central band denotes the median, hinges the first and third quartiles, and the whiskers extend max $1.5 \times$ IQR from the hinges.
- C As B, but for a model also adjusting for lab as a batch effect and limiting only to the set of transcription factors which have at least one predicted binding site within the modeled domain. Boxplot in top left indicates the distribution of the number of TFs included as variables in the model per domain (median 5 TFs). Box-and-whiskers defined as in panel B.
- D As Fig 6B, for a model including levels of histone modifications H3K27ac, H3K4me1, and H3K4me3. Box-and-whiskers defined as in panel B.
- E Scatter plot depicting the proportion of total variance in co-activity explained by ENCODE TFs versus the proportion of total variance in log(expression) explained by the same set of TFs, based on a model including 343 individuals.
- F Boxplots depicting the relative distributions of the proportion of explained variance values for co-activity and log(expression), as calculated in (E). Box-and-whiskers defined as in panel (B).

 © 2023 The Authors
