# Supplementary material for: Transcription factor expression is the main determinant of variability in gene co‐activity
Source: Mol Syst Biol. 2023 May 9;19(7):e11392. doi: 10.15252/msb.202211392 (PMC10333863; doi:10.15252/msb.202211392)
Supplement: Supplementary file 9 [file MSB-19-e11392-s008.pdf]

molecular systems biology

Transcription factor expression is the main determinant of variability in gene co-activity

Lucas van Duin, Robert Krautz, Sarah Rennie, and Robin Andersson
DOI: 10.15252/msb.202211392

Corresponding author(s): Robin Andersson (robin@bio.ku.dk) , Sarah Rennie (sarah.rennie@bio.ku.dk)

Review Timeline:

Submission Date:	13th Oct 22
Editorial Decision:	28th Nov 22
Revision Received:	24th Feb 23
Editorial Decision:	11th Apr 23
Revision Received:	13th Apr 23
Accepted:	17th Apr 23

Editor: Maria Polychronidou / Jingyi Hou

Transaction Report:

(Note: With the exception of the correction of typographical or spelling errors that could be a source of ambiguity, letters and reports are not edited. Depending on transfer agreements, referee reports obtained elsewhere may or may not be included in this compilation. Referee reports are anonymous unless the Referee chooses to sign their reports.)

28th Nov 2022

Manuscript Number: MSB-2022-11392
Title: Transcription factor expression is the main determinant of variability in gene co-activity
Author: Lucas van Duin
Robert Krautz
Sarah Rennie
Robin Andersson

Dear Dr Andersson,

Thank you for submitting your work to Molecular Systems Biology. We have now heard back from the three reviewers who agreed to evaluate your manuscript. As you will see from the reports below, the reviewers acknowledge the interest of the study. They raise however a series of concerns, which we would ask you to address in a major revision.

The recommendations of the reviewers are rather straightforward, so there is no need for me to reiterate the points listed below. In light of the comments of Reviewers #1 and #3, we would ask you to improve the clarity of the manuscript and make the methods and the concept of co-activity easily accessible to the general audience of Molecular Systems Biology.

All other issues raised by the reviewers need to be satisfactorily addressed as well. As you may already know, our editorial policy allows in principle a single round of major revision, and it is therefore essential to provide responses to the reviewers' comments that are as complete as possible. Please feel free to contact me in case you would like to discuss in further detail any of the issues raised by the reviewers.

On a more editorial level, we would ask you to address the following issues:

- Please provide a .docx formatted version of the manuscript text (including legends for main figures, EV figures and tables). Please make sure that the changes are highlighted to be clearly visible.

- Please provide individual production quality figure files as .eps, .tif, .jpg (one file per figure).

-Please provide a .docx formatted letter INCLUDING the reviewers' reports and your detailed point-by-point responses to their comments. As part of the EMBO Press transparent editorial process, the point-by-point response is part of the Review Process File (RPF), which will be published alongside your paper.

-Please note that all corresponding authors are required to supply an ORCID ID for their name upon submission of a revised manuscript.

-We replaced Supplementary Information with Expanded View (EV) Figures and Tables that are collapsible/expandable online (see examples in http://msb.embopress.org/content/11/6/812). A maximum of 5 EV Figures can be typeset. EV Figures should be cited as 'Figure EV1, Figure EV2" etc... in the text and their respective legends should be included in the main text after the legends of regular figures.

Additional Tables/Datasets should be labeled and referred to as Table EV1, Dataset EV1, etc. Legends have to be provided in a separate tab in case of .xls files. Alternatively, the legend can be supplied as a separate text file (README) and zipped together with the Table/Dataset file.

For the figures and tables that you do NOT wish to display as Expanded View figures, they should be bundled together with their legends in a single PDF file called *Appendix*, which should start with a short Table of Content. Each legend should be below the corresponding Figure/Table in the Appendix. Appendix figures and tables should be referred to in the main text as: "Appendix Figure S1, Appendix Figure S2, Appendix Table S1" etc. See detailed instructions regarding expanded view here: https://www.embopress.org/page/journal/17444292/authorguide#expandedview.

-Before submitting your revision, primary datasets (and computer code, where appropriate) produced in this study need to be deposited in an appropriate public database (seehttp://msb.embopress.org/authorguide - dataavailability https://www.embopress.org/page/journal/17444292/authorguide#dataavailability).

Please remember to provide a reviewer password if the datasets are not yet public.

The accession numbers and database should be listed in a formal "Data Availability" section (placed after Materials & Method) that follows the model below (see also https://www.embopress.org/page/journal/17444292/authorguide#dataavailability). Please

note that the Data Availability Section is restricted to new primary data that are part of this study.

Data availability

The datasets (and computer code) produced in this study are available in the following databases:

- RNA-Seq data: Gene Expression Omnibus GSE46843 (https://www.ncbi.nlm.nih.gov/geo/query/acc.cgi?acc=GSE46843)
- [data type]: [name of the resource] [accession number/identifier/doi] ([URL or identifiers.org/DATABASE:ACCESSION])

*** Note - All links should resolve to a page where the data can be accessed. ***

-At EMBO Press we ask authors to provide source data for the main figures. Our source data coordinator will contact you to discuss which figure panels we would need source data for and will also provide you with helpful tips on how to upload and organize the files.

- Our journal encourages inclusion of *data citations in the reference list* to directly cite datasets that were re-used and obtained from public databases. Data citations in the article text are distinct from normal bibliographical citations and should directly link to the database records from which the data can be accessed. In the main text, data citations are formatted as follows: "Data ref: Smith et al, 2001". In the Reference list, data citations must be labeled with "[DATASET]". A data reference must provide the database name, accession number/identifiers and a resolvable link to the landing page from which the data can be accessed at the end of the reference. Further instructions are available at .

- We updated our journal's competing interests policy in January 2022 and request authors to consider both actual and perceived competing interests. Please review the policy https://www.embopress.org/competing-interests and update your competing interests if necessary.

Please use the heading "Disclosure statement and competing interests".

- All Materials and Methods need to be described in the main text. We would encourage you to use 'Structured Methods', our new Materials and Methods format. According to this format, the Material and Methods section should include a Reagents and Tools Table (listing key reagents, experimental models, software and relevant equipment and including their sources and relevant identifiers) followed by a Methods and Protocols section in which we encourage the authors to describe their methods using a step-by-step protocol format with bullet points, to facilitate the adoption of the methodologies across labs. More information on how to adhere to this format as well as downloadable templates (.doc or .xls) for the Reagents and Tools Table can be found in our author guidelines: < https://www.embopress.org/page/journal/17444292/authorguide#researcharticleguide>. An example of a Method paper with Structured Methods can be found here: .

-Regarding data quantification:
Please ensure to specify the name of the statistical test used to generate error bars and P values, the number (n) of independent experiments (please specify technical or biological replicates) underlying each data point and the test used to calculate p-values in each figure legend. Discussion of statistical methodology can be reported in the materials and methods section, but figure legends should contain a basic description of n, P and the test applied.
Graphs must include a description of the bars and the error bars (s.d., s.e.m.).
Please also include scale bars in all microscopy images.

- Please provide a "standfirst text" summarizing the study in one or two sentences (approximately 250 characters, including space), three to four "bullet points" highlighting the main findings and a "synopsis image" (550px width and 400-600 px height, PNG format) to highlight the paper on our homepage.
Here are a couple of examples:
https://www.embopress.org/doi/10.15252/msb.20199356
https://www.embopress.org/doi/10.15252/msb.20209475
https://www.embopress.org/doi/10.15252/msb.209495

When you resubmit your manuscript, please download our CHECKLIST (https://www.embopress.org/pb-assets/embo-site/EMBO%20Press%20Author%20Checklist-1642513524327.xlsx) and include the completed form in your submission. *Please note* that the Author Checklist will be published alongside the paper as part of the transparent process (https://www.embopress.org/page/journal/17444292/authorguide#transparentprocess).

If you feel you can satisfactorily deal with these points and those listed by the referees, you may wish to submit a revised version of your manuscript. Please attach a covering letter giving details of the way in which you have handled each of the points raised by the referees. A revised manuscript will be once again subject to review and you probably understand that we can give you no guarantee at this stage that the eventual outcome will be favorable.

I look forward to receiving your revised manuscript soon.

Sincerely,
Jingyi

Jingyi Hou, PhD
Scientific Editor
Molecular Systems Biology

We realize that it is difficult to revise to a specific deadline. In the interest of protecting the conceptual advance provided by the work, we recommend a revision within 3 months (26th Feb 2023). Please discuss the revision progress ahead of this time with the editor if you require more time to complete the revisions. Use the link below to submit your revision:

https://msb.msubmit.net/cgi-bin/main.plex

IMPORTANT: When you send your revision, we will require the following items:
1. the manuscript text in LaTeX, RTF or MS Word format
2. a letter with a detailed description of the changes made in response to the referees. Please specify clearly the exact places in the text (pages and paragraphs) where each change has been made in response to each specific comment given
3. three to four 'bullet points' highlighting the main findings of your study
4. a short 'blurb' text summarizing in two sentences the study (max. 250 characters)
5. a 'thumbnail image' (550px width and max 400px height, Illustrator, PowerPoint or jpeg format), which can be used as 'visual title' for the synopsis section of your paper.
6. Please include an author contributions statement after the Acknowledgements section (see https://www.embopress.org/page/journal/17444292/authorguide)
7. Please complete the CHECKLIST available at (https://bit.ly/EMBOPressAuthorChecklist).
Please note that the Author Checklist will be published alongside the paper as part of the transparent process (https://www.embopress.org/page/journal/17444292/authorguide#transparentprocess).
8. When assembling figures, please refer to our figure preparation guideline in order to ensure proper formatting and readability in print as well as on screen:
https://bit.ly/EMBOPressFigurePreparationGuideline
See also figure legend guidelines: https://www.embopress.org/page/journal/17444292/authorguide#figureformat
9. Please note that corresponding authors are required to supply an ORCID ID for their name upon submission of a revised manuscript (EMBO Press signed a joint statement to encourage ORCID adoption).
(https://www.embopress.org/page/journal/17444292/authorguide#editorialprocess)
Currently, our records indicate that the ORCID for your account is 0000-0003-1516-879X.

Please click the link below to modify this ORCID:
Link Not Available

10. At EMBO Press we ask authors to provide source data for the main manuscript figures. Our source data coordinator will contact you to discuss which figure panels we would need source data for and will also provide you with helpful tips on how to upload and organize the files.

The system will prompt you to fill in your funding and payment information. This will allow Wiley to send you a quote for the article processing charge (APC) in case of acceptance. This quote takes into account any reduction or fee waivers that you may be eligible for. Authors do not need to pay any fees before their manuscript is accepted and transferred to the publisher.

EMBO Press participates in many Publish and Read agreements that allow authors to publish Open Access with reduced/no publication charges. Check your eligibility: https://authorservices.wiley.com/author-resources/Journal-Authors/open-access/affiliation-policies-payments/index.html

As a matter of course, please make sure that you have correctly followed the instructions for authors as given on the submission website.

*** PLEASE NOTE *** As part of the EMBO Press transparent editorial process initiative (see our Editorial at https://dx.doi.org/10.1038/msb.2010.72), Molecular Systems Biology publishes online a Review Process File with each accepted manuscripts. This file will be published in conjunction with your paper and will include the anonymous referee reports, your point-by-point response and all pertinent correspondence relating to the manuscript. If you do NOT want this File to be published, please inform the editorial office at msb@embo.org within 14 days upon receipt of the present letter.

Reviewer #1:

Van Duin et al applied their previously published transcriptional decomposition method to identify gene co-activity domains across LCL samples. While most co-activity domains were stable across individuals (as to be expected), a notable percentage varied in co-activity across individuals. The author's further analyzed these domains to determine factors that might be contributing to this variation in co-activity. By analyzing histone marks, predicted enhancer-gene interaction via ABC, and TF binding/expression and building a computational model to predict co-activity variation, the author's determined TF expression accounts for most of the observed variability.

Main points

The presentation of this manuscript could be greatly improved. It was very difficult to follow the narrative and it took re-reading paragraphs multiple times across multiple days to really understand what the authors were doing. I think the current presentation is a disservice to the work, which is actually quite interesting. For example, the author's assume the reader is familiar with the transcriptional decomposition method (paragraph 1, page 4). It would be very helpful to the reader to get a brief summary of the method and improve readability.

I am very hesitant about the heavy reliance on ABC predictions. ABC is highly dependent on H3K27ac and DNase signals as input. Therefore variability in these signals will result in variability in ABC calls. ABC predictions and histone marks should not be presented as separate lines of evidence (e.g. Figures 3D, E, F). Instead, if the author's wished to look at enhancer-gene interactions, it would be better to incorporate information about 3D chromatin loops (Hi-ChIP, ChIA-PET, etc). There is a lot of public data available for LCLs.

Minor points

What gene annotations are the author's using? The two genes highlighted in Figure 3 A & B (RP11XXX) are not in the current version of GENCODE. No gene version was provided in the methods.

Paragraph 3 on page 2 which talks about TADs seems out of place when paragraphs 2 & 4 end and start with discussions on co-expression, respectively.

Why were the top 10 TFs select via RF but then fed into a linear model? Some TFs have non-linear dependencies that would be picked up with RF model but then washed out with a linear model.

Reviewer #2:

In this manuscript, authors study the internal/external factors producing variations in co-activity of genes using LCL panel. The co-activity is defined as coordinated variability of two or more genes within a specific genomic neighborhood with or without co-expression. The authors can delineate the gene regulatory components that produce the co-activity but also differences in the expression of single genes using the transcriptional decomposition approach they developed (Rennie et al 2018). They find that co-activity patterns across individuals are stable on the domain levels (averaged out by multiple genes). However, they also find variations in co-activity across individuals in finer scales, driven by eQTLs potentially affecting TF affinities in the enhancers of individual genes. They also find that histone PTM variations correlated with that of co-activity. Importantly, they find that different TF levels across individuals explain more of the co-activity variation than that explained by the local genetic and chromatin variations.

This is a well-written manuscript. The authors tackle an important question in the field, namely, the reasons behind the co-regulation in relation to genomic neighborhood despite poor observed functional necessity for it. They identify a set of variable domains where the overall co-activity pattern is weaker and partially attribute this to TF level differences across individuals mediated by cis-eQTLs with larger effect sizes, providing a tool to prioritize variants contributing to inter-individual variability by use of co-activity domains. The finding the TFs variability is the main contributor for the observed co-activity variation is novel and significant. The application of co-activity scores for capturing inter-individual variability is novel and the findings have broad relevance to the field. I recommend the manuscript for publication after revision.

Major comments

1. Regarding the prediction of PE contacts using ABC modelling, authors claim that "co-activated" genes tend to vary less in their number of enhancers (page 8). However, this could be a result of ABC method overpredicting PE contacts in more distinct/strong histone signatures in the co-activity domains compared to background regions. Authors should address this especially in light with the results of the same analysis in variable co-activity domain (page 12).

2. "Similarly, genes located in co-activity domains were on average associated with more enhancers than those in background regions (1.8 versus 1.6 connections per gene on average, respectively; P = 0.0016, Wilcoxon signed-rank test; Figure 2F)". Is this not expected or given since there are more histone data ABC model can work on in co-activity domains in comparison to background (Supp. Figure 2E)? This conclusion could be drawn if the ABC model were to work irrespective of the genomic distance spanned by the PE contact. But ABC model is still skewed (albeit less than the nearest gene model) to shorter interaction distances.
3. Page 9, authors state "the expression of 62 genes showed significant correlation with the number of interactions (Pearson correlation test; BH-adjusted P < 0.1), for which the correlation sign was positive in 90% of cases. This suggests that the number of regulatory interactions reflects a gene's expression level within a co-activity domain, but that only few regulatory domains are sensitive to perturbations at an individual level." The enhancer number and gene expression levels might not correlate since they act upon different levels of transcription dynamics. The authors should clarify this point. Also what do authors mean by the last sentence is not clear.
4. Are the percentage differences stated in the last paragraph of page 14 statistically significant?
5. It is interesting that co-activity QTLs were associated with larger effect sizes (fig. 5c). This is most likely due to bettering of eQTL-target gene mapping using co-activity domains. The authors could highlight this point.
6. The co-activity scores seem to capture VCMs albeit at a lower resolution. Could this support that histone mark signatures are not the main driver/cause of VCM domains, but rather the result of a higher-order regulation/process? Authors should elaborate on this in relation with their conclusions.
7. The authors make the claim that variable co-activity domains could possess unique features that can explain their variability. However, this claim is not well supported. According to one line evidence given by the authors, then it should follow that promoters of genes in variable co-activity domains have differing regulatory grammar than those in stable co-activity domains. Have they tested this?
8. In the methods, the model fitting is done using groups of individuals to ease memory demand. Did the authors investigate how using groups of individuals rather than the full set of individuals (but on much smaller number of genomic bins per run (lower resolution)) affect the results?

Minor comments
1. Is supp. Fig 6c correct?
2. Is this sentence correct? "Put in another way, we excluded the portion of expression that can be explained by a given gene independently of its neighboring genes." (page 18) It is not clear to me what is it that is excluded.

Reviewer #3:

Based on a method published by the same authors earlier, this study quantifies the gene co-activity across individuals, characterises the variable co-activity domains and identifies TFs as main drivers for co-activity.

Overall, this is a nice study with solid descriptive analyses on co-activity domains. The key finding is that variation in gene co-activity is mostly driven by expression variation of TFs which bind to enhancers in these domains (based on motif enrichment). The variance explained by TF expression is quite impressive, however the significance of the findings in my view remain a bit hidden since the concept of co-activity is relatively hard to grasp and the authors could do a better job introducing it and relating it to known concepts, such as enhancer-based gene regulatory networks.

Major comments:

The concept of co-activity is hard to grasp. Since this study is based on a previously published method it will be important to again define with co-activity means, at least conceptually. It cannot be assumed that the reader knows the previous publication. Related, I find the schematic in Figure 1A not very clear. What are the purple and grey bars? Why are there no bars in the blue co-activity domain? What are the blue (TF?) squares reflecting?

P15 It would be important to assess also the importance of see chromatin marks, even if they are not necessarily direct.

In the final part where authors link TFs to co-regulated domains, it seems they are essentially reconstructing a enhancer-based gene regulatory network. If this interpretation is correct, it would be good to relate their findings to recent developments in enhancer-based gene regulatory networks (Glue, Granie, SCENIC+, Pando, to name a few).

The explanatory power of TFs seems very large (~60% variance explained by TFs), is that comparable to explaining expression levels (i.e. how much variance of expression variation would be explained by the same TFs?)? How does this compare to the level explained for genes are not in co-activity domains?

Have the authors tried how much variance of the co-activity domains is explained by the SNP that regulates the TF? (i.e. taking Figure 6C one step further)

Clarifications:

The description in the legend of Figure 1B is difficult to understand. This should be clarified in the methods section (and also mentioned in the main text)

Figure 1C: how many gene pairs are there? Why are some tiles empty? Can you add p-values to judge significance? Also, is this result expected just based on higher variance will lead to stronger correlation?

"co-activity domains were on average associated with more enhancers than those in background regions (1.8 versus 1.6 connections per gene on average, respectively; P = 0.0016, Wilcoxon signed-rank test; Figure 2F)": Figure 2F seems to be showing something else (eQTLs)

The authors should show some data for these statements: "On average, an individual's expression for a gene showed no or only weak correlation with its corresponding number of ABC-connections (PCC = 0.07). While this might reflect ABC measures being influenced by noise in the input data, the expression of 62 genes showed significant correlation with the number of interactions (Pearson correlation test; BH-adjusted P < 0.1), for which the correlation sign was positive in 90% of cases. This suggests that the number of regulatory interactions reflects a gene's expression level within a co-activity domain, but that only few regulatory domains are sensitive to perturbations at an individual level."

Claiming that Supplementary Figure 5 shows a clustering by genotype is an overstatement, at most the Utah population is partially separated.

Technical questions:

"Similarly, genes located in co-activity domains were on average associated with more enhancers than those in background regions": is this expected based on the analysis? i.e. both, co-activity and ABC-associated enhancers are based on correlation?

Page 12: Of 83 tested TFs (Supplementary Table 5) with experimentally identified binding sites in LCL. It would be great to see how this would perform when using predicted binding sites. This would be good to see, since it would determine whether the approach can be applied to cell types with less ChIP-seq data available.

Figure 2G: what is n in the legend? what is x axis?

Figure 3B: what are ABCDEF in the x- and y-axis?

minor:
page 4: "we modeled the positionally dependent portion of expression data independently in each LCL using aggregated expression in 10 kb tiled windows of the genome (Figure 1B)." How robust are the findings to changing the 10 kb window size?

We would like to thank the three reviewers for putting their valuable time and thought into reviewing our manuscript, and for the feedback that they have provided. We have carefully considered each comment - please see our responses below each relevant comment.

Reviewer #1:

Van Duin et al applied their previously published transcriptional decomposition method to identify gene co-activity domains across LCL samples. While most co-activity domains were stable across individuals (as to be expected), a notable percentage varied in co-activity across individuals. The author's further analyzed these domains to determine factors that might be contributing to this variation in co-activity. By analyzing histone marks, predicted enhancer-gene interaction via ABC, and TF binding/expression and building a computational model to predict co-activity variation, the author's determined TF expression accounts for most of the observed variability.

Main points

The presentation of this manuscript could be greatly improved. It was very difficult to follow the narrative and it took re-reading paragraphs multiple times across multiple days to really understand what the authors were doing. I think the current presentation is a disservice to the work, which is actually quite interesting. For example, the author's assume the reader is familiar with the transcriptional decomposition method (paragraph 1, page 4). It would be very helpful to the reader to get a brief summary of the method and improve readability.

> We thank the reviewer for their positive impression of our work. We agree that the method can be intuitively hard to follow, and in hindsight we should have included more description in order to minimize the requirement of the reader to refer back to the original text describing the method. Therefore, we have implemented the following changes to the text/figures with regards to the transcriptional decomposition method:
>
> - Expanded upon and clarified the explanation of the transcriptional decomposition in the main text. In particular, we have expanded upon paragraph 1 in the section “Transcriptional decomposition captures positionally dependent gene co-activities” to include further intuition, which should help the reader to understand conceptually what is being measured:
>   - “We have previously established transcriptional decomposition (Rennie *et al*, 2018), which is a novel Bayesian modeling-based approach for decomposing RNA expression in genomic bins along chromosomes into two parts: the portion of expression attributable to the local genomic neighborhood (positionally dependent (PD) component, referred to henceforth as ‘co-activity’ in the current study), and the portion of expression independent of the genomic position (positionally independent (PI) component). Formally, we model the log of the normalized (reads per million, RPM) expression of a given genomic bin as approximately PD + PI, where the value for PD (co-activity) is assumed to be dependent on neighboring bins, in contrast

to the value for PI, which is assumed to be independent of its neighbors. The co-activity portion is thus highly similar at close-by regions, which we hypothesize could result from a combination of shared regulatory mechanisms as well as similarities in the underlying chromatin environment, whilst the independent portion is uncorrelated with distance and likely reflects gene-specific regulatory mechanisms (Figure 1B) (Rennie *et al*, 2018)."

- Expanded upon the method in the main text:
  - **Added further mathematical details (see pasted text below)**
- Improved upon the overall clarity of Figure 1A, including the following specifics:
  - We have added an equation to more clearly depict that expression is split into two parts, and that one of them (co-activity) is the focus of the paper. We have further included the overall questions the study attempts to answer.
  - We have added small barcharts (purple) to better depict stable and variable co-activity domains - that is, according to how variable the mean co-activity in a domain is across the individuals.
  - On the right hand side we have replaced the blue squares that represented transcription factor (TF) abundance with a similar bar chart to depict variation in TF abundance across individuals.

Revised transcriptional decomposition methods section involving more comprehensive mathematical details:

"The transcriptional decomposition model was fit to the 10 kb binned RNA-seq datasets using a previously described approach (Rennie *et al*, 2018), which is based on a Bayesian hierarchical model that relies on the integrated nested laplace approximation, implemented in the package R-INLA (Rue *et al*, 2009). Briefly, INLA focuses on models which can be expressed as a conditional Markov random field (a widely used family of models which are particularly suited to modeling the underlying structural dependencies in data), and make the assumption that the parameter marginal distributions can be approximated using the integrated nested laplace approximation, a step which greatly eases computational processing time and thus suited to large datasets.

Briefly, the transcriptional decomposition approach is as follows: let $y_i \dots y_N$ relate to a chromosomal segment such that y_i represents the total read count in bin i, for a total of N bins in the segment (which could in theory span the whole chromosome, but for modeling purposes we terminate the segments prior to large regions of non-expressed consecutive bins - see below). We assume that these read counts are distributed as negative binomial, such that $y_i \sim NB(s^{-1}\mu_i, \theta)$ where s is the library depth offset (number of millions of reads), μ_i is the mean RPM for bin i and θ is a hyperparameter representing the overdispersion. We model the log of the mean expression in a given bin i as a combination of two latent components and an intercept, in other words $log\,(\mu_i) = log\,(s) + \alpha + PD_i + PI_i$, where α is the intercept

and PD_i and PI_i are the positionally dependent (co-activity) and independent components respectively.
The co-activity component was modeled as a first-order random walk, dependent on neighboring bins and assuming normally distributed differences. This equates to the difference between neighboring bins, $PD_i - PD_{i-1}$ having a normal distribution with mean zero and variance $1/\tau_{PD}$, and where for identifiability purposes the component is scaled to $\sum_{i=1}^{N}(PD_i - PD_{i-1}) = 0$.

The positionally independent component was modeled assuming bins to be independent and identically distributed (IID) $(PI_i \sqcup PI_{i-1})$ where PI_i is Guassian with variance $1/\tau_{PI}$. The model was fit as a hierarchical model in INLA, whereby priors for the hyperparameter θ was given a gaussian prior and τ_{PD} and τ_{PI} were fixed according to the scheme described below. For each of the co-activity and independent components, the mean and variance based on samples from modeled posterior was taken for each bin and used in subsequent analyses. "

I am very hesitant about the heavy reliance on ABC predictions. ABC is highly dependent on H3K27ac and DNase signals as input. Therefore variability in these signals will result in variability in ABC calls. ABC predictions and histone marks should not be presented as separate lines of evidence (e.g. Figures 3D, E, F). Instead, if the author's wished to look at enhancer-gene interactions, it would be better to incorporate information about 3D chromatin loops (Hi-ChIP, ChIA-PET, etc). There is a lot of public data available for LCLs.

We thank the reviewer for the comment on the use of ABC predictions in our manuscript. It is acknowledged that ABC is heavily dependent on H3K27ac and DNase signals as input, and therefore non-biological variability in these signals could be affecting the accuracy of our ABC calls (and thus the ability to reliably distinguish between different individuals). In our study, we calculated the ABC scores using the same GM12878 HiC dataset for all individuals (which is not unreasonable to do - see (Fulco *et al*, 2019), which shows that using an average HiC score for all cell types results in only a minor decrease in ABC performance). The incorporation of this dataset is valuable for driving the distance-decay relationship between the promoter and enhancer. Since it cannot account for individual-specific differences, it is the H3K27ac and DNase which are driving differences between individuals.

Whilst we appreciate that there is a lot of public LCL data available, to our knowledge the only available data source of genome-wide chromatin interaction data for different LCL individuals included in the present study is (Gorkin et al, 2019), which has low resolution HiC for 13 of the individuals considered in our study. To show that the differences in ABC scores are, at least partly, measuring contact differences, we have performed a similar analysis to that shown in Figure 3D, but based on HiC contact frequencies for 13 YRI individuals, obtained from the 4DNucleome project (accessions 4DNFIG5O1OQS, 4DNFIH3OTR14, 4DNFIUATRW3Z, 4DNFIF9BDCNI, 4DNFIQD2DP2F, 4DNFINHT8P7C, 4DNFIQS8853L, 4DNFIGF8EM7M, 4DNFIUPG2ZBJ, 4DNFICKMT1CY, 4DNFIVBYCYGS, 4DNFIE4WWHMF, 4DNFI6V7ZQAE) (Gorkin et al, 2019). In brief, we extracted 50 kb bins overlapping annotated gene TSSs in both variable and matched positive domains and summed all connections across bins within 1 Mb. We then compared the mean and variability

across the individuals for each of the two domain classes. Please see the figure below, which we have now included as a supplementary figure (Figure EV2E,F):

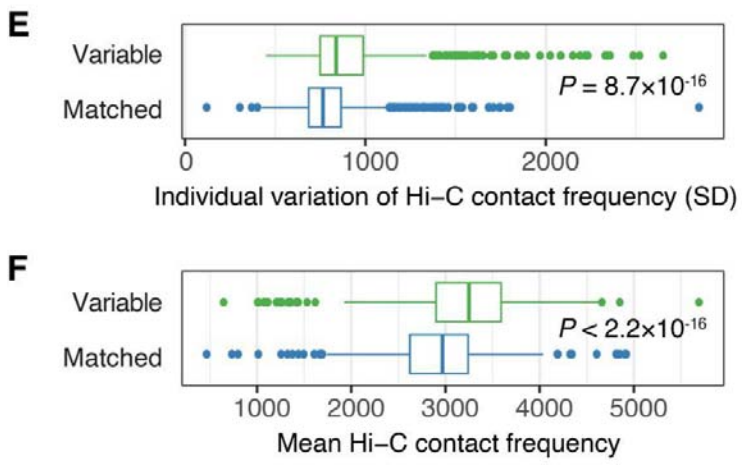

Finally, with regards to the usage of the ABC predictions and histone marks as separate lines of evidence, we would like to point out that the ABC method involves various processing and normalization steps, such as dividing the ABC scores by the number of putative contacts within a region. Furthermore, since it also includes information from HiC, it cannot solely be interpreted similarly to histone marks in isolation. Therefore, we hope that the reviewer can agree that including the histone marks within separate analyses is useful for the two following reasons:

- It would be informative beyond what is measured with ABC
- It would complement the analysis on the histone marks which were not included in the ABC score (such as H3K27me3 and H3K4me3), by offering direct comparisons.

Minor points

What gene annotations are the author's using? The two genes highlighted in Figure 3 A & B (RP11XXX) are not in the current version of GENCODE. No gene version was provided in the methods.

We apologize for the lack of clarity regarding the annotations used in the study. The annotations are based on GENCODE 26. They are available in file "gencode.v26.annotation.gtf" downloaded from https://www.gencodegenes.org/human/release_26.html, and the relevant information is here:

RP11-588H23.3: ENSG00000258168.5, chr 12 between 70468080 and 70543040
RP11-785D18.3: ENSG00000277247.1, chr 12 between 70570969 and 70571440

Both of these genes are lncRNAs that divergently overlap the 3' end of the PTPRB gene. In hindsight, it might have been better to select a locus with known mRNA

genes. Therefore we have redone Figures 3A & B and Figure EV2A using a new locus (see also below for convenience).

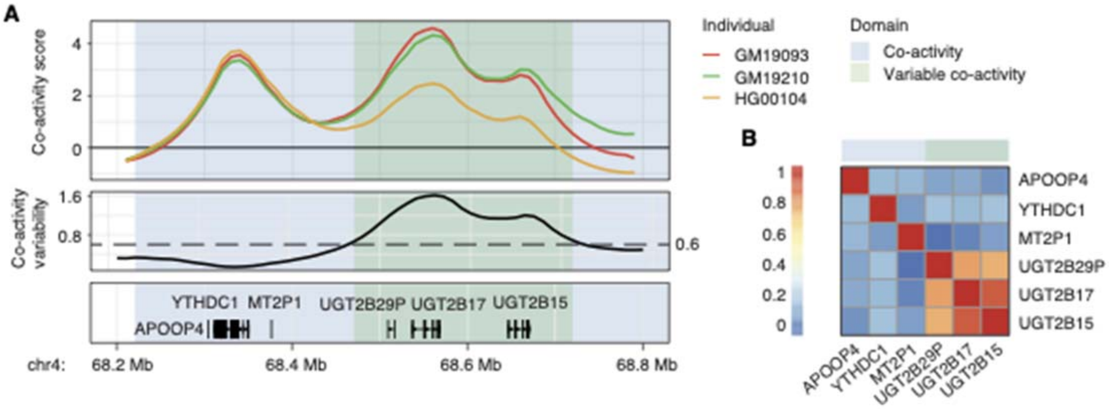

Paragraph 3 on page 2 which talks about TADs seems out of place when paragraphs 2 & 4 end and start with discussions on co-expression, respectively.

We thank the reviewer for pointing this out. We have now changed the order of the paragraph in the main text, so that the TAD overview is after the two paragraphs that discuss co-expression. We hope it is agreed that this improves the readability of the introduction.

Why were the top 10 TFs select via RF but then fed into a linear model? Some TFs have non-linear dependencies that would be picked up with RF model but then washed out with a linear model.

The reviewer correctly points out that the RF model captures non-linear dependencies. However, out of a possible 83 TFs, the search space for finding 10 TFs is enormous, so we needed to short-cut this search to find an approximate set likely to be the most important. Therefore, we used the RF model as a fast way to pre-select likely candidates, and then refined our search with linear models, to identify the best 10 from the set of candidates identified in the various test runs from the RF analysis. We would like to point out that these top 10 TFs would, if anything, more likely underrepresent the explained variation in co-activity, and therefore this process would not reflect on our overall conclusion of the paper (put in other words, a better set would likely only strengthen our conclusion that TF expression is the strongest determinant of co-activity).

Reviewer #2:

In this manuscript, authors study the internal/external factors producing variations in co-activity of genes using LCL panel. The co-activity is defined as coordinated variability of two or more genes within a specific genomic neighborhood with or without co-expression. The authors can delineate the gene regulatory components that produce the co-activity but also differences in the expression of single genes using the transcriptional decomposition approach they developed (Rennie et al 2018). They find that co-activity patterns across

individuals are stable on the domain levels (averaged out by multiple genes). However, they also find variations in co-activity across individuals in finer scales, driven by eQTLs potentially affecting TF affinities in the enhancers of individual genes. They also find that histone PTM variations correlated with that of co-activity. Importantly, they find that different TF levels across individuals explain more of the co-activity variation than that explained by the local genetic and chromatin variations.

This is a well-written manuscript. The authors tackle an important question in the field, namely, the reasons behind the co-regulation in relation to genomic neighborhood despite poor observed functional necessity for it. They identify a set of variable domains where the overall co-activity pattern is weaker and partially attribute this to TF level differences across individuals mediated by cis-eQTLs with larger effect sizes, providing a tool to prioritize variants contributing to inter-individual variability by use of co-activity domains. The finding the TFs variability is the main contributor for the observed co-activity variation is novel and significant. The application of co-activity scores for capturing inter-individual variability is novel and the findings have broad relevance to the field. I recommend the manuscript for publication after revision.

> We thank the reviewer for the praise of our manuscript, and address their comments below.

Major comments

1. Regarding the prediction of PE contacts using ABC modelling, authors claim that "co-activated" genes tend to vary less in their number of enhancers (page 8). However, this could be a result of ABC method overpredicting PE contacts in more distinct/strong histone signatures in the co-activity domains compared to background regions. Authors should address this especially in light with the results of the same analysis in variable co-activity domain (page 12).

> We thank the reviewer for their comment, and agree that the ABC method might be difficult to compare between regions of weak or strong chromatin signatures (as a proxy for activity). In regions with weak signals, contacts for an enhancer could be missed for some individuals, leading to the impression that contact variability is higher. In the case of the background regions, which have both a lower density of putative enhancers per gene and lower activity per putative enhancer, it may therefore be misleading to compare contact variability directly.
>
> In terms of comparison between variable co-activity and co-activity domains (Figure 3D), we addressed this issue by defining a set of “matched” co-activity domains. These have similar properties to variable co-activity domains in terms of their overall activity, number of genes and length, and differ only in terms of their co-activity variability (that is, the feature used to define them as variable co-activity domains). In Figure 3D, we see that variability in enhancer number is higher, and due to the above reason this is likely not explained by differences in the underlying expression / histone densities. Furthermore, this difference is more significant ($P = 8\times10^{-10}$; Mann-Whitney U test, Figure 3D) than the difference between co-activity and background domains, where the underlying landscape differs more strongly ($P = 1.1\times10^{-8}$; Mann-Whitney U test, Figure EV1H). Therefore, we conclude that our data is supportive of

genes in variable domains being more variable in their enhancer numbers compared to their co-active counterparts (all things being equal, although we cannot rule out other biases in our analysis).

We have attempted to clarify this in the main text by implementing the following changes:

- We have softened the claims of Figure EV1H.
- We have added the Mann-Whitney U test p-value to the main Figure 3D to the differences between the matched and variable co-activity domains for the ABC scores.
- We explicitly note in the main text referring to Figure 3D that the result is accounting for differences in underlying domain activity.
- We concede that there may still be biases due to both the ABC method (which are just predictions) and other possible differences between the matched and variable domains that we have not accounted for, and make it clear in the Discussion that follow-up experiments are important for validating interactions at individual genes and enhancers.

Further to this, and in response to comments by Reviewer 1, we have performed additional analysis to show that the ABC scores are, at least in part, measuring contact differences: we have carried out a similar analysis to that shown in Figure 3D, but based on HiC contact frequencies for 13 YRI individuals (Gorkin et al, 2019). In brief, we extracted 50 kb bins overlapping annotated gene TSSs in both variable and matched positive domains and summed all connections across bins within 1 Mb. We then compared the mean and variability across the individuals for each of the two domain classes. Please see the figure below, which we have now included in (Figure EV2E,F):

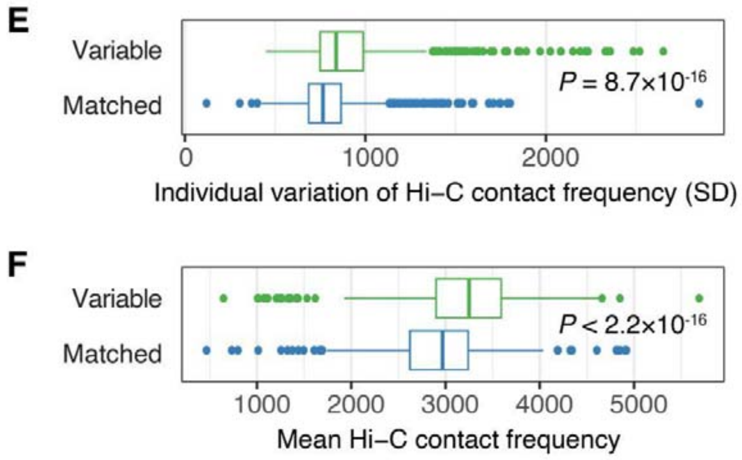

2. "Similarly, genes located in co-activity domains were on average associated with more enhancers than those in background regions (1.8 versus 1.6 connections per gene on average, respectively; $P = 0.0016$, Wilcoxon signed-rank test; Figure 2F)". Is this not

expected or given since there are more histone data ABC model can work on in co-activity domains in comparison to background (Supp. Figure 2E)?

We thank the reviewer for their comment. As discussed in the response for comment 1, this could indeed be explained by missed contacts in the background regions resulting from poor signals. We now explicitly state this in the text:

"Similarly, genes located in co-activity domains were on average associated with more enhancers than those in background regions (1.8 versus 1.6 connections per gene on average, respectively; $P = 0.0016$, Mann-Whitney U test; Figure EV1H), although we cannot rule out that these differences are driven by low H3K27ac signal in background regions."

This conclusion could be drawn if the ABC model were to work irrespective of the genomic distance spanned by the PE contact. But ABC model is still skewed (albeit less than the nearest gene model) to shorter interaction distances.

We thank the reviewer for their comment. The ABC method is indeed not independent of distance, since it is calculated by combining HiC with data from H3K27ac and DNase. Since the latter two are not able to account for distance information, it is the HiC that is driving the signal-to-distance decay in interactions, and therefore the final scores should have similar distance-based properties or biases as the HiC input. We further note that it is not unreasonable to assume that most interactions are at shorter distances - this has also been observed experimentally using CRISPRi, which clearly shows the strongest effects for the most proximal targets (Fulco et al, 2019).

The ABC method is, of course, based on predictions and is not without bias, so in addition to the additional analysis based on HiC individuals above, we have cleared up the text to clarify that the ABC scores are only predictions, and that follow-up experiments would be required to confirm the individual interactions.

In the Discussion, we now write:

"We note, however, that our result could also reflect low sensitivities in the called interactions, in part due to differences across individuals in the resolution of input data used for modeling, emphasizing the importance of future experiments to validate differences in enhancer-gene connectivity between individuals and their impact on gene expression levels."

3. Page 9, authors state "the expression of 62 genes showed significant correlation with the number of interactions (Pearson correlation test; BH-adjusted $P < 0.1$), for which the correlation sign was positive in 90% of cases. This suggests that the number of regulatory interactions reflects a gene's expression level within a co-activity domain, but that only few regulatory domains are sensitive to perturbations at an individual level." The enhancer number and gene expression levels might not correlate since they act upon different levels of transcription dynamics. The authors should clarify this point. Also what do authors mean by the last sentence is not clear.

We thank the reviewer for the comment. In general, we cannot observe a significant correlation between the expression of a gene and the corresponding enhancer number for that gene, where we would like to emphasize that the correlation is taken across the set of individuals and 'enhancer number' refers to the fact that some individuals may differ compared to others in terms of predicted (> ABC cutoff) enhancers linked to a given gene. This inability to observe significant effects could be in part due to technical limitations in the ABC method (noise in DNase and/or H3K27ac), which likely play a large role in the context of looking for variation over individuals within the same cell type, which likely have very similar profiles biologically. We do observe, however, 62 cases and were reassured that the direction of correlation was overwhelmingly in the expected direction, giving rise to the statement "only a few regulatory domains are sensitive to perturbations at an individual level". This could indeed be due to different underlying mechanisms of enhancers on an individual level, for example buffering of enhancers could result in very few observed changes in expression.

We have implemented the following changes to the text (pasting relevant snippets below):

- We have made it clear that the correlation per gene is taken over individuals:
  - "We next asked whether the number of interactions per gene per individual was associated with the gene expression of that individual (paired analysis). Overall, the expression of a gene across individuals showed no or only weak correlation with their corresponding number of ABC-connections (PCC = 0.07)."

- We have clarified the point that, while enhancer number and gene expression (from the perspective of looking across different genes) do appear to correlate, we do not necessarily see this for the same gene on an individual level (which could very well be due to buffering mechanisms). We have further made it clear that there are other ways enhancers might relate to target expression beyond their raw numbers, such as activity level, burst-size or buffering.
  - "This might reflect ABC measures being influenced by noise in the input data, or that enhancers might relate to target gene expression in ways beyond their raw numbers. For instance, multiple enhancers for a gene may provide regulatory redundancy (Joshua L Payne, 2015; Perry *et al*, 2010) and, hence, not additively influence gene expression levels. Indeed, the expression of only 62 genes showed significant correlation with the number of interactions (Pearson correlation test; BH-adjusted $P < 0.1$), for which the correlation sign was positive in 90% of cases. Together, these results suggest that the number of regulatory interactions may reflect differences in expression level within a co-activity domain across genes, as previously shown

(Andersson *et al*, 2014), but that only few regulatory domains are sensitive to perturbations at an individual level."

4. Are the percentage differences stated in the last paragraph of page 14 statistically significant?

We thank the reviewer for highlighting left out statistics. Indeed, the percentage differences are statistically significant. We have added the statistics to the main text and Figure 4:

- The number of domains that have at least one co-activity QTL associated between variable and matched non-variable co-activity domains: Fisher's exact test, P = 0.00035
- The percentage of neighboring eGene pairs: Fisher's exact test:
  - Background domains versus co-activity domains: P < 2.2e-16
  - Matched co-activity domains versus variable co-activity domains: P = 0.027

5. It is interesting that co-activity QTLs were associated with larger effect sizes (fig. 5c). This is most likely due to bettering of eQTL-target gene mapping using co-activity domains. The authors could highlight this point.

We thank the reviewer for their comment. While we agree that regulatory interactions are likely to be contained within the same co-activity domain, we would like to point out a possible misunderstanding in terms of our methodology. The co-activity scores are, via transcriptional decomposition, modeled as a portion of total expression. Hence, any genetic variant associated with co-activity (co-activity QTL) should be also associated with total expression (eQTL). However, by disregarding the portion of expression independent from genomic positioning, the co-activity scores are less affected by gene-specific regulatory effects and may, therefore, more reliably capture variation due to genetic perturbation of regulatory processes acting upon co-activity within domains. However, we have not performed a careful investigation of how eQTLs compare to co-activity QTLs, which we believe is out of scope in this study, so we would prefer to avoid making too big conclusions out of this. To make it clear that co-activity QTLs refer to genetic associations with co-activity scores, we have changed the legend of Figure 5C.

6. The co-activity scores seem to capture VCMs albeit at a lower resolution. Could this support that histone mark signatures are not the main driver/cause of VCM domains, but rather the result of a higher-order regulation/process? Authors should elaborate on this in relation with their conclusions.

We thank the reviewer for their interesting comment. It is true that VCMs and variable co-activity domains are both capturing coordinated activity within local genomic neighborhoods. While VCMs are based on histone modifications, the variable co-activity domains from this study are based only on transcriptional activity, and therefore, while likely to be correlated, the two sets are domains that are measured based on different data modalities and computational approaches and therefore worth comparing.

While we want to leave it to the original investigators of the VCMs to claim driving mechanisms, we can speculate on their relationship with co-activity domains. Since expression and histone modifications are correlated, it is expected to see overlaps between the two sets of domains. The major differences in resolution is likely explained by transcriptional decomposition acting on much wider genomic bins, which we believe is allowing us to capture coordinated gene activity within domains, when VCMs act on a smaller scale. It is likely that changes in transcription caused by changes in transcription factor expression, which we find to be the main determinant of variability in co-activity domains, could also drive VCMs. Relatedly, the authors of the related cis-regulatory domains (CRDs) (Delaneau et al, 2019), speculate that coordinated activity of domains on different chromosomes are driven by TF abundances. Hence, we agree with the reviewer that VCMs and CRDs are, at least in part, reflecting coordinated TF regulation within domains. We have expanded the Discussion to reflect these aspects:

> "In general, variable co-activity domains showed strong overlap with co-variable histone PTM domains (VCMs). VCMs were previously identified also in LCLs in a similar population of healthy individuals and, similar to our co-activity domains, are also enriched in chromatin contacts and genetic variants (Waszak et al, 2015). Our work shows that co-activity alone reflects properties captured by profiles of histone modifications from which VCMs are derived, albeit on a broader scale and approached from an alternative angle. While the relationship between histone PTM and expression domains is expected (Andersson & Sandelin, 2020), we speculate that changes in TF expression, which we find to be the main determinant of variability in co-activity domains, could also drive variability in VCMs. This is supported by a model of coordinated activity of cis-regulatory domains (CRDs) being driven by TF abundances (Delaneau et al, 2019)."

7. The authors make the claim that variable co-activity domains could possess unique features that can explain their variability. However, this claim is not well supported. According to one line evidence given by the authors, then it should follow that promoters of genes in variable co-activity domains have differing regulatory grammar than those in stable co-activity domains. Have they tested this?

We thank the reviewer for their comment. Analyzing differences in the potential regulatory mechanisms underlying variable and non-variable co-activity domains is an important objective in our current study, and we summarize our findings below:

- We see differences in transcription factor binding site (TFBS) motifs enriched within variable domains (Figure 4A), including: NFATC1, CEBPB, EP300, BMI1, ATF2, TBX21 and BCL11A, suggesting a unique TF grammar in these domains.
- Furthermore, TFBSs were overall similar amongst the variable domains (Figure 4C), suggesting a common grammar driving co-activity domain variability (which is different from that driving non-variable co-activity domains).

- Variable domains had on average higher numbers of (and more variable) enhancer-promoter interactions as measured by ABC (Figure 3D, and for HiC contacts Figure EV2E,F).
- Variable domains were more enriched in eQTLs (Figure 5), suggesting greater propensity for perturbations in these regions.

To follow up on the request by the Reviewer to analyze differences in promoter grammars between variable and (matched) non-variable co-activity domains, we have performed a new analysis of predicted TFBS enrichments:

> "Finally, we observed specific enrichments of predicted TFBSs (Castro-Mondragon *et al*, 2022) at gene promoters (-2 kb to +200 bp around annotated gene TSSs) in variable and matched non-variable co-activity domains compared to all co-activity domains (Figure EV3F), indicating a different promoter grammar of variable co-activity domains."

EV3F is pasted below for convenience:

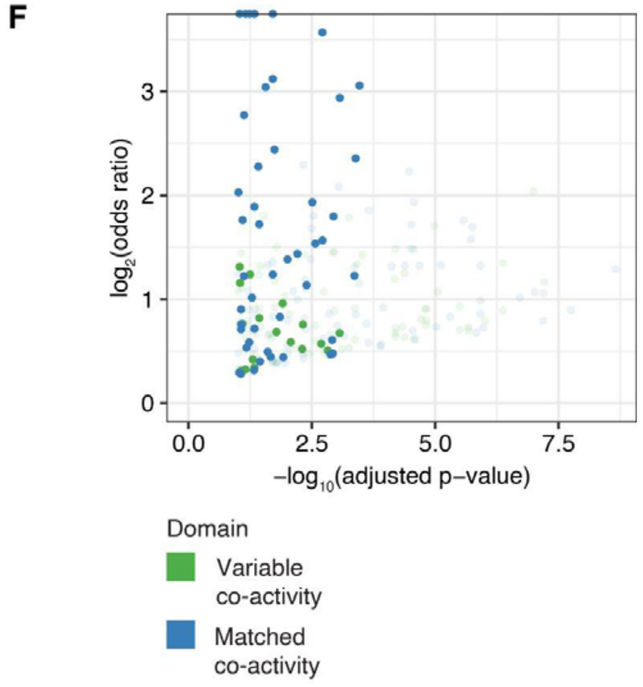

8. In the methods, the model fitting is done using groups of individuals to ease memory demand. Did the authors investigate how using groups of individuals rather than the full set of individuals (but on much smaller number of genomic bins per run (lower resolution)) affect the results?

We thank the reviewer for the question. We can't rule out that there would be a difference if we used all individuals, so this is a potential limitation with our analysis. To overcome computational and memory demands, we are currently developing alternative approaches that are less memory demanding and are looking forward to

presenting these in separate publications. In the Methods section on transcriptional decomposition, we write:

> "In order to facilitate fitting the model, which has a high memory demand, the libraries of expression data of individuals were randomly assigned to groups of between 40 and 50 individuals.
> [...]
> In order to achieve good convergence and maximum comparability across individuals, chromosomes and groups, the hyperparameters of the model were fixed using the following strategy
> [...]
> Whilst efforts were made to make the results as generalizable as possible, we cannot rule out small batch differences impacting our results. Furthermore, we did not investigate the impact of different resolutions (bin sizes) and/or parameterizations of the model itself on the overall results. These aspects could potentially be addressed in future studies."

Minor comments
1. Is supp. Fig 6c correct?

We thank the reviewer for noticing that Supplementary Figure 6C was not correct - it should be TF expression and genotype. We have corrected the supplementary figure (Figure EV4).

2. Is this sentence correct? "Put in another way, we excluded the portion of expression that can be explained by a given gene independently of its neighboring genes." (page 18) It is not clear to me what is it that is excluded.

We thank the reviewer for their comment and hope that our revisions to the description of the method, both in the main text and in the methods, should help clarify this sentence from the Discussion.

In answer to the question, the transcriptional decomposition aims to split expression in bins across chromosomes into two parts - one is co-activity (the main subject of the paper, and whereby a bin-to-bin neighboring relationship is assumed), and the other is the independent component (which assumes that the expression in a given region/gene is independent of its neighbors). It is this independent component that we exclude, keeping only co-activity.

Since the method can be intuitively hard to follow, we have implemented the following changes to the text/figures with regards to the transcriptional decomposition method:

- Expanded upon and clarified the explanation of the transcriptional decomposition in the main text. In particular, we have expanded upon paragraph 1 in the section "Transcriptional decomposition captures

positionally dependent gene co-activities" to include further intuition, which should help the reader to understand conceptually what is being measured:

  - "We have previously established transcriptional decomposition (Rennie et al, 2018), which is a novel Bayesian modeling-based approach for decomposing RNA expression in genomic bins along chromosomes into two parts: the portion of expression attributable to the local genomic neighborhood (positionally dependent (PD) component, referred to henceforth as 'co-activity' in the current study), and the portion of expression independent of the genomic position (positionally independent (PI) component). Formally, we model the log of the normalized (reads per million, RPM) of a given bin as approximately PD + PI, where the value for PD (co-activity) is assumed to be dependent on neighboring bins, whereas the value for PI is assumed to be independent of its neighbors. The co-activity portion is thus highly similar at close-by regions, which we hypothesize could result from a combination of shared regulatory mechanisms as well as similarities in the underlying chromatin environment, whilst the independent portion is uncorrelated with distance and likely reflects gene-specific regulatory mechanisms (Rennie et al, 2018) (Figure 1B)."

- Expanded upon the method in the main text:

  - **Added further mathematical details (see below)**

- Improved upon the overall clarity of Figure 1A, including the following specifics:

  - We have added an equation to more clearly depict that expression is split into two parts, and that one of them (co-activity) is the focus of the paper. We have further included the overall questions the study attempts to answer (top).
  - We have added small barcharts (purple) to better depict stable and variable co-activity domains - that is, according to the variance of the mean co-activity in a domain across the individuals.
  - On the right hand side we have replaced the blue squares that represented transcription factor (TF) abundance with a similar bar chart to depict variation in TF abundance across individuals.

<u>Revised transcriptional decomposition methods section involving more comprehensive mathematical details:</u>

"The transcriptional decomposition model was fit to the 10 kb binned RNA-seq datasets using a previously described approach (Rennie et al, 2018), which is based on a Bayesian hierarchical model that relies on the integrated nested laplace approximation, implemented in the package R-INLA (Rue et al, 2009). Briefly, INLA focuses on models which can be expressed as a conditional Markov random field (a widely used family of models which are particularly suited to modeling the underlying

structural dependencies in data), and make the assumption that the parameter marginal distributions can be approximated using the integrated nested laplace approximation, a step which greatly eases computational processing time and thus suited to large datasets.

Briefly, the transcriptional decomposition approach is as follows: let $y_i \dots y_N$ relate to a chromosomal segment such that y_i represents the total read count in bin i, for a total of N bins in the segment (which could in theory span the whole chromosome, but for modeling purposes we terminate the segments prior to large regions of non-expressed consecutive bins - see below). We assume that these read counts are distributed as negative binomial, such that $y_i \sim NB(s^{-1}\mu_i, \theta)$ where s is the library depth offset (number of millions of reads), μ_i is the mean RPM for bin i and θ is a hyperparameter representing the overdispersion. We model the log of the mean expression in a given bin i as a combination of two latent components and an intercept, in other words $log\ (\mu_i) = log\ (s) + \alpha + PD_i + PI_i$, where α is the intercept and PD_i and PI_i are the positionally dependent (co-activity) and independent components respectively.
The co-activity component was modeled as a first-order random walk, dependent on neighboring bins and assuming normally distributed differences. This equates to the difference between neighboring bins, $PD_i - PD_{i-1}$ having a normal distribution with mean zero and variance $1/\tau_{PD}$, and where for identifiability purposes the component is scaled to $\sum_{i=1}^{N}(PD_i - PD_{i-1}) = 0$.

The positionally independent component was modeled assuming bins to be independent and identically distributed (IID) $(PI_i \sqcup PI_{i-1})$ where PI_i is Guassian with variance $1/\tau_{PI}$. The model was fit as a hierarchical model in INLA, whereby priors for the hyperparameter θ was given a gaussian prior and τ_{PD} and τ_{PI} were fixed according to the scheme described below. For each of the co-activity and independent components, the mean and variance based on samples from modeled posterior was taken for each bin and used in subsequent analyses. "

Reviewer #3:

Based on a method published by the same authors earlier, this study quantifies the gene co-activity across individuals, characterises the variable co-activity domains and identifies TFs as main drivers for co-activity.

Overall, this is a nice study with solid descriptive analyses on co-activity domains. The key finding is that variation in gene co-activity is mostly driven by expression variation of TFs which bind to enhancers in these domains (based on motif enrichment). The variance explained by TF expression is quite impressive, however the significance of the findings in my view remain a bit hidden since the concept of co-activity is relatively hard to grasp and the authors could do a better job introducing it and relating it to known concepts, such as enhancer-based gene regulatory networks.

Major comments:

The concept of co-activity is hard to grasp. Since this study is based on a previously published method it will be important to again define with co-activity means, at least conceptually. It cannot be assumed that the reader knows the previous publication.

Related, I find the schematic in Figure 1A not very clear. What are the purple and grey bars? Why are there no bars in the blue co-activity domain? What are the blue (TF?) squares reflecting?

We thank the reviewer for their positive impression of our work. We agree that the method can be intuitively hard to follow, and in hindsight we should have included more description in order to minimize the requirement of the reader to refer back to the original text describing the method. Therefore, we have implemented the following changes to the text/figures with regards to the transcriptional decomposition method:

- Expanded upon and clarified the explanation of the transcriptional decomposition in the main text. In particular, we have expanded upon paragraph 1 in the section "Transcriptional decomposition captures positionally dependent gene co-activities" to include further intuition, which should help the reader to understand conceptually what is being measured:
    - "We have previously established transcriptional decomposition (Rennie *et al*, 2018), which is a novel Bayesian modeling-based approach for decomposing RNA expression in genomic bins along chromosomes into two parts: the portion of expression attributable to the local genomic neighborhood (positionally dependent (PD) component, referred to henceforth as 'co-activity' in the current study), and the portion of expression independent of the genomic position (positionally independent (PI) component). Formally, we model the log of the normalized (reads per million, RPM) of a given bin as approximately PD + PI, where the value for PD (co-activity) is assumed to be dependent on neighboring bins, whereas the value for PI is assumed to be independent of its neighbors. The co-activity portion is thus highly similar at close-by regions, which we hypothesize could result from a combination of shared regulatory mechanisms as well as similarities in the underlying chromatin environment, whilst the independent portion is uncorrelated with distance and likely reflects gene-specific regulatory mechanisms (Rennie *et al*, 2018) (Figure 1B)."
- Expanded upon the method in the main text:
    - **Added further mathematical details (see below)**
- Improved upon the overall clarity of Figure 1A, including the following specifics:
    - We have added an equation to more clearly depict that expression is split into two parts, and that one of them (co-activity) is the focus of the paper. We have further included the overall questions the study attempts to answer (top).

- We have added small barcharts (purple) to better depict stable and variable co-activity domains - that is, according to the variance of the mean co-activity in a domain across the individuals.
- On the right hand side we have replaced the blue squares that represented transcription factor (TF) abundance with a similar bar chart to depict variation in TF abundance across individuals.

Revised transcriptional decomposition methods section involving more comprehensive mathematical details:

“The transcriptional decomposition model was fit to the 10 kb binned RNA-seq datasets using a previously described approach (Rennie *et al*, 2018), which is based on a Bayesian hierarchical model that relies on the integrated nested laplace approximation, implemented in the package R-INLA (Rue *et al*, 2009). Briefly, INLA focuses on models which can be expressed as a conditional Markov random field (a widely used family of models which are particularly suited to modeling the underlying structural dependencies in data), and make the assumption that the parameter marginal distributions can be approximated using the integrated nested laplace approximation, a step which greatly eases computational processing time and thus suited to large datasets.

Briefly, the transcriptional decomposition approach is as follows: let $y_i \dots y_N$ relate to a chromosomal segment such that y_i represents the total read count in bin i, for a total of N bins in the segment (which could in theory span the whole chromosome, but for modeling purposes we terminate the segments prior to large regions of non-expressed consecutive bins - see below). We assume that these read counts are distributed as negative binomial, such that $y_i \sim NB(s^{-1}\mu_i, \theta)$ where s is the library depth offset (number of millions of reads), μ_i is the mean RPM for bin i and θ is a hyperparameter representing the overdispersion. We model the log of the mean expression in a given bin i as a combination of two latent components and an intercept, in other words $log\,(\mu_i) = log\,(s) + \alpha + PD_i + PI_i$, where α is the intercept and PD_i and PI_i are the positionally dependent (co-activity) and independent components respectively.
The co-activity component was modeled as a first-order random walk, dependent on neighboring bins and assuming normally distributed differences. This equates to the difference between neighboring bins, $PD_i - PD_{i-1}$ having a normal distribution with mean zero and variance $1/\tau_{PD}$, and where for identifiability purposes the component is scaled to $\sum_{i=1}^{N}(PD_i - PD_{i-1}) = 0$.

The positionally independent component was modeled assuming bins to be independent and identically distributed (IID) $(PI_i \sqcup PI_{i-1})$ where PI_i is Guassian with variance $1/\tau_{PI}$. The model was fit as a hierarchical model in INLA, whereby priors for the hyperparameter θ was given a gaussian prior and τ_{PD} and τ_{PI} were fixed according to the scheme described below. For each of the co-activity and independent components, the mean and variance based on samples from modeled posterior was taken for each bin and used in subsequent analyses. “

P15 It would be important to assess also the importance of see chromatin marks, even if they are not necessarily direct.

We thank the reviewer for their comment. We have added an extra supplementary figure (Figure EV4E), which includes histone modifications as an added predictor

group to the model. This shows that transcription factors are still strong at explaining co-activity levels after accounting for histone modifications.

In the final part where authors link TFs to co-regulated domains, it seems they are essentially reconstructing a enhancer-based gene regulatory network. If this interpretation is correct, it would be good to relate their findings to recent developments in enhancer-based gene regulatory networks (Glue, Granie, SCENIC+, Pando, to name a few).

While the analysis of TFs in our study does not specifically focus on TFBSs at enhancers, it is likely that some of the TF associations we reveal in our study could also be inferred from enhancer-based GRNs. However, there is a clear distinction, as the inference of co-activity domains relies on transcriptional decomposition and most GRNs are based on correlations. Nevertheless, we have expanded the Discussion to place our work in the context of enhancer-based GRNs:

"Our results reveal that, relative to local effects of eQTLs and ABC-predicted interactions, TF abundance is the strongest driver of co-activity variability. This concurs with previous observations that TF abundance influences coordinated variability (Delaneau *et al*, 2019). The strong association between TF expression variability and variability in domain co-activity lead us to hypothesize that variable co-activity domains, at least partly, reflect enhancer-based gene regulatory networks (Kamal *et al*, 2021; González-Blas *et al*, 2022), and that local genotype variation in cis to TF genes drives trans-effects on co-activity in variable domains through altered binding to their regulatory elements."

The explanatory power of TFs seems very large (~60% variance explained by TFs), is that comparable to explaining expression levels (i.e. how much variance of expression variation would be explained by the same TFs?)? How does this compare to the level explained for genes are not in co-activity domains?

We thank the reviewer for their comment. We do expect the variance explained by TFs to also be high when looking at raw expression levels. This is because co-activity and expression are related. However, the co-activity is the portion of the raw expression that is 'enriched' for co-activity effects. Therefore, the explanatory power of TFs should be larger, under the hypothesis that we expect TF binding in a region to have a co-regulatory effect on multiple genes in that region.

In order to address this analytically, we have analyzed the explanatory power of TFs on expression levels versus co-activity, in variable co-activity domains. Overall, the set of TFs explain more of the variance in co-activity than the variance in the raw expression levels for the same gene. This suggests that the positionally independent portion of expression may be more influenced by local effects, such as histone modifications, and by separating the two from total expression, we can better capture how TFs influence gene co-regulation. This figure (shown below) is now included as Figure EV4 E-F in the revised version of the manuscript.

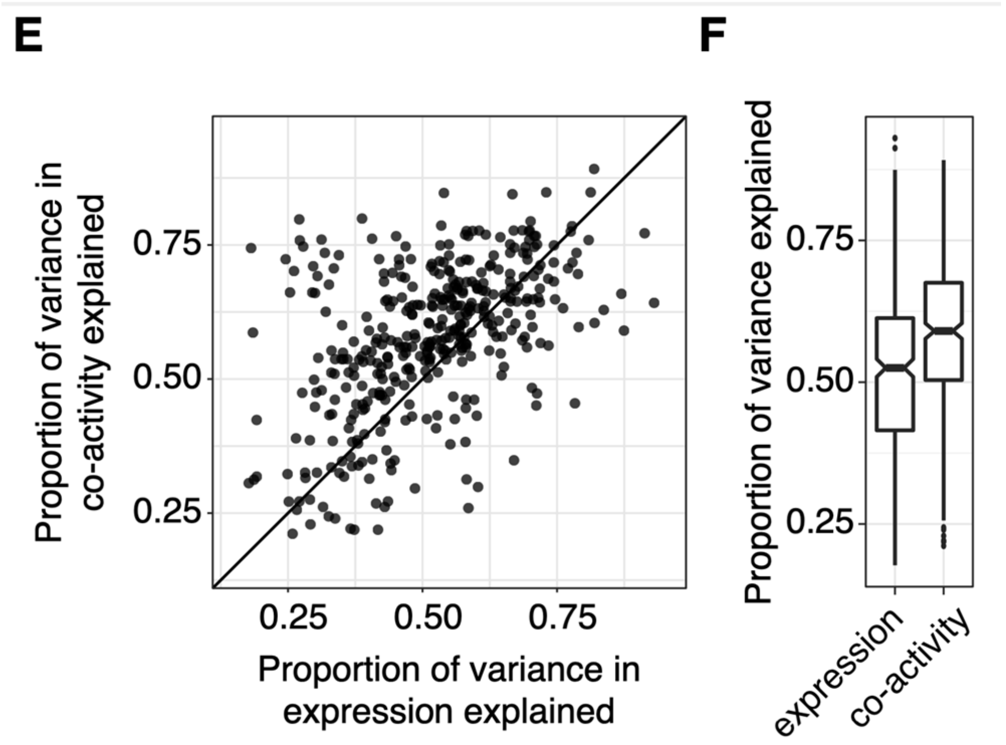

Have the authors tried how much variance of the co-activity domains is explained by the SNP that regulates the TF? (i.e. taking Figure 6C one step further)

> This is a good suggestion. We have tried to do this, and found that a single SNP in the vicinity of the TF does explain a small proportion of the variation in the co-activity domain. We note here, however, that in order to detect these differences as significant would require much greater statistical power than we have in the current study. A given TF is likely to be influenced by a large number of variants, and a given variant may impact multiple TFs. Furthermore, a single TF might in turn impact multiple domains. Therefore, it is very difficult to pinpoint a given variant near a TF to changes in distal co-activity. We do, however, think that it is an extremely interesting question, and perhaps one which could be addressed in future studies. We do, however, think that our results demonstrate the utility of modeling TF expression as a proxy for distal trans effects alongside efforts to understand the genetic basis of expression variation in health and disease.

Clarifications:

The description in the legend of Figure 1B is difficult to understand. This should be clarified in the methods section (and also mentioned in the main text)

> We apologize for the lack of clarity in the text - we have implemented the following changes:

- Changed the figure legend in 1B to say:

  "Overall strategy of how expression data from each sample is decomposed into transcriptional components. Via approximate Bayesian modeling, normalized RNA expression count data (reads per million, RPM), quantified in 10 kb genomic bins (shown left for three individuals), is modeled as approximately PD + PI, where the value for PD (positionally dependent component: co-activity) is assumed to be dependent on neighboring bins, whereas the value for PI (positionally independent component) is assumed to be independent of its neighbors (shown right). The co-activity (PD) component is modeled as a first-order random walk (see Methods)."

- Significantly expanded the description of transcriptional decomposition in the Methods section (as described in our response above).

Figure 1C: how many gene pairs are there? Why are some tiles empty? Can you add p-values to judge significance? Also, is this result expected just based on higher variance will lead to stronger correlation?

> We thank the reviewer for their comment. We have now added the number of gene pairs in each cell of Figure 1D (old Figure 1C), which we believe adds to the interpretability of the plot. The tiles which are empty are those where there are either no or fewer than 10 gene pairs (a total of 4 tiles in the latter case).

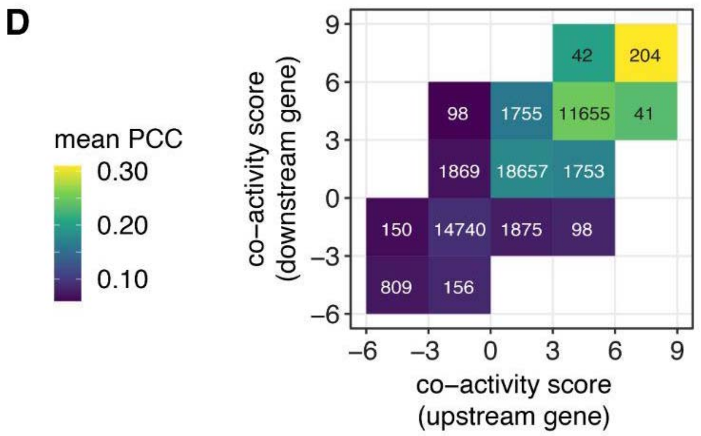

> In addition to the above, we have also added Figure EV1A, which shows the proportion of gene pairs in a tile which had significant co-expression (adjusted p-value<0.1).

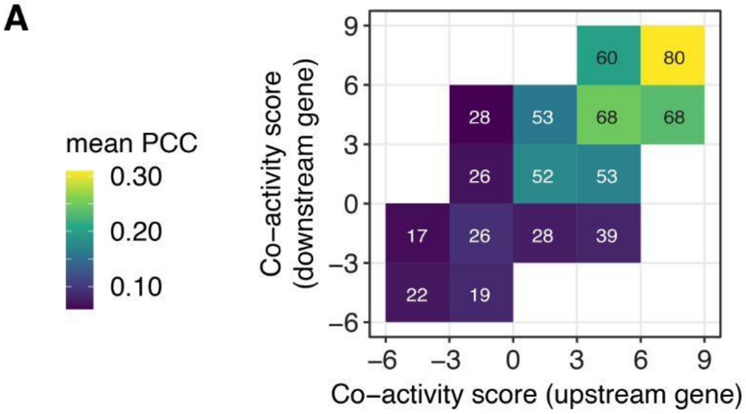

Furthermore, we have altered the main text to reflect the fact that this is indeed not an unexpected result.

"co-activity domains were on average associated with more enhancers than those in background regions (1.8 versus 1.6 connections per gene on average, respectively; P = 0.0016, Wilcoxon signed-rank test; Figure 2F)": Figure 2F seems to be showing something else (eQTLs)

We thank the reviewer for spotting this - we have revised the text, which now accurately points to Figure EV1H.

The authors should show some data for these statements: "On average, an individual's expression for a gene showed no or only weak correlation with its corresponding number of ABC-connections (PCC = 0.07). While this might reflect ABC measures being influenced by noise in the input data, the expression of 62 genes showed significant correlation with the number of interactions (Pearson correlation test; BH-adjusted P < 0.1), for which the correlation sign was positive in 90% of cases. This suggests that the number of regulatory interactions reflects a gene's expression level within a co-activity domain, but that only few regulatory domains are sensitive to perturbations at an individual level."

We thank the Reviewer for this suggestion. We have added a supplementary figure (Figure EV1I, pasted below) to illustrate the data underlying these results.

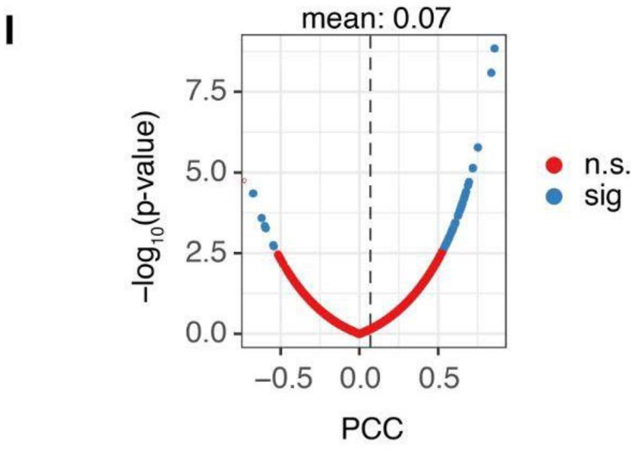

Claiming that Supplementary Figure 5 shows a clustering by genotype is an overstatement, at most the Utah population is partially separated.

We agree with the reviewer and have changed the text to tone down this claim:

"Principal component analysis revealed a modest separation by ancestry of the individuals for the co-activity scores, but less so for the positionally independent component or the raw expression data (Appendix Figure S2), suggesting that individual genetic variation may influence individual differences in the regulation of gene co-activities."

Technical questions:

"Similarly, genes located in co-activity domains were on average associated with more enhancers than those in background regions": is this expected based on the analysis? i.e. both, co-activity and ABC-associated enhancers are based on correlation?

While we agree that more enhancers are likely to be contained and linked with genes within co-activity domains than outside of co-activity domains, we would like to point out a possible misunderstanding in terms of our methodology. The co-activity scores are, via transcriptional decomposition, modeled as a portion of total expression and not based on correlation. Hence, we would not expect the number of ABC connections to be technically linked with the co-activity scores, as they are derived from different data modalities and using different methodologies. We have updated the text:

"In addition, genes within co-activity domains were generally less variable in their number of ABC-associated enhancers (P = 1.1×10-8; Mann-Whitney U test; Figure EV1H). This is likely in part explained by their association with higher gene expression levels, since more variably interacting genes had lower median expression across individuals (Spearman's rho -0.46; P < 2×10-16; Figure EV1G). Similarly, genes located in co-activity domains were on average associated with more enhancers than those in background regions (1.8 versus 1.6 connections per gene on average, respectively; P = 0.0016, Mann-Whitney U test; Figure EV1H), although we cannot rule out that these differences are driven by low H3K27ac signal in background regions."

Page 12: Of 83 tested TFs (Supplementary Table 5) with experimentally identified binding sites in LCL. It would be great to see how this would perform when using predicted binding sites. This would be good to see, since it would determine whether the approach can be applied to cell types with less ChIP-seq data available.

This is a good suggestion. We have added additional analyses using predicted TFBSs (Figure EV3A), repeating the analyses previously shown in Figure 4B for experimentally identified TFBSs:

“Interestingly, the more enriched the TFBSs for a given TF were within variable co-activity domains, the more variable we observed the expression of the TF itself to be (PCC: 0.34, Pearson correlation test, P = 0.0017, Figure 4B). The same pattern was observed for predicted TFBSs derived from TF motif scanning (Castro-Mondragon et al, 2022) (PCC: 0.27, Pearson correlation test, P = 1.2×10-10; Figure EV3A), and for experimentally defined TFBSs for the matched non-variable co-activity domains (PCC: 0.25, Pearson correlation test, P = 0.022; Figure EV3B), although the associations were weaker.”

Figure 2G: what is n in the legend? what is x axis?

We thank the reviewer for pointing out missing information. We have updated Figure 2G and the legend to state that the x axis denotes the distance between eGenes and n denotes the number of genes in each distance bin.

Figure 3B: what are ABCDEF in the x- and y-axis?

We apologize for the lack of clarity regarding what these genes are - we have now added the genes to the figure itself (instead of only in the figure placed to the right). Please see the revised figure below. Note that we have redone Figures 3A & B using a new locus. This is because both of the genes in the previous version of the figure were lncRNAs that divergently overlap the 3’ end of the PTPRB gene, and in hindsight (and in response to a comment by Reviewer 1), it might have been better to select a locus with known mRNA genes.

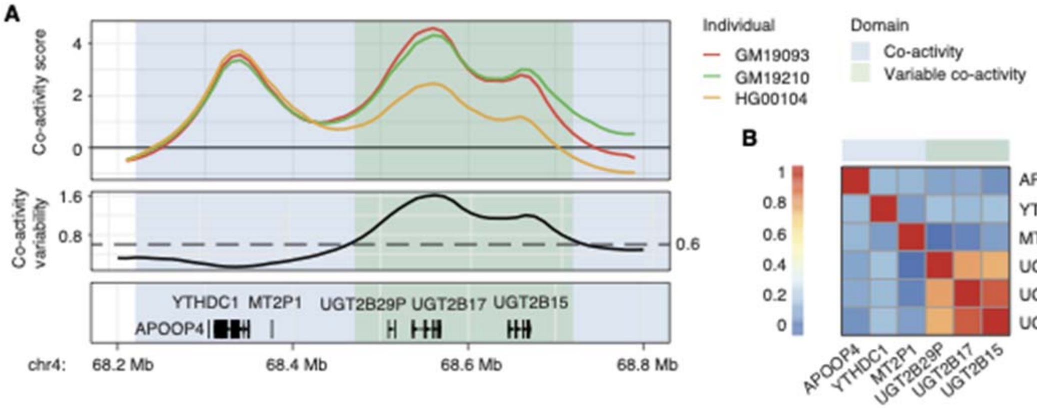

minor:
page 4: "we modeled the positionally dependent portion of expression data independently in each LCL using aggregated expression in 10 kb tiled windows of the genome (Figure 1B)." How robust are the findings to changing the 10 kb window size?

We thank the reviewer for their comment, this is an important point. In this study, we aimed to adapt the transcriptional decomposition method to RNA-seq data by controlling the precision hyperparameters to most closely match CAGE-derived transcriptional components of GM12878 (Rennie et al, 2018) and therefore also kept window sizes of the original study. It should be noted that while changing the window

sizes may result in different co-activity estimates, there is a clear trade-off between resolution and capturing co-activity of neighboring genes. Through a series of analyses (Rennie et al, 2018), we have established 10 kb as a useful window size for capturing the underlying (known) chromatin biology, but acknowledge that it would be interesting to more systematically investigate the impact of varying (or adaptive) window sizes in a future study. We have added the following statement to the Methods section on transcriptional decomposition:

> "Whilst efforts were made to make the results as generalizable as possible, we cannot rule out small batch differences impacting our results. Furthermore, we did not investigate the impact of different resolutions (bin sizes) and/or parameterizations of the model itself on the overall results. These aspects could potentially be addressed in future studies."

References cited in the responses

Andersson R, Gebhard C, Miguel-Escalada I, Hoof I, Bornholdt J, Boyd M, Chen Y, Zhao X, Schmidl C, Suzuki T, *et al* (2014) An atlas of active enhancers across human cell types and tissues. *Nature* 507: 455–461

Castro-Mondragon JA, Riudavets-Puig R, Rauluseviciute I, Berhanu Lemma R, Turchi L, Blanc-Mathieu R, Lucas J, Boddie P, Khan A, Manosalva Pérez N, *et al* (2022) JASPAR 2022: the 9th release of the open-access database of transcription factor binding profiles. *Nucleic Acids Res* 50: D165–D173

Delaneau O, Zazhytska M, Borel C, Giannuzzi G, Rey G, Howald C, Kumar S, Ongen H, Popadin K, Marbach D, *et al* (2019) Chromatin three-dimensional interactions mediate genetic effects on gene expression. *Science* 364: eaat8266

Fulco CP, Nasser J, Jones TR, Munson G, Bergman DT, Subramanian V, Grossman SR, Anyoha R, Doughty BR, Patwardhan TA, *et al* (2019) Activity-by-contact model of enhancer–promoter regulation from thousands of CRISPR perturbations. *Nat Genet* 51: 1664–1669

González-Blas CB, Winter SD, Hulselmans G, Hecker N, Matetovici I, Christiaens V, Poovathingal S, Wouters J, Aibar S & Aerts S (2022) SCENIC+: single-cell multiomic inference of enhancers and gene regulatory networks. 2022.08.19.504505 doi:10.1101/2022.08.19.504505 [PREPRINT]

Gorkin DU, Qiu Y, Hu M, Fletez-Brant K, Liu T, Schmitt AD, Noor A, Chiou J, Gaulton KJ, Sebat J, *et al* (2019) Common DNA sequence variation influences 3-dimensional conformation of the human genome. *bioRxiv* 28: 592741

Joshua L Payne AW (2015) Mechanisms of mutational robustness in transcriptional regulation. *Front Genet* 6: 378

Kamal A, Arnold C, Claringbould A, Moussa R, Daga N, Nogina D, Kholmatov M, Servaas N, Mueller-Dott S, Reyes-Palomares A, *et al* (2021) GRaNIE and GRaNPA: Inference and evaluation of enhancer-mediated gene regulatory networks applied to study macrophages

Perry MW, Boettiger AN, Bothma JP & Levine M (2010) Shadow enhancers foster robustness of Drosophila gastrulation. *Curr Biol CB* 20: 1562–1567

Rennie S, Dalby M, van Duin L & Andersson R (2018) Transcriptional decomposition reveals active chromatin architectures and cell specific regulatory interactions. *Nat Commun* 9: 487

Rue H, Martino S & Chopin N (2009) Approximate Bayesian inference for latent Gaussian models by using integrated nested Laplace approximations. *J R Stat Soc Ser B Stat Methodol* 71: 319–392

11th Apr 2023

Manuscript Number: MSB-2022-11392R, Transcription factor expression is the main determinant of variability in gene co-activity

Dear Dr Andersson,

Thank you for sending us your revised manuscript. My colleague Jingyi Hou is on maternity leave and I took over handling your manuscript. We have now heard back from the three reviewers who were asked to evaluate your revised study. As you will see below, the reviewers are satisfied with the performed revisions and support publication. Before we can formally accept the manuscript for publication, we would ask you to address some remaining editorial issues listed below.

- Our data editors have noted some missing information in the figure legends, please see the attached .doc file. Please make all requested text changes using the attached file and *keeping the "track changes" mode* so that we can easily access the edits made.

- Please include 5 keywords.

- Please include a "Disclosure and Competing Interests Statement" in the main text.

- Figure 1A should be called out before Figure 1B in the main text.

- Appendix Tables S1-S5 should be provided as EV Datasets. Please upload them individually (one file per dataset) and rename them to Dataset EV1-EV5. The callouts in the text should be updated accordingly. Their descriptions should be removed from the Appendix file. The description of each EV dataset should be included in the respective xls file in a separate sheet.

- Please include page numbers in the Appendix Table of Contents.

- Appendix Figure S3 should be included in the Appendix Table of Contents.

Please resubmit your revised manuscript online, with a covering letter listing amendments and responses to each point raised by the referees. Please resubmit the paper **within one month** and ideally as soon as possible. If we do not receive the revised manuscript within this time period, the file might be closed and any subsequent resubmission would be treated as a new manuscript. Please use the Manuscript Number (above) in all correspondence.

Click on the link below to submit your revised paper.

https://msb.msubmit.net/cgi-bin/main.plex

As a matter of course, please make sure that you have correctly followed the instructions for authors as given on the submission website.

Thank you for submitting this paper to Molecular Systems Biology.

Kind regards,

Maria

Maria Polychronidou, PhD
Senior Editor
Molecular Systems Biology

If you do choose to resubmit, please click on the link below to submit the revision online before 11th May 2023.

https://msb.msubmit.net/cgi-bin/main.plex

IMPORTANT:
Please note that corresponding authors are required to supply an ORCID ID for their name upon submission of a revised manuscript (EMBO Press signed a joint statement to encourage ORCID adoption).
(https://www.embopress.org/page/journal/17444292/authorguide#editorialprocess)

Currently, our records indicate that the ORCID for your account is 0000-0003-1516-879X.

Please click the link below to modify this ORCID:
Link Not Available

The system will prompt you to fill in your funding and payment information. This will allow Wiley to send you a quote for the article processing charge (APC) in case of acceptance. This quote takes into account any reduction or fee waivers that you may be eligible for. Authors do not need to pay any fees before their manuscript is accepted and transferred to the publisher.

*** PLEASE NOTE *** As part of the EMBO Press transparent editorial process initiative (see our Editorial at https://dx.doi.org/10.1038/msb.2010.72 , Molecular Systems Biology will publish online a Review Process File to accompany accepted manuscripts. When preparing your letter of response, please be aware that in the event of acceptance, your cover letter/point-by-point document will be included as part of this File, which will be available to the scientific community. More information about this initiative is available in our Instructions to Authors. If you have any questions about this initiative, please contact the editorial office (msb@embo.org).

Reviewer #1:

Authors have adequately addressed my previous concerns.

Reviewer #2:

The authors have addressed all my comments adequately and I do not have any further edits/comments.

Reviewer #3:

The authors have done a good job addressing my original comments. I have nothing more to add.

We are very pleased to see that the reviewers are satisfied with our revisions and would like to thank all three reviewers for putting their valuable time and thought into reviewing our manuscript.

Reviewer #1:

Authors have adequately addressed my previous concerns.

Reviewer #2:

The authors have addressed all my comments adequately and I do not have any further edits/comments.

Reviewer #3:

The authors have done a good job addressing my original comments. I have nothing more to add.

17th Apr 2023

Manuscript number: MSB-2022-11392RR
Title: Transcription factor expression is the main determinant of variability in gene co-activity

Dear Dr. Andersson,

Thank you again for sending us your revised manuscript. We are now satisfied with the modifications made and I am pleased to inform you that your paper has been accepted for publication.

*** PLEASE NOTE *** As part of the EMBO Publications transparent editorial process initiative (see our Editorial at https://dx.doi.org/10.103/msb.2010.72), Molecular Systems Biology publishes online a Review Process File with each accepted manuscripts. This file will be published in conjunction with your paper and will include the anonymous referee reports, your point- by-point response and all pertinent correspondence relating to the manuscript. If you do NOT want this File to be published, please inform the editorial office at msb@embo.org within 14 days upon receipt of the present letter.

LICENSE AND PAYMENT:
All articles published in Molecular Systems Biology are fully open access: immediately and freely available to read, download and share.

Molecular Systems Biology charges an article processing charge (APC) to cover the publication costs. You, as the corresponding author for this manuscript, should have already received a quote with the article processing fee separately. Please let us know in case this quote has not been received.

Once your article is at Wiley for editorial production, you will receive an email from Wiley's Author Services system, which will ask you to log in and will present you with the publication license form for completion. Within the same system the publication fee can be paid by credit card, an invoice or pro forma can be requested.

Payment of the publication charge and the signed Open Access Agreement form must be received before the article can be published online.

Upon acceptance it is mandatory for you to return the completed payment form. Failure to send back the form may result in a delay in the publication of your paper.

Molecular Systems Biology articles are published under the Creative Commons licence CC BY, which facilitates the sharing of scientific information by reducing legal barriers, while mandating attribution of the source in accordance to standard scholarly practice.

Proofs will be forwarded to you within the next 2-3 weeks.

Thank you very much for submitting your work to Molecular Systems Biology.

Kind regards,

Maria

Maria Polychronidou, PhD
Senior Editor
Molecular Systems Biology

EMBO Press Author Checklist

Corresponding Author Name: Sarah Rennie & Robin Andersson
Journal Submitted to: Molecular Systems Biology
Manuscript Number: MSB-2022-11392

USEFUL LINKS FOR COMPLETING THIS FORM
The EMBO Journal - Author Guidelines
EMBO Reports - Author Guidelines
Molecular Systems Biology - Author Guidelines
EMBO Molecular Medicine - Author Guidelines

Reporting Checklist for Life Science Articles (updated January
This checklist is adapted from Materials Design Analysis Reporting (MDAR) Checklist for Authors. MDAR establishes a minimum set of requirements in transparent reporting in the life sciences (see Statement of Task: 10.31222/osf.io/9sm4x). Please follow the journal's guidelines in preparing your
Please note that a copy of this checklist will be published alongside your article.

Abridged guidelines for figures
1. Data
The data shown in figures should satisfy the following conditions:
- the data were obtained and processed according to the field's best practice and are presented to reflect the results of the experiments in an accurate and unbiased manner.
- ideally, figure panels should include only measurements that are directly comparable to each other and obtained with the same assay.
- plots include clearly labeled error bars for independent experiments and sample sizes. Unless justified, error bars should not be shown for technical
- if n<5, the individual data points from each experiment should be plotted. Any statistical test employed should be justified.
- Source Data should be included to report the data underlying figures according to the guidelines set out in the authorship guidelines on Data

2. Captions
Each figure caption should contain the following information, for each panel where they are relevant:
- a specification of the experimental system investigated (eg cell line, species name).
- the assay(s) and method(s) used to carry out the reported observations and measurements.
- an explicit mention of the biological and chemical entity(ies) that are being measured.
- an explicit mention of the biological and chemical entity(ies) that are altered/varied/perturbed in a controlled manner.
- the exact sample size (n) for each experimental group/condition, given as a number, not a range;
- a description of the sample collection allowing the reader to understand whether the samples represent technical or biological replicates (including how many animals, litters, cultures, etc.).
- a statement of how many times the experiment shown was independently replicated in the laboratory.
- definitions of statistical methods and measures:
  - common tests, such as t-test (please specify whether paired vs. unpaired), simple χ2 tests, Wilcoxon and Mann-Whitney tests, can be unambiguously identified by name only, but more complex techniques should be described in the methods section;
  - are tests one-sided or two-sided?
  - are there adjustments for multiple comparisons?
  - exact statistical test results, e.g., P values = x but not P values < x;
  - definition of 'center values' as median or average;
  - definition of error bars as s.d. or s.e.m.

Please complete ALL of the questions below.
Select "Not Applicable" only when the requested information is not relevant for your study.

Materials

Newly Created Materials	Information included in the manuscript?	In which section is the information available? (Reagents and Tools Table, Materials and Methods, Figures, Data Availability Section)
New materials and reagents need to be available; do any restrictions apply?	Not Applicable	

Antibodies	Information included in the manuscript?	In which section is the information available? (Reagents and Tools Table, Materials and Methods, Figures, Data Availability Section)
For **antibodies** provide the following information:<br>- Commercial antibodies: RRID (if possible) or supplier name, catalogue number and or/clone number<br>- Non-commercial: RRID or citation	Not Applicable	

DNA and RNA sequences	Information included in the manuscript?	In which section is the information available? (Reagents and Tools Table, Materials and Methods, Figures, Data Availability Section)
**Short novel DNA or RNA including primers, probes**: provide the sequences.	Not Applicable	

Cell materials	Information included in the manuscript?	In which section is the information available? (Reagents and Tools Table, Materials and Methods, Figures, Data Availability Section)
**Cell lines:** Provide species information, strain. Provide accession number in repository **OR** supplier name, catalog number, clone number, and/**OR** RRID.	Not Applicable	
**Primary cultures:** Provide species, strain, sex of origin, genetic modification status.	Not Applicable	
Report if the cell lines were recently **authenticated** (e.g., by STR profiling) and tested for mycoplasma contamination.	Not Applicable	

Experimental animals	Information included in the manuscript?	In which section is the information available? (Reagents and Tools Table, Materials and Methods, Figures, Data Availability Section)
**Laboratory animals or Model organisms:** Provide species, strain, sex, age, genetic modification status. Provide accession number in repository **OR** supplier name, catalog number, clone number, **OR** RRID.	Not Applicable	
**Animal observed in or captured from the field:** Provide species, sex, and age where possible.	Not Applicable	
Please detail **housing and husbandry conditions**.	Not Applicable	

Plants and microbes	Information included in the manuscript?	In which section is the information available? (Reagents and Tools Table, Materials and Methods, Figures, Data Availability Section)
**Plants:** provide species and strain, ecotype and cultivar where relevant, unique accession number if available, and source (including location for collected wild specimens).	Not Applicable	
**Microbes:** provide species and strain, unique accession number if available, and source.	Not Applicable	

Human research participants	Information included in the manuscript?	In which section is the information available? (Reagents and Tools Table, Materials and Methods, Figures, Data Availability Section)
If collected and within the bounds of privacy constraints report on age, sex and gender or ethnicity for all study participants.	Not Applicable	

Core facilities	Information included in the manuscript?	In which section is the information available? (Reagents and Tools Table, Materials and Methods, Figures, Data Availability Section)
If your work benefited from core facilities, was their service mentioned in the acknowledgments section?	Not Applicable	

Design

Study protocol	Information included in the manuscript?	In which section is the information available? (Reagents and Tools Table, Materials and Methods, Figures, Data Availability Section)
If study protocol has been **pre-registered, provide DOI in the manuscript**. For clinical trials, provide the trial registration number **OR** cite DOI.	Not Applicable	
Report the **clinical trial registration number** (at ClinicalTrials.gov or equivalent), where applicable.	Not Applicable	

Laboratory protocol	Information included in the manuscript?	In which section is the information available? (Reagents and Tools Table, Materials and Methods, Figures, Data Availability Section)
Provide DOI OR other citation details if **external detailed step-by-step protocols** are available.	Not Applicable	

Experimental study design and statistics	Information included in the manuscript?	In which section is the information available? (Reagents and Tools Table, Materials and Methods, Figures, Data Availability Section)
Include a statement about **sample size** estimate even if no statistical methods were used.	Yes	Results, Materials and Methods, Figure captions
Were any steps taken to minimize the effects of subjective bias when allocating animals/samples to treatment (e.g. **randomization procedure**)? If yes, have they been described?	Not Applicable	
Include a statement about **blinding** even if no blinding was done.	Not Applicable	
Describe **inclusion/exclusion criteria** if samples or animals were excluded from the analysis. Were the criteria pre-established?<br><br>If sample or data points were omitted from analysis, report if this was due to attrition or intentional exclusion and provide justification.	Yes	Materials and Methods
For every figure, are **statistical tests** justified as appropriate? Do the data meet the assumptions of the tests (e.g., normal distribution)? Describe any methods used to assess it. Is there an estimate of variation within each group of data? Is the variance similar between the groups that are being statistically compared?	Yes	Results, Materials and Methods, Figure captions

Sample definition and in-laboratory replication	Information included in the manuscript?	In which section is the information available? (Reagents and Tools Table, Materials and Methods, Figures, Data Availability Section)
In the figure legends: state number of times the experiment was **replicated** in laboratory.	Not Applicable	
In the figure legends: define whether data describe **technical or biological replicates**.	Yes	Figure captions

Ethics

Ethics	Information included in the manuscript?	In which section is the information available? (Reagents and Tools Table, Materials and Methods, Figures, Data Availability Section)
Studies involving **human participants**: State details of **authority granting ethics approval** (IRB or equivalent committee(s), provide reference number for approval.	Not Applicable	
Studies involving **human participants**: Include a statement confirming that **informed consent** was obtained from all subjects and that the experiments conformed to the principles set out in the WMA Declaration of Helsinki and the Department of Health and Human Services Belmont Report.	Not Applicable	
Studies involving **human participants:** For publication of **patient photos**, include a statement confirming that consent to publish was obtained.	Not Applicable	
Studies involving experimental **animals**: State details of **authority granting ethics approval** (IRB or equivalent committee(s), provide reference number for approval. Include a statement of compliance with ethical regulations.	Not Applicable	
Studies involving **specimen and field samples:** State if relevant **permits** obtained, provide details of authority approving study; if none were required, explain why.	Not Applicable	

Dual Use Research of Concern (DURC)	Information included in the manuscript?	In which section is the information available? (Reagents and Tools Table, Materials and Methods, Figures, Data Availability Section)
Could your study fall under dual use research restrictions? Please check biosecurity documents and list of **select agents and toxins** (CDC): https://www.selectagents.gov/sat/list.htm	Not Applicable	
If you used a select agent, is the security level of the lab appropriate and reported in the manuscript?	Not Applicable	
If a study is subject to dual use research of concern regulations, is the name of the **authority granting approval and reference number** for the regulatory approval provided in the manuscript?	Not Applicable	

Reporting

The MDAR framework recommends adoption of discipline-specific guidelines, established and endorsed through community initiatives. Journals have their own policy about requiring specific guidelines and recommendations to complement MDAR.

Adherence to community standards	Information included in the manuscript?	In which section is the information available? (Reagents and Tools Table, Materials and Methods, Figures, Data Availability Section)
State if relevant guidelines or checklists (e.g., **ICMJE, MIBBI, ARRIVE, PRISMA**) have been followed or provided.	Not Applicable	
For **tumor marker prognostic studies,** we recommend that you follow the **REMARK** reporting guidelines (see link list at top right). See author guidelines, under 'Reporting Guidelines'. Please confirm you have followed these guidelines.	Not Applicable	
For **phase II and III randomized controlled trials**, please refer to the **CONSORT** flow diagram (see link list at top right) and submit the CONSORT checklist (see link list at top right) with your submission. See author guidelines, under 'Reporting Guidelines'. Please confirm you have submitted this list.	Not Applicable	

Data Availability

Data availability	Information included in the manuscript?	In which section is the information available? (Reagents and Tools Table, Materials and Methods, Figures, Data Availability Section)
Have **primary datasets** been deposited according to the journal's guidelines (see 'Data Deposition' section) and the respective accession numbers provided in the Data Availability Section?	Not Applicable	
Were **human clinical and genomic datasets** deposited in a public access-controlled repository in accordance to ethical obligations to the patients and to the applicable consent agreement?	Not Applicable	
Are **computational models** that are central and integral to a study available without restrictions in a machine-readable form? Were the relevant accession numbers or links provided?	Yes	Data Availability Section
If publicly available data were reused, provide the respective **data citations in the reference list**.	Yes	References
